# Palladium-catalyzed C–H olefination of uridine, deoxyuridine, uridine monophosphate and uridine analogues†

**DOI:** 10.1039/d2ra03681a

**Published:** 2022-09-01

**Authors:** Qin Zhao, Ruoqian Xie, Yuxiao Zeng, Wanlu Li, Guolan Xiao, Yangyan Li, Gang Chen

**Affiliations:** Shanghai Key Laboratory for Molecular Engineering of Chiral Drugs, School of Chemistry and Chemical Engineering, Shanghai Jiao Tong University Shanghai 200240 People's Republic of China yangany_li@sjtu.edu.cn gchen2018@sjtu.edu.cn; College of Chemistry and Bioengineering, Hunan University of Science and Engineering Yongzhou 425199 People's Republic of China; School of Chemistry and Chemical Engineering, Southeast University Nanjing 211189 People's Republic of China

## Abstract

The palladium-catalyzed oxidative C–H olefinations of uridine, deoxyuridine, uridine monophosphate and uridine analogues are reported herein. This protocol provides an efficient, atom-economic and environmentally friendly approach to the synthesis of biologically important C5-alkene modified uracil/uridine-containing derivatives and pharmaceutical candidates.

## Abstract 2

The palladium-catalyzed oxidative C–H olefinations of uridine, deoxyuridine, uridine monophosphate and uridine analogues are reported herein.

## Graphical abstract

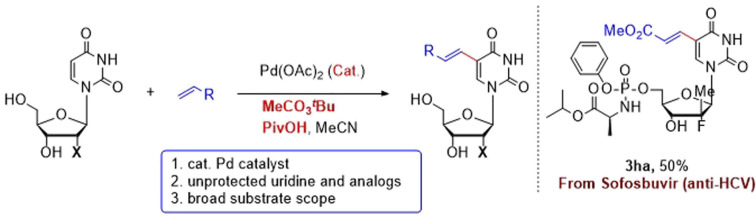

The modifications of nucleosides, nucleotides and oligonucleotide analogs have drawn long-standing interest because of the important scientific significance and various application values in different areas. The chemically modified nucleoside and nucleotide analogues often serve as antitumor and antiviral drugs^1^ in clinical settings to cure cancers and diseases caused by viruses, like the herpes virus, hepatitis virus, AIDS, *etc*. Construction of a compound library of structurally novel and diverse nucleoside analogs (herein, the nucleoside, nucleotide and base analogs are collectively called nucleoside analogs) *via* selective modification of natural nucleosides will help a lot to advance the process of innovative drug development and discovery.^2^ Meanwhile, the base-modified nucleoside analogs with fluorescent groups recently have also emerged as bioorthogonal chemical probes to investigate the nucleic acid structures, activities, locations and interactions, which are extraordinarily useful tools to facilitate understanding of the RNA/DNA functions at the molecular-level.^3^ Apart from this, they can be used to study the mechanisms of carcinogenesis.^4^

As a member of nucleobases uracil is an extremely important pharmacophore skeleton that exists in many biologically active molecules.^5^ C(5) position of pyrimidine bases has been considered to be the most ideal site for DNA modification,^6^ since the introduced substituents were located in the major groove of the β-DNA duplex and basically did not wreck the DNA helical structure but strengthened the base pairing. Among the C(5)-modified pyrimidine nucleosides, 5-vinyl uracil derivatives exhibit considerable therapeutic and biological-imaging properties. Take the 5-vinyl uracil derivatives illustrated in Scheme 1a for example, *E*-5-(2-bromovinyl)-2′-deoxyuridine (BVDU) is an antiviral agent to herpes viruses;^7^ sparsomycin displays a rare broad-spectrum antibiotic and antitumor activity against bacteria, archaea, eucarya, and various cancer cell lines.^8^ The rapid inverse electron demand Diels–Alder reactions between the simplest 5-vinyl-2′-deoxyuridine (VdU) or 5-vinyluridine (VrU) and a fluorescent tetrazine^9^ could occur *in vitro* and in whole cells (Scheme 1b). Thus, these two minimalistic functionalized nucleosides have been employed as metabolic probe for labeling DNA and RNA after its incorporation into cellular nucleic acid.^10^

**Scheme 1:**
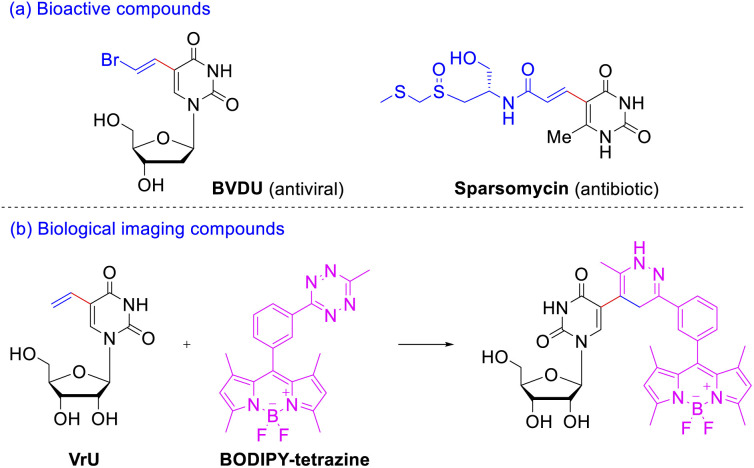
Application of 5-vinyluridine and analogues. (a) Representative 5-vinyl uracil derivatives as antiviral and antibiotic agents; (b) the inverse electron demand Diels–Alder reactions between VrU and tetrazine.

The preparation of 5-vinyl substituted uracil derivatives had gained the attentions from organic synthetic chemist as early as in the 1970s. The earliest synthetic strategy towards VdU or VrU employed palladium-catalyzed cross-coupling reactions between the 5-mercurated^11^ uridine and alkenes. Due to the high cost and toxicity of mercury reagents, the 5-mercurated uridine was subsequently replaced by 5-halo uracil nucleosides^12^ although prehalogenation of pyrimidine ring was still inevitable. A reversal Heck reaction of vinylic triflates with uridine was another route to 5-vinyl uridines.^13^ Itahara^14*a*^ and Hirota^14*b*^ (Scheme 2a) independently realized the oxidative coupling reaction of uracil nucleosides with alkenes in the 1980s under stoichiometric amounts of Pd(OAc)_2_, where Hirota reported two examples using catalytic Pd(OAc)_2_ with protected uracil and uridine substrates. Georg^15^ and Huang^16^ (Scheme 2b) successively implemented the catalytic C–H olefination of N-protected uracil in 2013 and 2017, respectively, but uridine and 2′-deoxyuridine (dU) carrying unprotected sugars were absent in the substrate scopes. Reasons for this blank might be the following: (1) coordination of the free hydroxy group or the nitrogen atom to palladium catalyst might hamper the carbometalation; (2) poor solubility of the nucleoside derivatives increases the difficulty in separation of the products in typical organic solvents. Nevertheless, in a continuation of our interest on Pd-catalyzed C–H activation,^17^ we aimed to develop an efficient Pd-catalyzed, direct C–H alkenylation^18^ of unprotected uridine, dU and uridine analogs under mild reaction conditions to fill up the gap beyond the precedent instances (Scheme 2c).

**Scheme 2:**
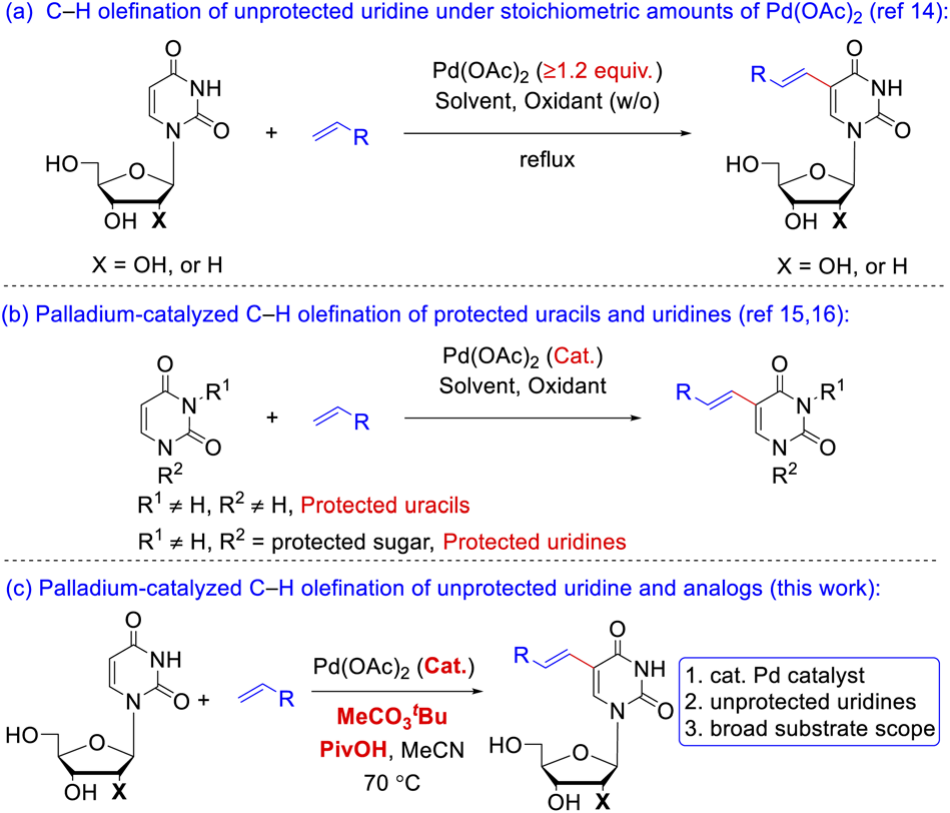
C–H olefination of uracil or uridine. (a) C–H olefination of unprotected uridine under stoichiometric amounts of Pd(OAc)_2_; (b) palladium-catalyzed C–H olefination of protected uracils; (c) palladium-catalyzed C–H olefination of unprotected uridine and analogs.

The investigation was initiated by choosing uridine 1a and methyl acrylate 2a as model substrate to extensively screen the alkenylation conditions. At first, we carried out the reaction by employing Pd(OAc)_2_ (10 mol%) as the catalyst and *t*-butyl perbenzoate (PhCO_3_^*t*^Bu) (2.0 equiv.) as the oxidant under ambient air in acetonitrile at 70 °C (Table 1, entry 1). The expected product 3aa was formed in 24% yield together with recovery of the starting material 1a (31%). Then a set of oxidants were inspected, including organic and inorganic oxidants (for more details, please see Table S1†). Among them, *t*-butyl peroxyacetate (CH_3_CO_3_^*t*^Bu) was the most effective one, promoting the reaction to form 3aa in 37% yield with concurrent recovery of 1a in 60% (Table 1, entry 2). The inorganic oxidants, either failed to yield any of the desired product (*e.g.* Cu(OAc)_2_) (Table 1, entry 4), or only led to produce 3aa in trace yield. Then, a series of solvents were screened (for more details, please see Table S2†), and satisfyingly, the use of acetic acid (Table 1, entry 10) significantly elevated the reaction yield to 59% with recovery of the 1a in 24%. Considering the effectiveness of the combination of Pd with HOAc,^19^ soon afterwards we conducted the reaction in acetonitrile with HOAc as the additive, which produced 3aa in 62% yield (Table 1, entry 11). We subsequently inspected an extensive array of additives with different acidity, finding that replacement of HOAc with pivalic acid (PivOH) furnished 82% yield of 3aa (Table 1, entry 12). Since Yu *et. al.* have demonstrated that the mono-N-protected amino acids (MPAAs)^20^ could effectively accelerate the Pd(ii)-catalyzed C–H olefination reaction, we further examined the influence of ligand on the coupling reaction. However, addition of ligands marginally decreased the formation of alkenylated product 3aa compared to the one without ligand (for more details, please see Table S4†). Therefore, the optimal reaction conditions were considered as below (Table 1, entry 12): Pd(OAc)_2_ (10 mol%) as the catalyst, CH_3_CO_3_^*t*^Bu (2.0 equiv.) as the oxidant, PivOH (2.0 equiv.) as the additive, under ambient air in acetonitrile at 70 °C. Notably, if the reaction was performed under inert atmosphere, like argon, the yield of 3aa dramatically decreased to 21% (Table 1, entry 13), whereas it was basically the same effective as under air if the reaction mixture was bubbled with O_2_ for 15 minutes before stirring and heating (Table 1, entry 14).

**Table 1:** Representative results for the optimization of the C–H olefination of uridine 1a^a^

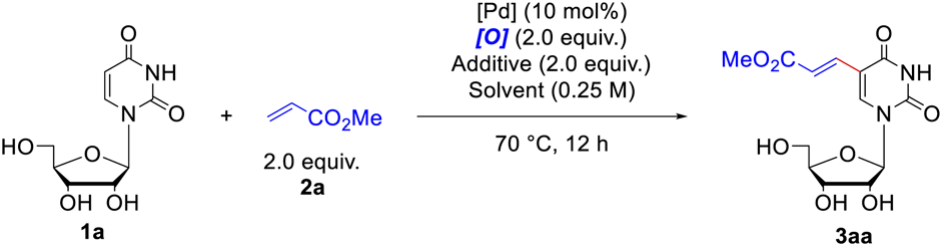						
Entry	Oxidant	Catalyst	Solvent	Additive	Yield^b^ (%)	Recovery^c^ (%)
1	PhCO_3_^*t*^Bu	Pd(OAc)_2_	CH_3_CN	—	24	31
2	MeCO_3_^*t*^Bu	Pd(OAc)_2_	CH_3_CN	—	37	60
3	(NH_4_)_2_S_2_O_8_	Pd(OAc)_2_	CH_3_CN	—	9	5
4	Cu(OAc)_2_	Pd(OAc)_2_	CH_3_CN	—	0	100
5	AgOAc	Pd(OAc)_2_	CH_3_CN	—	2	97
6	MeCO_3_^*t*^Bu	Pd(OAc)_2_	DMSO	—	8	92
7	MeCO_3_^*t*^Bu	Pd(OAc)_2_	DMA	—	18	77
8	MeCO_3_^*t*^Bu	Pd(OAc)_2_	HFIP	—	17	72
9	MeCO_3_^*t*^Bu	Pd(OAc)_2_	MeOH	—	0	81
10	MeCO_3_^*t*^Bu	Pd(OAc)_2_	HOAc	—	59	24
11	MeCO_3_^*t*^Bu	Pd(OAc)_2_	CH_3_CN	HOAc	62	26
12	MeCO_3_^*t*^Bu	Pd(OAc)_2_	CH_3_CN	PivOH	82	15
13^d^	MeCO_3_^*t*^Bu	Pd(OAc)_2_	CH_3_CN	PivOH	21	79
14^e^	MeCO_3_^*t*^Bu	Pd(OAc)_2_	CH_3_CN	PivOH	83	11

a Reaction conditions: uridine 1a (0.1 mmol), methyl acrylate 2a (2.0 equiv.), catalyst (10 mol%), oxidant (2.0 equiv.), additive (2.0 equiv.), solvent (0.4 mL) under air at 70 °C for 12 hours.

b Yields were determined by LC-MS.

c Rates of recovery were determined by LC-MS.

d The reaction was carried out under an argon atmosphere.

e The reaction was carried out under an oxygen atmosphere.

With the optimized reaction conditions in hand, the substrate scope of uracil-based nucleosides was firstly probed. As illustrated in Table 2, a range of uracil-based nucleosides were tolerated in the Pd-catalyzed dehydrogenative alkenylations. Both the naturally occurred uridine 1a and 2′-deoxy-uridine (dU, 1b) underwent smooth C5-alkenylation to afford the expected uridine or dU analogues in satisfactory yields (72% and 69%, respectively). The nucleoside analogues containing 2′-fluoro (1c) or 2′-methoxy group (1d) on the sugar ring worked well in this reaction, furnishing the corresponding 5-alkenylated products in good yields (80% and 87%, respectively). The hydroxy protected uridines, such as 2′,3′,5′-tri-*O*-acetyluridine (1e), 2′,3′-*O*-isopropylideneuridine (1f) and 3′, 5′-bis-*O*-diisopropyl-2′-deoxy-uridine (1g), were suitable substrates in the olefin modification reaction as well, converting to the 5-alkenylated uridine derivatives in 45–66% yields. Furthermore, the reactivity of a nucleoside analogue drug 1h, the anti-hepatitis C virus (HCV) agent, Sofosbuvir,^21^ was investigated, which could be successfully transformed to 3ha in 50% yield. Our method could be applicable to uridine monophosphate (UMP, 3ia) as well when the reaction was performed in mixed solvents of acetonitrile and water (10 : 1, v/v). Although the yield for late-stage functionalization of UMP was just acceptable (24%), the simple operation (only one-step), which omitted a series of reactions of protections, deprotections and phosphorylation, indicated the potential application in the field of chemical biology. C5-alkenyl modifications of the uridine diphosphate glucose (1j), deoxycytidine (1k) and the novel anti-SARS-Cov-2 agent, molnupiravir (1l), were probed as well (for more details, please see Table S7†). Reaction of 1j resulted in the formation of the target product 3ja in 23% yield. The two latter substrates with different nucleobases were very inert under present reaction conditions, and only small amounts of products could be detected by Liquid Chromatography-Mass Spectrometry (LC-MS). We speculated that the C4 amine groups in the deoxycytidine or Molnupiravir coordinated with the Pd catalyst and thus hindered the processes of carbopalladation.

**Table 2:** Scope of uridine and analogues for the C–H olefination of uridine 1 with methyl acrylate 2a a

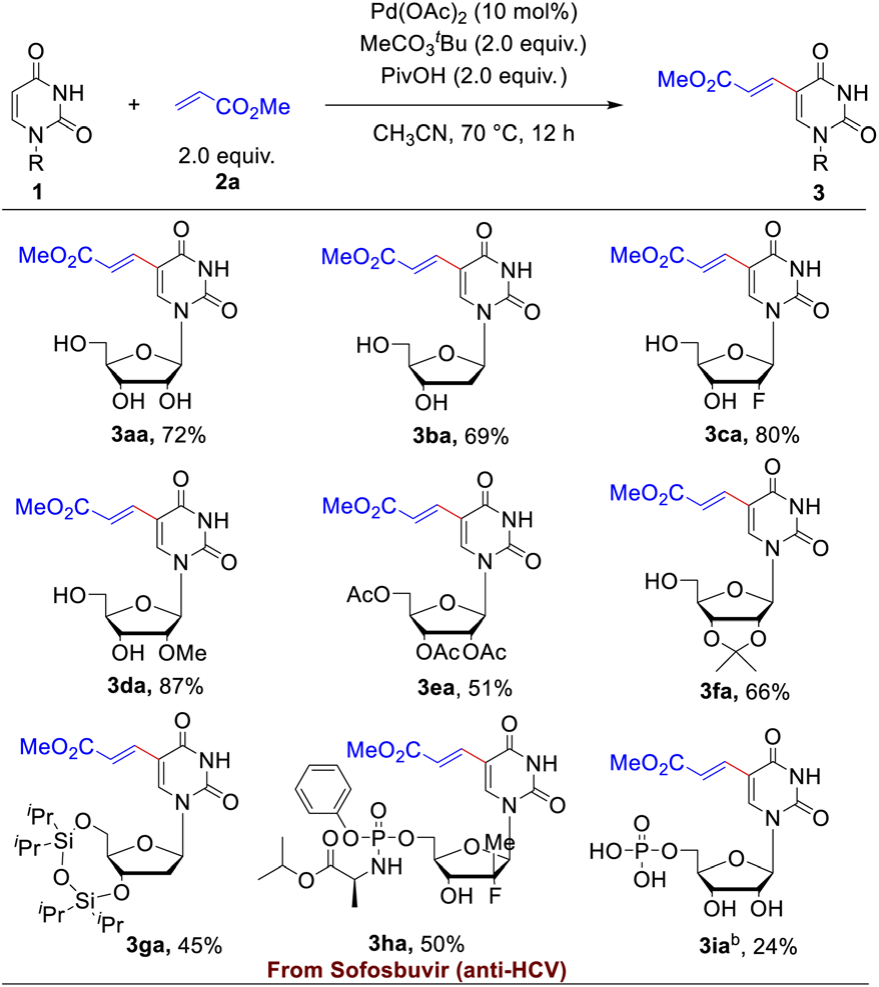

a Reaction conditions: uracil-based nucleosides/nucleotides 1 (0.1 mmol), methyl acrylate 2a (2.0 equiv.), Pd(OAc)_2_ (10 mol%), CH_3_CO_3_^*t*^Bu (2.0 equiv.), PivOH (2.0 equiv.), CH_3_CN (0.4 mL) under air at 70 °C for 12 hours.

b 1i (0.2 mmol), mixed solvents of CH_3_CN and H_2_O (10 : 1, v/v) was used. Isolated yield.

**Table 3:** Scope of alkenes for the C–H olefination of uridine 1a and 2′-deoxyuridine 1b^a^

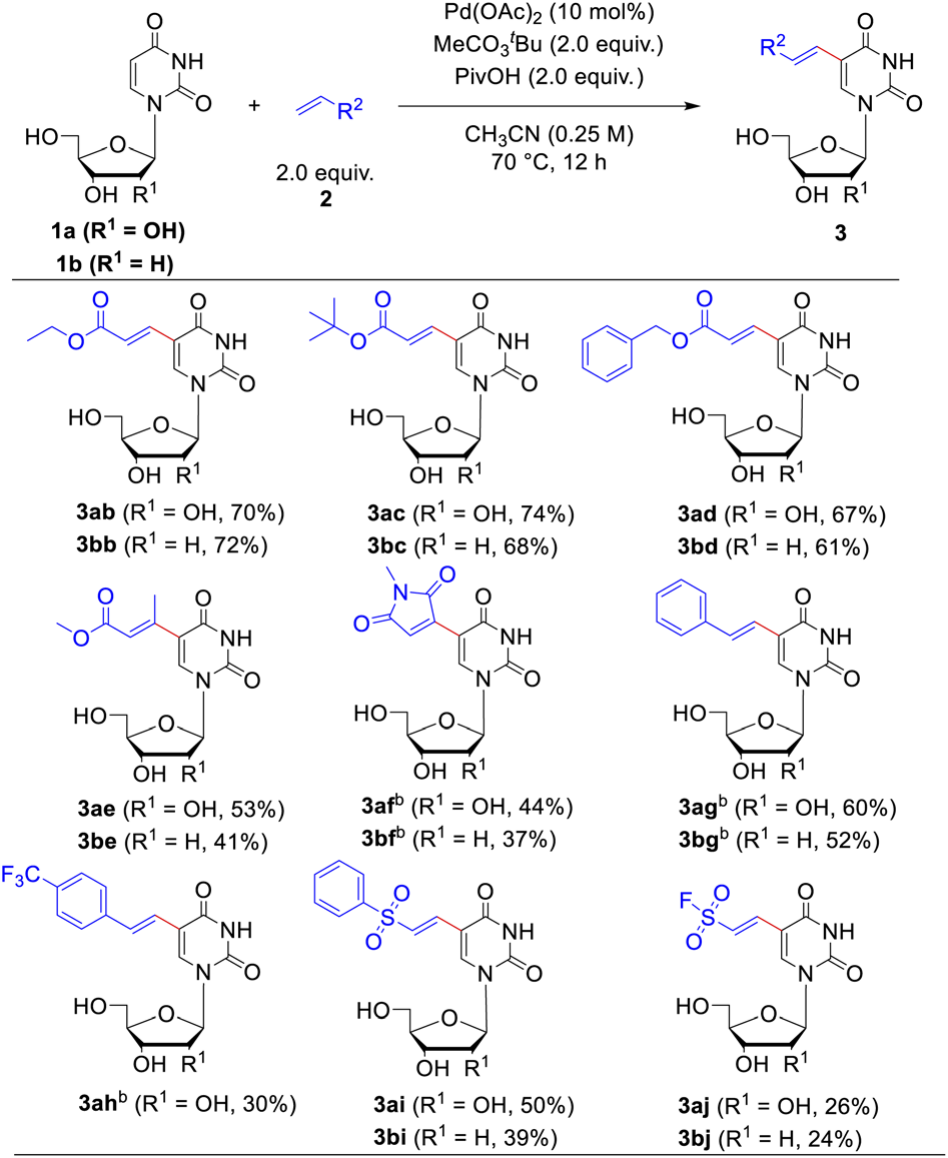

a Reaction conditions: uridine 1a or 2′-deoxyuridine 1b (0.1 mmol), olefines 2 (2.0 equiv.), Pd(OAc)_2_ (10 mol%), CH_3_CO_3_^*t*^Bu (2.0 equiv.), PivOH (2.0 equiv.), CH_3_CN (0.4 mL) under air at 70 °C for 12 hours.

b The reaction was carried out under O_2_ at 90 °C for 12 hours. Isolated yield.

Next, the applicability of this reaction to other olefin substrates was investigated (Table 3). A broad range of alkenes was found to undergo this C–H olefination reaction smoothly with uridine 1a or 2′-deoxyuridine (dU) 1b in acceptable to good yields. The size of ester group of the acrylate esters did not affect the reaction. Except methyl acrylate, ethyl- (2b), *tert*-butyl- (2c) and benzyl acrylate (2d) reacted efficiently with uridine or 2′-deoxyuridine to give the functionalized corresponding products in 61–74% yields. We were pleased to find that this catalytic system was not restricted to acrylate, maleimide, styrene and vinyl sulfone were competent coupling partners as well. However, disubstituted alkenes, such as methyl crotonate (2e) and *N*-methylmaleimide (2f), showed sluggish reactivity when reacted with uridine 1a or dU 1b compared to the monosubstituted alkenes (such as methyl acrylate 2a) probably due to the steric repulsion exerted by the other substituent. The reactions of *N*-methylmaleimide 2f and styrene 2g required a higher temperature (90 °C) and bubbling of O_2_ as the co-oxidant to ensure the yields. Introduction of substituent of electron-withdrawing group on the benzene ring of styrene, such as trifluoromethyl group (2h), decreased the reaction yield. The ethenesulfonyl fluoride 2j was also tolerated in the alkenylating modification, albeit the ethenesulfonyl fluoride showed some inactive reactivity compared to (vinylsulfonyl)benzene (2i) (26%, 24% yield for uridine and dU, respectively). Considering that the ethene sulfonyl fluoride is a newly emerged type of ligation functionality in click reaction^22^ to connect with small molecules, polymers, and biomolecules, this modification possesses potential application values in bioconjugate chemistry.

To evaluate the synthetic potential of this protocol (Scheme 3), gram-scale synthesis of 3aa was conducted. The uridine 1a (5.0 mmol) reacted with methyl acrylate 2a smoothly under 90 °C and O_2_ atmosphere, providing 3aa 7 in 62% yield (Scheme 3a). In addition, on-water reaction was also carried out to evaluate the potential application in chemical biology. When the reaction of uridine 1a with olefin 2a was performed in mixed solvents of acetonitrile and water (7 : 1, v/v), 48% yield of the expected product 3aa was obtained (Scheme 3b). With success in the achievement of preparation of the uridine alkenylsulfonyl fluorides, afterwards we turned to test the unique reactivity for corresponding transformation. As shown Scheme 3c, nucleophilic substitution at sulfur was achieved through the reaction of 3aj with *p*-methoxyphenol 4 to form sulfonate ester 5 in 56% yield.

**Scheme 3:**
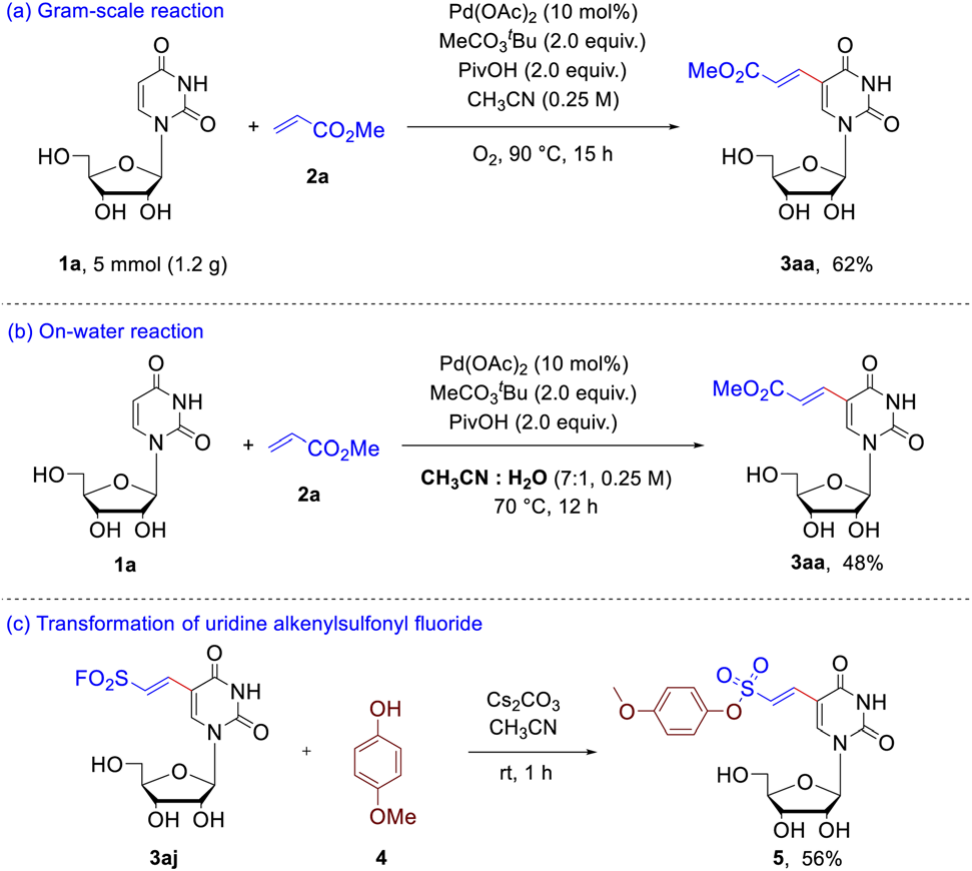
Application of this C–H olefination. (a) gram-scale experiment; (b) on-water reaction; (c) transformation of the uridine alkenylsulfonyl fluoride 3aj.

In summary, we developed an efficient and general catalytic synthetic method for direct C–H olefination of uridine/dU with unprotected hydroxy group and free amide nitrogen atom *via* oxidative Heck coupling reactions using CH_3_CO_3_^*t*^Bu as oxidant. Besides, a series of uridine analogues were compatible on the catalytical conditions, including 2′-F and 2′-OMe uridine, *O*-protected uridine/dU, an anti-HCV drug (Sofosbuvir) and the UMP. This protocol features atom economy, simple operation, shorter synthetic routes which precluded multistep sequences of protections and deprotections, environmentally friendly as prefunctionalization was avoided. The generality of this transformation provides a promisingly direct route to synthesize 5-alkenyl uridines/deoxyuridines and other analogues, which are of importance in medicinal chemistry. We also anticipate that this methodology will find applications in the field of chemical biology.

## Conflicts of interest

There are no conflicts to declare.

## Supplementary Material

RA-012-D2RA03681A-s001
