# Supplementary material for: Palladium-catalyzed C–H olefination of uridine, deoxyuridine, uridine monophosphate and uridine analogues
Source: RSC Adv. 2022 Sep 1;12(38):24930–4. doi: 10.1039/d2ra03681a (PMC9434382; doi:10.1039/d2ra03681a)
Supplement: RA-012-D2RA03681A-s001 [file RA-012-D2RA03681A-s001.pdf]

Electronic Supplementary Material (ESI) for RSC Advances.
This journal is © The Royal Society of Chemistry 2022

Supporting Information

Palladium-catalyzed C–H Olefination of Uridine, Deoxyuridine, Uridine Monophosphate and Uridine Analogues

Qin Zhao, Ruoqian Xie, Yuxiao Zeng, Wanlu Li, Guolan Xiao, Yangyan Li and Gang Chen

Table of Contents

1. General information 2
2. Details for the direct C–H olefinations 3
2.1 Optimization of C–H olefinations 3
Table S1. Oxidant screening 3
Table S2. Solvent screening 4
Table S3. Additive screening 5
Table S4. Ligand screening 6
Table S5. Atmosphere screening 7
Table S6. Temperature and Time screening 7
2.2. Substrate scope 8
Table S7. Substrate scope of the uridines 8
Table S8. Substrate scope of the alkenes 9
2.3 General procedure 9
3. Characterization data for compounds 3aa-3ia, 3ab-3aj, 3bb-3bj 10
3.1 Applications of the methodology 22
a) Gram scale preparation of 3aa 22
b) On-water reaction [a] 22
c) Derivative of 3aj [12, 13] 23
4. References 25
5. NMR Spectra 26

1. General information

All the chemicals were purchased commercially and used without further purification. General reagents were obtained from Adamas, Leyan, Innochem, Laajoo and Bidepharm. Anhydrous solvents were obtained from J&K. Analytical thin layer chromatography was performed on 0.25 mm silica gel 60-F254. Visualization was carried out with UV light and Vogel's permanganate. ^{1}H NMR spectra were recorded on Bruker-400 MHz and Bruker-500 MHz instruments. When the ^{1}H NMR solvent was DMSO-*d*-6, chemical shifts were quoted in parts per million (ppm) referenced to 2.50 ppm for solvent DMSO-*d*-6. When the ^{1}H NMR solvent was Methanol-*d*-4, chemical shifts were quoted in parts per million (ppm) referenced to 3.31 ppm for solvent Methanol-*d*-4. When the ^{1}H NMR solvent was D_2O, chemical shifts were quoted in parts per million (ppm) referenced to 4.79 ppm for solvent D_2O. The following abbreviations (or combinations thereof) were used to explain multiplicities: s = singlet, d = doublet, t = triplet, q = quartet, m = multiple, dd = double doublet, dt = double triplet. Coupling constants, *J*, were reported in Hertz unit (Hz). ^{13}C NMR spectra were recorded on Bruker-400 instrument (101 MHz) and Bruker-500 instrument (126 MHz), and were fully decoupled by broad band proton decoupling. When the ^{13}C NMR solvent was DMSO-*d*-6, chemical shifts were quoted in parts per million (ppm) referenced to 39.52 ppm for solvent DMSO-*d*-6. When the ^{13}C NMR solvent was Methanol-*d*-4, chemical shifts were quoted in parts per million (ppm) referenced to 49.00 ppm for solvent Methanol-*d*-4. Reverse-phase column chromatography was performed on SepaBean® machine T from Santai Technologies in Changzhou, China, using ODS 45-60 mm C18 Spherical silica. The high-resolution mass spectra (HRMS) were recorded on an Agilent Mass spectrometer using ESI-TOF (electrospray ionization-time of flight). Optical rotations were measured on an Anton Paar MCP100 automatic polarimeter using a 100 mm path-length cell at 589 nm. Melting points were measured with microscope WRX-4 (Shanghai Yice).

2. Details for the direct C–H olefinations

2.1 Optimization of C–H olefinations

Table S1. Oxidant screening

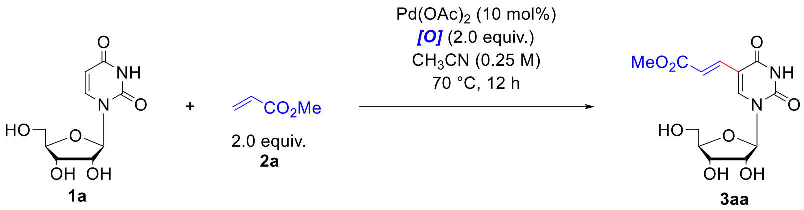

Entry[a]	[O]	Yield[b] of 3aa	Recovery[b] of 1a
**1**	$PhCO_3{}^tBu$	24%	31%
**2**	$MeCO_3{}^tBu$	37%	60%
**3**	DTBP	13%	85%
**4**	TBHP	20%	73%
**5**	$PhI(OAc)_2$	14%	43%
**6**	Oxone	9%	78%
**7**	DLP	1%	93%
**8**	Benzoquinone	1%	23%
**9**	*m*-CPBA	0%	23%
**10**	$(NH_4)_2S_2O_8$	9%	5%
**11**	H_2O_2	8%	91%
**12**	$Cu(OAc)_2$	0%	100%
**13**	CaOTf	0%	97%
**14**	AgOAc	2%	97%

[a] Conditions: uridine **1a** (0.1 mmol), methyl acrylate **2a** (0.2 mmol), $Pd(OAc)_2$ (0.01 mmol), oxidant (0.2 mmol), CH_3CN (0.4 mL) under air at 70 ℃ for 12 hours. [b] Yields and recovery were determined by LC-MS.

Table S2. Solvent screening

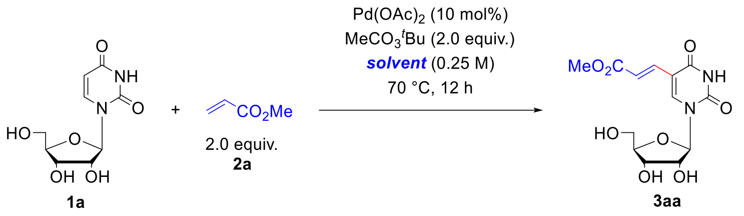

Entry[a]	Solvent	Yield[b] of 3aa	Recovery[b] of 1a
**1**	CH_3CN	37%	60%
**2**	Benzonitrile	2%	42%
**3**	H_2O	2%	98%
**4**	MeOH	< 1%	82%
**5**	CH_3CH_2OH	0%	83%
**6**	*t*-BuOH	6%	61%
**7**	*t*-AmlyOH	6%	69%
**8**	Glycol	0%	98%
**9**	Pyridine	0%	100%
**10**	DMSO	7%	91%
**11**	DMA	18%	77%
**12**	HFIP	17%	72%
**13**	CF_3CH_2OH	2%	76%
**14**	CH_3COOH	59%	24%
**15**	HCl	27%	0%
**16**	THF	2%	47%
**17**	Dioxane	16%	75%
**18**	Toluene	0%	44%
**19**	Cyclohexane	0%	100%
**20**	DCE	0%	99%

[a] Conditions: uridine **1a** (0.1 mmol), methyl acrylate **2a** (0.2 mmol), $Pd(OAc)_2$ (0.01 mmol), $MeCO_3{}^tBu$ (0.2 mmol), solvent (0.4 mL) under air at 70 ℃ for 12 hours. [b] Yields and recovery were determined by LC-MS.

Table S3. Additive screening

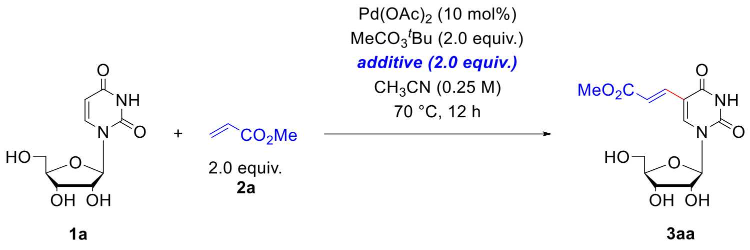

Entry[a]	Additive	Yield[b] of 3aa	Recovery[b] of 1a
**1**	AcOH	62%	26%
**2**	$CH_3CH_2C(CH_3)_2COOH$	76%	13%
**3**	$(CH_3)_2CHCOOH$	75%	22%
**4**	*p*-$CF_3(C_6H_4)CH_2COOH$	42%	35%
**5**	PivOH	82%	16%
**6**	TFA	2%	80%
**7**	HFIP	28%	69%
**8**	HCl	8%	60%
**9**	K_2CO_3	1%	99%
**10**	Li_2CO_3	0%	100%
**11**	Cs_2CO_3	0%	100%
**12**	KH_2PO_4	14%	83%
**13**	K_2HPO_4	13%	61%
**14**	$NaHCO_3$	1%	99%
**15**	NaH_2PO_4	17%	80%
**16**	LiF	0%	98%
**17**	CsF	0%	100%
**18**	$MgCl_2$	0%	100%

[a] Conditions: uridine **1a** (0.1 mmol), methyl acrylate **2a** (0.2 mmol), $Pd(OAc)_2$ (0.01 mmol), $MeCO_3{}^t\!Bu$ (0.2 mmol), additive (0.2 mmol), CH_3CN (0.4 mL) under air at 70 ℃ for 12 hours. [b] Yields and recovery were determined by LC-MS.

Table S4. Amino acid and pyridine ligand screening

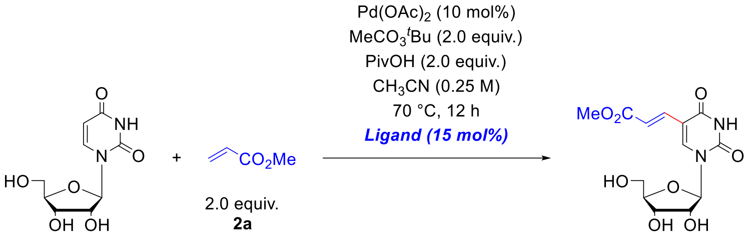

1a **3aa**

Entry[a]	ligand	Yield[b] of 3aa	Recovery[b] of 1a
**1**	-	79%	18%
**2**	Ac-Leu-OH	51%	10%
**3**	Ac-Gly-OH	67%	22%
**4**	Ac-Val-OH	78%	14%
**5**	Ac-Pro-OH	70%	24%
**6**	Ac-Phe-OH	60%	27%
**7**	Ac-Cys-OH	5%	93%
**8**	Z-Phe-OH	28%	72%
**9**	Z-Ala-OH	64%	25%
**10**	Z-Gly-OH	63%	27%
**11**	Boc-Gly-OH	64%	26%
**12**	Boc-Phe-OH	35%	61%
**13**	Boc-Leu-OH	74%	14%

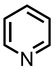

3aa/1a 0%/84%

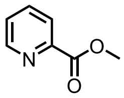

65%/35%

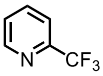

48%/18%

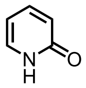

32%/68%

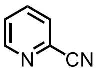

5%/81%

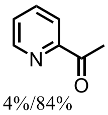

4%/84%

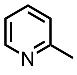

6%/21%

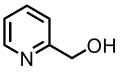

33%/20%

[a] Conditions: uridine **1a** (0.1 mmol), methyl acrylate **2a** (0.2 mmol), Pd(OAc)2 (0.01 mmol), MeCO3tBu (0.2 mmol), PivOH (0.2 mmol), CH3CN (0.4 mL), Ligand (0.015 mmol) under air at 70 ℃ for 12 hours.

Table S5. Atmosphere screening

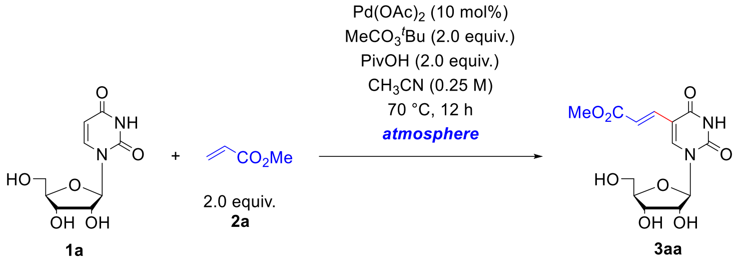

Entry[a]	Atmosphere	Yield[d] of 3aa	Recovery[d] of 1a
**1**	air	82%	15%
**2**[b]	Ar	21%	79%
**3**[c]	O_2	83%	11%

[a] Conditions: uridine **1a** (0.1 mmol), methyl acrylate **2a** (0.2 mmol), $Pd(OAc)_2$ (0.01 mmol), $MeCO_3{}^tBu$ (0.2 mmol), PivOH (0.2 mmol), CH_3CN (0.4 mL) under air at 70 ℃ for 12 hours. [b] The reaction was carried out under an argon atmosphere. [c] The reaction was carried out under an oxygen atmosphere. [d] Yields and recovery were determined by LC-MS.

Table S6. Temperature and Time screening

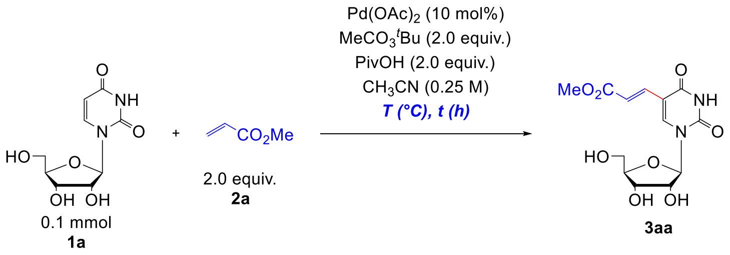

Entry[a]	T (℃)	t (h)	Yield[b] of 3aa	Recovery[b] of 1a
**1**	60	12	40	54
**2**	70	3	62	38
**3**	70	6	79	18
**4**	70	12	82	15
**5**	70	16	82	6
**6**	80	12	83	4
**7**	90	12	83	1

[a] Conditions: uridine **1a** (0.1 mmol), methyl acrylate **2a** (0.2 mmol), $Pd(OAc)_2$ (0.01 mmol), $MeCO_3{}^tBu$ (0.2 mmol), PivOH (0.2 mmol), CH_3CN (0.4 mL) under air. [b] Yields and recovery were determined by LC-MS.

2.2. Substrate scope

Table S7. Substrate scope of the uridines

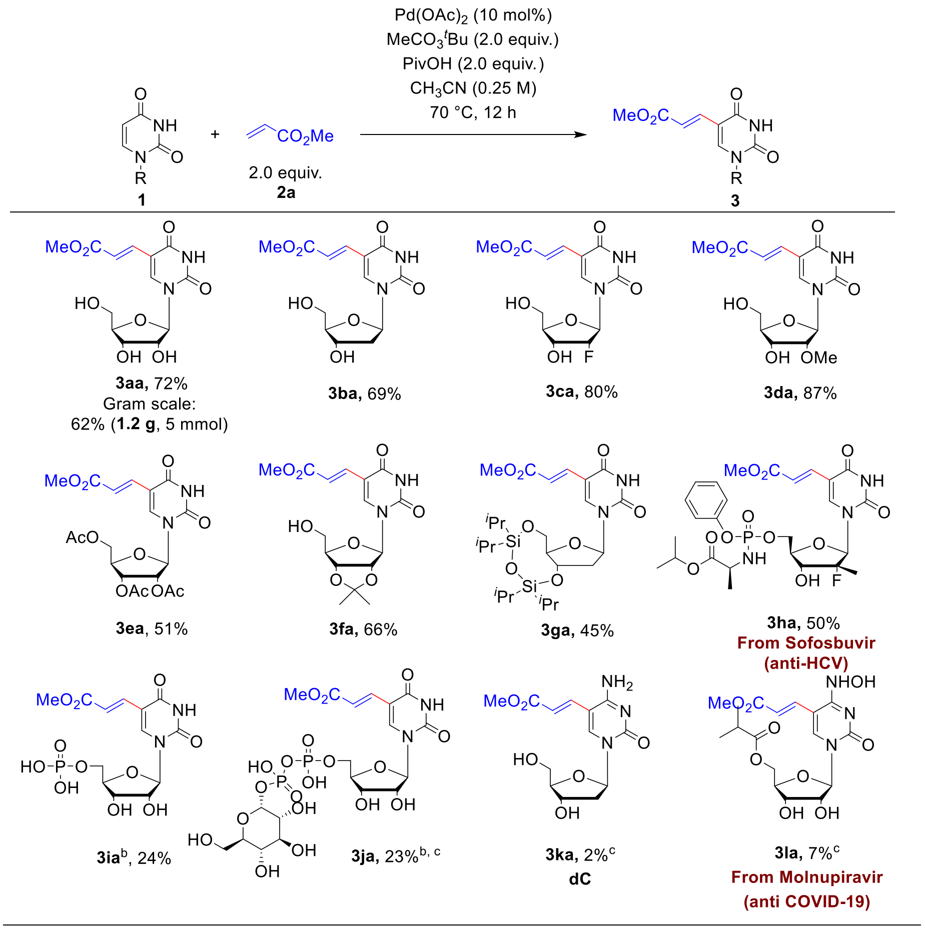

[a] Conditions: uracil-based nucleosides/nucleotides **1** (0.1 mmol), methyl acrylate **2a** (2.0 equiv.), Pd(OAc)$_2$ (10 mol%), $CH_3CO_3{}^t$Bu (2.0 equiv.), PivOH (2.0 equiv.), CH_3CN (0.4 mL) under air at 70 ℃ for 12 hours. [b] Mixed solvents of CH_3CN and H_2O (10:1, v/v) was used. [c] Yield determined by LC-MS and compound not isolated.

Table S8. Substrate scope of the alkenes

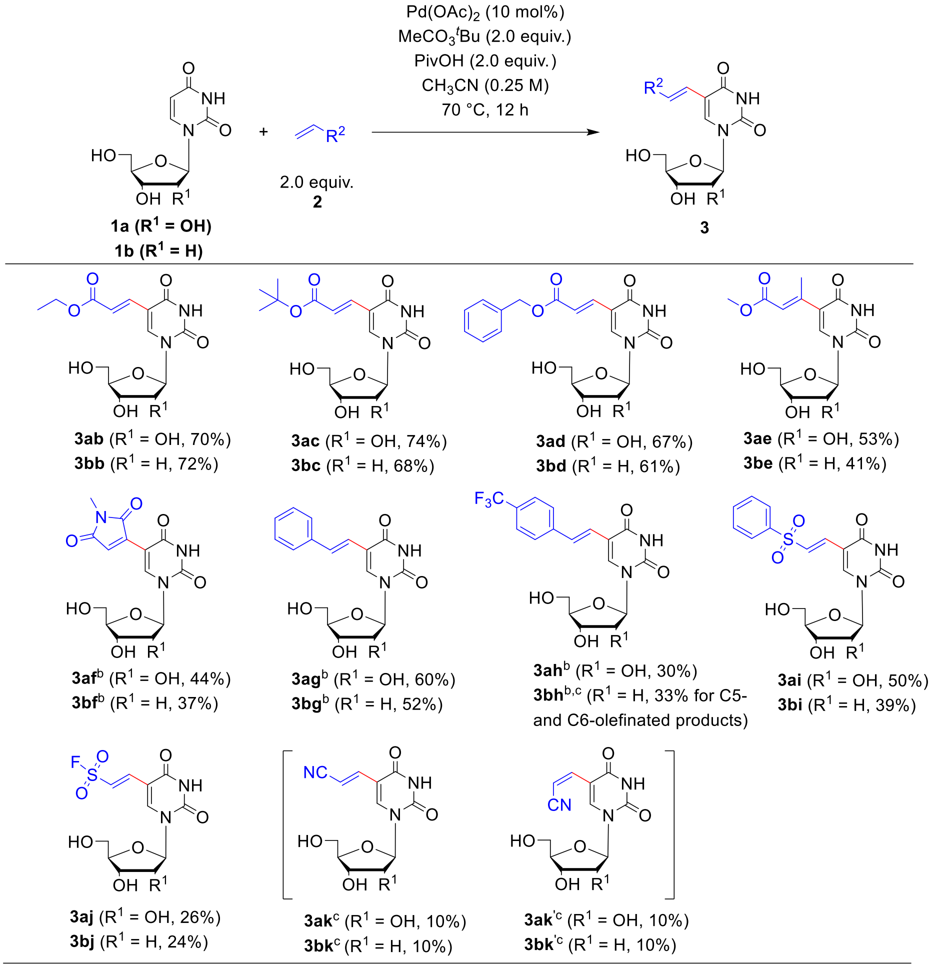

[a] Conditions: uridine **1a** or 2'-deoxyuridine **1b** (0.1 mmol), olefines **2** (2.0 equiv.), $Pd(OAc)_2$ (10 mol%), $CH_3CO_3{}^t Bu$ (2.0 equiv.), PivOH (2.0 equiv.), CH_3CN (0.4 mL) under air at 70 ℃ for 12 hours. [b] The reaction was carried out under O_2 at 90 ℃ for 12 hours. [c] Yield determined by LC-MS and compound not isolated. Isolated yield.

2.3 General procedure

General procedure A (0.1 mmol scale): A 10 mL reaction tube was charged with substrate **1a-1h** (0.1mmol, 1.0 equiv.), $Pd(OAc)_2$ (2.2 mg, 0.01 mmol, 10 mol%), $CH_3CO_3{}^t Bu$ (64 µL, 0.2 mmol, 2.0 equiv.) (50% solution in aromatic free mineral spirit), PivOH (20.4 mg, 0.2 mmol, 2.0 equiv.) and **2a-2j** (0.2 mmol, 2.0 equiv.), then 0.4 mL CH_3CN was added to dissolved the above mixture. The tube was sealed and the reaction mixture was then placed to a pre-heated oil bath to stir at 70 °C for 12 h. The reaction mixture was then cooled to room temperature. It was filtered through a pad of celite and washed with methanol. The filtrate was concentrated under reduced pressure and the residue was purified by PTLC (preparative TLC) (CH_2Cl_2:MeOH = 25:1 to 10:1) or reverse-phase column chromatography (C18 Spherical silica) (MeOH: H_2O = 0:1 to

1:1) to give the pure products **3aa-3ia, 3ab-3aj, 3bb-3bj**.

3. Characterization data for compounds 3aa-3ia, 3ab-3aj, 3bb-3bj

Methyl(*E*)-3-(1-((*2R,3R,4S,5R*)-3,4-dihydroxy-5-(hydroxy methyl) tetrahydrofuran-2-yl)-2,4-dioxo-1,2,3,4-tetrahydropyrimidin-5-yl)acrylate (3aa) [1]

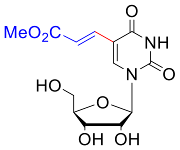

3aa was obtained following the general procedure **A** from **1a**. After purification by PTLC (preparative TLC) using CH_2Cl_2/MeOH (10/1) as the eluent, **3aa** was obtained as a yellow solid (23.6 mg, 72%), gram scale (5 mmol, 1.02 g, 62%). mp 180.7-182.6 °C; $[\alpha]_D^{25}$ -41.58 (c 0.670, MeOH); ^{1}H NMR (400 MHz, DMSO) δ 11.69 (s, 1H), 8.50 (s, 1H), 7.34 (d, *J* = 16.0 Hz, 1H), 6.84 (d, *J* = 15.6 Hz, 1H), 5.76 (d, *J* = 4.0 Hz, 1H), 5.46 (d, *J* = 4.0 Hz, 1H), 5.31 (t, *J* = 5.0 Hz, 1H), 5.09 (d, *J* = 3.6 Hz, 1H), 4.10–4.04 (m, 1H), 4.04–3.97 (m, 1H), 3.90–3.84 (m, 1H), 3.76–3.69 (m, 1H), 3.68 (s, 3H), 3.63–3.56 (m, 1H). ^{13}C NMR (126 MHz, DMSO) δ 167.2, 161.8, 149.5, 144.0, 138.0, 116.2, 108.2, 88.6, 84.6, 73.9, 69.0, 60.2, 51.3. HRMS-ESI m/z calcd for $C_{13}H_{16}N_2NaO_8$ $[M+Na]^+$ 351.0799; found 351.0798.

Methyl (*E*)-3-(1-((*2R,4S,5R*)-4-hydroxy-5-(hydroxymethyl)tetrahydrofuran-2-yl)-2,4-dioxo-1,2,3,4-tetrahydropyrimidin-5-yl)acrylate (3ba) [2]

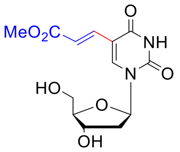

3ba was obtained following the general procedure **A** from **1b**. After purification by PTLC (preparative TLC) using CH_2Cl_2/MeOH (12/1) as the eluent, **3ba** was obtained as a yellow solid (19.6mg, 69%). mp 97.3-100.5 °C; $[\alpha]_D^{25}$ -1.86 (c 0.700, MeOH); ^{1}H NMR (400 MHz, DMSO) δ 11.63 (s, 1H), 8.42 (s, 1H), 7.37 (d, *J* = 15.6 Hz, 1H), 6.85 (d, *J* = 15.6 Hz, 1H), 6.13 (t, *J* = 6.4 Hz, 1H), 5.26 (d, *J* = 4.4 Hz, 1H), 5.17 (t, *J* = 5.2 Hz, 1H), 4.28–4.23 (m, 1H), 3.83–3.77 (m, 1H), 3.68 (s, 3H), 3.67–3.62 (m, 1H), 3.61–3.54 (m, 1H), 2.24–2.10 (m, 2H). ^{13}C NMR (101 MHz, MeOD) δ 169.7, 163.6, 151.1, 144.8, 138.8, 118.4, 110.4, 89.2, 87.1, 71.7, 62.4, 52.0, 41.9. HRMS-ESI m/z calcd for $C_{13}H_{16}N_2NaO_7$ $[M+Na]^+$ 335.0850; found 335.0852.

Methyl(*E*)-3-(1-((*2R,3R,4R,5R*)-3-fluoro-4-hydroxy-5-(hydroxymethyl) tetrahydrofuran-2-yl)-2,4-dioxo-1,2,3,4-tetrahydropyrimidin-5-yl)acrylate (3ca)

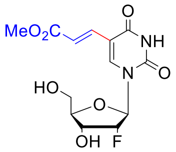

3ca was obtained following the general procedure **A** from **1c** on 0.1 mmol scale. After purification by PTLC (preparative TLC) using CH_2Cl_2/MeOH (15/1) as the eluent, **3ca** was obtained as a faint yellow solid (26.4 mg, 80%). mp 142.7-144.4 °C; $[\alpha]_D^{25}$ -58.49 (c 0.330, MeOH); ^{1}H NMR (400 MHz, DMSO) δ 11.71 (s, 1H), 8.53 (s, 1H), 7.31 (d, *J* = 16.0 Hz, 1H), 6.81 (d, *J* = 16.0 Hz, 1H), 5.89 (d, *J* = 16.8 Hz, 1H), 5.64 (d, *J* = 6.4 Hz, 1H), 5.49 (t, *J* = 4.4 Hz, 1H), 5.06 (dd, *J* = 52.8, 4.0 Hz, 1H), 4.26–4.11 (m, 1H), 3.95–3.81 (m, 2H), 3.70–3.60 (m, 4H). ^{19}F NMR (376 MHz, DMSO) δ -202.05. ^{13}C NMR (101 MHz, DMSO) δ 167.2, 161.9, 149.1, 143.5, 138.0, 116.2, 108.0, 93.8 (d, *J* = 184.3 Hz), 87.6 (d, *J* = 29.9 Hz), 83.1, 66.7 (d, *J* = 16.2 Hz), 58.7, 51.4. HRMS-ESI m/z calcd for $C_{13}H_{15}FN_2NaO_7$ $[M+Na]^+$ 353.0755; found 353.0756.

Methyl(*E*)-3-(1-((*2R,3R,4R,5R*)-4-hydroxy-5-(hydroxymethyl)-3-methoxytetrahydrofuran-2-yl)-2,4-dioxo-1,2,3,4-tetrahydropyrimidin-5-yl)acrylate (3da) [3]

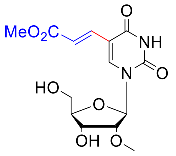

3da was obtained following the general procedure **A** from **1d** on 0.2 mmol scale. After purification by reverse-phase column chromatography (C18 Spherical silica) using MeOH/H_2O as the eluent, **3da** was obtained as a white solid (59.5 mg, 87%). mp 214.7-217.3 °C; $[\alpha]_D^{25}$ -13.524 (c 0.175, MeOH); ^{1}H NMR (400 MHz, DMSO) δ 11.69 (s, 1H), 8.55 (s, 1H), 7.32 (d, *J* = 16.0 Hz, 1H), 6.83 (d, *J* = 16.0 Hz, 1H), 5.83 (d, *J* = 3.6 Hz, 1H), 5.42 (brs, 1H), 5.20 (d, *J* = 5.6 Hz, 1H), 4.20–4.10 (m, 1H), 3.90–3.81 (m, 2H), 3.74 (d, *J* = 12.0 Hz, 1H), 3.67 (s, 3H), 3.61 (d, *J* = 12.0 Hz, 1H), 3.39 (s, 3H). ^{13}C NMR (101 MHz, DMSO) δ 167.2, 149.4, 143.7, 138.0, 116.2, 108.2, 86.8, 84.8, 83.0, 67.6, 59.7, 57.7, 51.3. HRMS-ESI m/z calcd for $C_{14}H_{18}N_2NaO_8$ $[M+Na]^+$ 365.0955; found 365.0956.

(*2R,3R,4R,5R*)-2-(acetoxymethyl)-5-(5-((*E*)-3-methoxy-3-oxoprop-1-en-1-yl)-2,4-dioxo-3,4-dihydropyrimidin-1(2H)-yl)tetrahydrofuran-3,4-diyl diacetate (3ea)

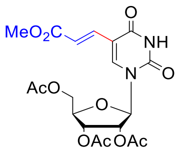

3ea was obtained following the general procedure **A** from **1e** on 0.2 mmol scale. After purification by PTLC (preparative TLC) using petroleum ether/ethyl acetate (2/3) as the eluent, **3ea** was obtained as a beige solid (46.5 mg, 51%). mp 104.2-106.6 °C; $[\alpha]_D^{25}$ -41.80 (c 0.500, MeOH); ^{1}H NMR (400 MHz, MeOD) δ 8.03 (s, 1H), 7.38 (d, *J* = 16.0 Hz, 1H), 6.94 (d, *J* = 16.0 Hz, 1H), 5.94 (d, *J* = 4.4 Hz, 1H), 5.56–5.49 (m, 1H), 5.43 (t, *J* = 5.8 Hz, 1H), 4.45–4.33 (m, 3H), 3.75 (s, 3H), 2.13–2.08 (m, 9H). ^{13}C NMR (101 MHz, MeOD) δ 172.2, 171.4, 171.3, 169.5, 163.3, 150.8, 145.1, 138.4, 119.4, 111.0, 91.0, 81.4, 74.5, 71.4, 64.0, 52.1, 20.8, 20.4, 20.3. HRMS-ESI m/z calcd for $C_{19}H_{22}N_2NaO_{11}$ $[M+Na]^+$ 477.1116; found 477.1113.

Methyl(*E*)-3-(1-((3a*R*,4*R*,6*R*,6a*R*)-6-(hydroxymethyl)-2,2-dimethyltetrahydrofuro[3,4-d][1,3]dioxol-4-yl)-2,4-dioxo-1,2,3,4-tetrahydropyrimidin-5-yl)acrylate (3fa)

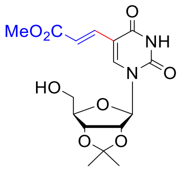

3fa was obtained following the general procedure **A** from **1f** on 0.1 mmol scale. After purification by PTLC (preparative TLC) using CH_2Cl_2/MeOH (22/1) as the eluent, **3fa** was obtained as a white solid (24.2 mg, 66%). mp 179.8-183.4 °C; $[\alpha]_D^{25}$ -40.36 (c 0.280, MeOH); ^{1}H NMR (400 MHz, DMSO) δ 11.74 (s, 1H), 8.35 (s, 1H), 7.34 (d, *J* = 16.0 Hz, 1H), 6.85 (d, *J* = 16.0 Hz, 1H), 5.85 (d, *J* = 2.4 Hz, 1H), 5.24 (t, *J* = 5.2 Hz, 1H), 4.95 (dd, *J* = 6.4, 2.4 Hz, 1H), 4.77 (dd, *J* = 6.4, 3.6 Hz, 1H), 4.15–4.11 (m, 1H), 3.68 (s, 3H), 3.67–3.61 (m, 1H), 3.60–3.53 (m, 1H), 1.49 (s, 3H), 1.29 (s, 3H). ^{13}C NMR (101 MHz, DMSO) δ 167.1, 161.8, 149.2, 145.0, 137.9, 116.4, 112.9, 108.2, 91.4, 87.1, 84.1, 80.2, 61.1, 51.3, 27.0, 25.2. HRMS-ESI m/z calcd for $C_{16}H_{20}N_2NaO_8$ $[M+Na]^+$ 391.1112; found 391.1114.

Methyl(*E*)-3-(2,4-dioxo-1-((6a*R*,8*R*,9a*S*)-2,2,4,4-tetraisopropyltetrahydro-6H-furo[3,2-f][1,3,5,2,4]trioxadisilocin-8-yl)-1,2,3,4-tetrahydropyrimidin-5-yl)acrylate (3ga)

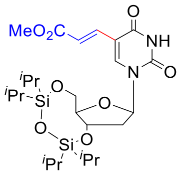

3ga was obtained following the general procedure **A** from **1g** on 0.1 mmol scale. After purification by PTLC (preparative TLC) using CH_2Cl_2/MeOH (25/1) as the eluent, **3ga** was obtained as a white solid (25.0 mg, 45%). mp 70.1-75.8 °C; $[\alpha]_D^{25}$ -60.36 (c 0.550, MeOH); ^{1}H NMR (400 MHz, DMSO) δ 11.70 (s, 1H), 8.06 (s, 1H), 7.31 (d, *J* = 16.0 Hz, 1H), 6.87 (d, *J* = 15.6 Hz, 1H), 6.00 (dd, *J* = 7.8, 3.4 Hz, 1H), 4.63–4.51 (m, 1H), 4.04 (dd, *J* = 12.2, 5.4 Hz, 1H), 3.95 (dd, *J* = 12.4, 3.2 Hz, 1H), 3.79–3.72 (m, 1H), 3.67 (s, 3H), 2.58–2.52 (m, 1H), 2.38–2.29 (m, 1H), 1.11–0.94 (m, 28H). ^{13}C NMR (101 MHz, DMSO) δ 167.1, 161.7, 149.0, 144.4, 138.0, 116.5, 108.1, 84.6, 84.4, 70.1, 61.8, 51.3, 17.4, 17.2(2C), 17.1, 16.9, 16.9, 16.8, 12.7, 12.5, 12.2, 11.9. HRMS-ESI m/z calcd for $C_{25}H_{42}N_2NaO_8Si_2$ $[M+Na]^+$ 577.2372; found 577.2375.

Methyl(*E*)-3-(1-((2*R*,3*R*,4*R*)-3-fluoro-4-hydroxy-5-((((*S*)-(((*S*)-1-isopropoxy-1-oxopropan-2-yl)amino)(phenoxy)phosphoryl)oxy)methyl)-3-methyltetrahydrofuran-2-yl)-2,4-dioxo-1,2,3,4-tetrahydropyrimidin-5-yl)acrylate (3ha)

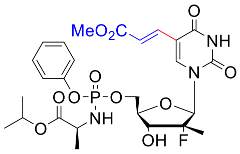

3ha was obtained following the general procedure **A** from **1h** on 0.1 mmol scale. After purification by PTLC (preparative TLC) using CH_2Cl_2/MeOH (15/1) as the eluent, **3ha** was obtained as a faint yellow solid (30.7 mg, 50%). mp 95.0-98.4 °C; $[\alpha]_D^{25}$ +8.79 (c 0.633, MeOH); ^{1}H NMR (400 MHz, MeOD) δ 7.95 (s, 1H), 7.44–7.31 (m, 3H), 7.27 (d, *J* = 8.0 Hz, 2H), 7.19 (t, *J* = 7.0 Hz, 1H), 6.98 (d, *J* = 16.0 Hz, 1H), 6.17 (d, *J* = 11.6 Hz, 1H), 4.96–4.89 (m, 2H), 4.59–4.40 (m, 2H), 4.18–4.09 (m, 1H), 4.00–3.94 (m, 1H), 3.66 (s, 3H), 1.42–1.31 (m, 6H), 1.19 (dd, *J* = 6.0, 2.0 Hz, 6H). ^{19}F NMR (376 MHz, MeOD) δ -161.9. ^{31}P NMR (162 MHz, MeOD) δ 4.0. ^{13}C NMR (101 MHz, MeOD) δ 174.4, 174.3, 169.6, 152.0, 152.0, 139.1, 130.8, 130.4, 126.3, 121.6, 121.5, 119.4, 116.2, 111.2, 102.4, 70.2, 52.0, 51.8, 25.3, 21.9, 21.9, 20.5 (d, *J* = 6.1 Hz), 17.0 (d, *J* = 25.7 Hz). HRMS-ESI m/z calcd for $C_{26}H_{33}FN_3NaO_{11}P$ $[M+Na]^+$ 636.1729; found 636.1725.

((2*R*,3*S*,4*R*,5*R*)-3,4-dihydroxy-5-(5-((*E*)-3-methoxy-3-oxoprop-1-en-1-yl)-2,4-

dioxo-3,4-dihydropyrimidin-1(2H)-yl)tetrahydrofuran-2-yl)methyl phosphate (3ia)

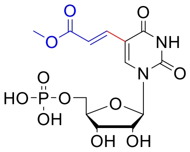

3ia was obtained following the general procedure **A** from **1i** on 0.2 mmol scale with mixed solvent of CH_3CN/H_2O (10/1, v/v). After purification by reverse-phase column chromatography (C18 Spherical silica) using $MeOH/H_2O$ as the eluent, **3ia** was obtained as a white solid (9.8 mg, 24%). mp 196.0-200.1 °C; $[\alpha]_D^{25}$ -51.652 (c 0.575, H_2O); ^1H NMR (400 MHz, D_2O) δ 8.22 (s, 1H), 7.48 (d, J = 15.6 Hz, 1H), 6.91 (d, J = 16.0 Hz, 1H), 5.98 (d, J = 4.8 Hz, 1H), 4.40 (t, J = 4.8 Hz, 1H), 4.33 (t, J = 4.6 Hz, 1H), 4.28 (s, 1H), 4.20–4.04 (m, 2H), 3.78 (s, 3H). ^{31}P NMR (162 MHz, D_2O) δ 0.7. ^{13}C NMR (101 MHz, D_2O) δ 170.1, 163.4, 150.6, 143.6, 137.8, 117.9, 109.9, 89.1, 83.3, 74.1, 69.5, 63.9, 52.1. HRMS-ESI m/z calcd for $C_{13}H_{17}N_2NaO_{11}P$ $[M+Na]^+$ 431.0462; found 431.0463.

Ethyl (*E*)-3-(1-((*2R,3R,4S,5R*)-3,4-dihydroxy-5-(hydroxymethyl)tetrahydrofuran-2-yl)-2,4-dioxo-1,2,3,4-tetrahydropyrimidin-5-yl)acrylate (3ab) [4]

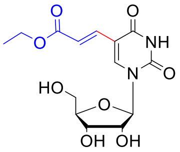

3ab was obtained following the general procedure **A** from **1a**. After purification by PTLC (preparative TLC) using CH_2Cl_2/MeOH (10/1) as the eluent, **3ab** was obtained as a white solid (24.0 mg, 70%). mp 198.2-200.0 °C; $[\alpha]_D^{25}$ -66.53 (c 0.473, MeOH); ^1H NMR (400 MHz, MeOD) δ 8.57 (s, 1H), 7.39 (d, J = 15.6 Hz, 1H), 6.88 (d, J = 15.6 Hz, 1H), 5.91 (d, J = 2.8 Hz, 1H), 4.23–4.16 (m, 4H), 4.08–4.02 (m, 1H), 3.93 (dd, J = 12.4, 2.4 Hz, 1H), 3.79 (dd, J = 12.4, 2.4 Hz, 1H), 1.29 (t, J = 7.0 Hz, 3H). ^{13}C NMR (101 MHz, MeOD) δ 169.2, 163.7, 151.3, 144.7, 138.4, 119.0, 110.4, 91.2, 86.2, 76.2, 70.6, 61.6, 61.5, 14.6. HRMS-ESI m/z calcd for $C_{14}H_{18}N_2NaO_8$ $[M+Na]^+$ 365.0955; found 365.0956.

Tert-butyl(*E*)-3-(1-((*2R,3R,4S,5R*)-3,4-dihydroxy-5-(hydroxymethyl) tetrahydrofuran-2-yl)-2,4-dioxo-1,2,3,4-tetrahydropyrimidin-5-yl)acrylate (3ac) [5]

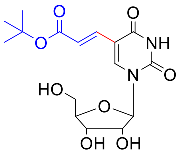

3ac was obtained following the general procedure **A** from **1a**. After purification by PTLC (preparative TLC) using CH_2Cl_2/MeOH (10/1) as the eluent, **3ac** was obtained as a white solid (27.4 mg, 74%). mp 165.3-167.3 °C; $[\alpha]_D^{25}$ -28.39 (c 0.830, MeOH); ^{1}H NMR (400 MHz, DMSO) δ 11.65 (s, 1H), 8.46 (s, 1H), 7.21 (d, *J* = 15.6 Hz, 1H), 6.74 (d, *J* = 16.0 Hz, 1H), 5.76 (d, *J* = 4.4 Hz, 1H), 5.46 (d, *J* = 5.2 Hz, 1H), 5.31 (t, *J* = 5.0 Hz, 1H), 5.10 (d, *J* = 5.6 Hz, 1H), 4.08 (dd, *J* = 9.2, 4.8 Hz, 1H), 4.01 (dd, *J* = 10.2, 5.0 Hz, 1H), 3.89–3.84 (m, 1H), 3.76–3.69 (m, 1H), 3.63–3.55 (m, 1H), 1.44 (s, 9H). ^{13}C NMR (101 MHz, DMSO) δ 166.1, 161.7, 149.5, 143.6, 136.8, 118.6, 108.3, 88.6, 84.7, 79.6, 73.8, 69.0, 60.2, 27.9(3C). HRMS-ESI m/z calcd for $C_{16}H_{22}N_2NaO_8$ $[M+Na]^+$ 393.1268; found 393.1270.

Benzyl(*E*)-3-(1-((*2R,3R,4S,5R*)-3,4-dihydroxy-5-(hydroxymethyl)tetrahydrofuran-2-yl)-2,4-dioxo-1,2,3,4-tetrahydropyrimidin-5-yl)acrylate (3ad) [5]

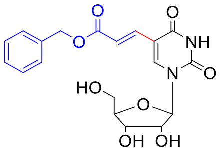

3ad was obtained following the general procedure **A** from **1a**. After purification by PTLC (preparative TLC) using CH_2Cl_2/MeOH (10/1) as the eluent, **3ad** was obtained as a white solid (27.0 mg, 67%). mp 179.6-183.7 °C; $[\alpha]_D^{25}$ -39.29 (c 0.330, MeOH); ^{1}H NMR (400 MHz, DMSO) δ 11.69 (s, 1H), 8.51 (s, 1H), 7.42–7.30 (m, 6H), 6.89 (d, *J* = 16.0 Hz, 1H), 5.76 (d, *J* = 4.4 Hz, 1H), 5.45 (d, *J* = 5.2 Hz, 1H), 5.30 (t, *J* = 5.2 Hz, 1H), 5.18 (s, 2H), 5.07 (d, *J* = 5.6 Hz, 1H), 4.08 (dd, *J* = 9.6, 4.8 Hz, 1H), 4.01 (dd, *J* = 10.4, 5.2 Hz, 1H), 3.89–3.84 (m, 1H), 3.77–3.68 (m, 1H), 3.59 (ddd, *J* = 12.2, 4.8, 3.2 Hz, 1H). ^{13}C NMR (101 MHz, DMSO) δ 166.5, 161.7, 149.4, 144.1, 138.3, 136.3, 128.4, 128.0, 127.9, 116.2, 108.1, 88.6, 84.6, 73.8, 68.9, 65.3, 60.1. HRMS-ESI m/z calcd for $C_{19}H_{20}N_2NaO_8$ $[M+Na]^+$ 427.1112; found 427.1118.

Methyl(*E*)-3-(1-((*2R,3R,4S,5R*)-3,4-dihydroxy-5-(hydroxy methyl)tetrahydrofuran-2-yl)-2,4-dioxo-1,2,3,4-tetrahydropyrimidin-5-yl)but-2-enoate (3ae)

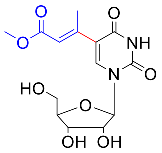

3ae was obtained following the general procedure **A** from **1a**. After purification by PTLC (preparative TLC) using CH_2Cl_2/MeOH (10/1) as the eluent, **3ae** was obtained as a white solid (18.1 mg, 53%). mp 163.9-168.6 °C; $[\alpha]_D^{25}$ -27.14 (c 0.280, MeOH); ^1H NMR (400 MHz, DMSO) δ 11.54 (s, 1H), 8.33 (s, 1H), 6.77 (s, 1H), 5.80 (d, *J* = 4.0 Hz, 1H), 5.46 (s, 1H), 5.30 (s, 1H), 5.13 (s, 1H), 4.14–4.06 (m, 1H), 4.02 (t, *J* = 4.4 Hz, 1H), 3.92–3.86 (m, 1H), 3.73–3.55 (m, 5H), 2.32 (s, 3H). ^{13}C NMR (101 MHz, DMSO) δ 166.9, 161.7, 149.6, 147.6, 140.6, 116.7, 113.2, 88.7, 84.7, 74.3, 69.5, 60.1, 50.9, 16.5. HRMS-ESI m/z calcd for $C_{14}H_{18}N_2NaO_8$ $[M+Na]^+$ 365.0955; found 365.0961.

1-((*2R,3R,4S,5R*)-3,4-dihydroxy-5-(hydroxymethyl)tetrahydrofuran-2-yl)-5-(1-methyl-2,5-dioxo-2,5-dihydro-1H-pyrrol-3-yl)pyrimidine-2,4(1H,3H)-dione (3af)[6]

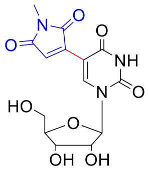

3af was obtained following the general procedure **A** from **1a** under O_2 at 90℃. After purification by PTLC (preparative TLC) using CH_2Cl_2/MeOH (10/1) as the eluent, **3af** was obtained as a yellow solid (15.5 mg, 44%). mp 273.9-276.5 °C; $[\alpha]_D^{25}$ -3.89 (c 0.300, MeOH); ^1H NMR (400 MHz, DMSO) δ 11.89 (s, 1H), 8.92 (s, 1H), 7.13 (s, 1H), 5.86 (d, *J* = 4.4 Hz, 1H), 5.50 (d, *J* = 5.2 Hz, 1H), 5.21 (d, *J* = 5.2 Hz, 1H), 4.92 (t, *J* = 5.2 Hz, 1H), 4.07 (dd, *J* = 9.4, 4.6 Hz, 1H), 3.94–3.90 (m, 1H), 3.69–3.59 (m, 2H), 2.90 (s, 3H). ^{13}C NMR (101 MHz, DMSO) δ 171.0, 170.9, 161.4, 149.2, 143.1, 135.7, 122.7, 103.6, 88.8, 85.2, 74.1, 70.3, 61.7, 23.6. HRMS-ESI m/z calcd for $C_{14}H_{15}N_3NaO_8$ $[M+Na]^+$ 376.0751; found 376.0748.

1-((*2R,3R,4S,5R*)-3,4-dihydroxy-5-(hydroxymethyl)tetrahydrofuran-2-yl)-5-((*E*)-styryl) pyrimidine-2,4(1H,3H)-dione (3ag) [7]

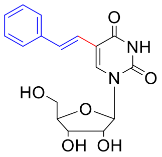

3ag was obtained following the general procedure **A** from **1a** under O_2 at 90℃. After purification by PTLC (preparative TLC) using CH_2Cl_2/MeOH (10/1) as the eluent, **3ag**

was obtained as a yellow solid (20.8 mg, 60%). mp 133.7-137.9 °C; $[\alpha]_D^{25}$ -50.11 (c 0.300, MeOH); ^{1}H NMR (400 MHz, DMSO) δ 11.51 (s, 1H), 8.32 (s, 1H), 7.46 (d, *J* = 7.6 Hz, 2H), 7.40–7.328 (m, 3H), 7.23 (t, *J* = 7.4 Hz, 1H), 6.88 (d, *J* = 16.4 Hz, 1H), 5.81 (d, *J* = 4.4 Hz, 1H), 5.46 (d, *J* = 4.4 Hz, 1H), 5.34 (t, *J* = 4.4 Hz, 1H), 5.12 (d, *J* = 4.0 Hz, 1H), 4.14–4.07 (m, 1H), 4.07–4.01 (m, 1H), 3.91–3.87 (m, 1H), 3.78–3.73 (m, 1H), 3.65–3.60 (m, 1H). ^{13}C NMR (101 MHz, DMSO) δ 162.3, 149.8, 138.1, 137.5, 128.8(2C), 127.8, 127.5, 126.1(2C), 120.9, 110.8, 88.4, 84.7, 74.0, 69.5, 60.5. HRMS-ESI m/z calcd for $C_{17}H_{18}N_2NaO_6$ $[M+Na]^+$ 369.1057; found 369.1061.

1-((*2R,3R,4S,5R*)-3,4-dihydroxy-5-(hydroxymethyl)tetrahydrofuran-2-yl)-5-((*E*)-4-(trifluoromethyl)styryl) pyrimidine-2,4(1H,3H)-dione (3ah) [8]

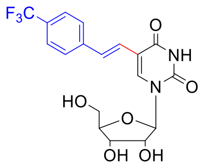

3ah was obtained following the general procedure **A** from **1a** under O_2 at 90℃.. After purification by PTLC (preparative TLC) using CH_2Cl_2/MeOH (10/1) as the eluent, **3ah** was obtained as a beige solid (12.4 mg, 30%). mp 202.5-207.3 °C; $[\alpha]_D^{25}$ -52.27 (c 0.383, MeOH); ^{1}H NMR (400 MHz, DMSO) δ 11.59 (s, 1H), 8.40 (s, 1H), 7.74–7.64 (m, 4H), 7.47 (d, *J* = 16.4 Hz, 1H), 7.04 (d, *J* = 16.4 Hz, 1H), 5.81 (d, *J* = 4.4 Hz, 1H), 5.48 (d, *J* = 5.2 Hz, 1H), 5.36 (t, *J* = 4.6 Hz, 1H), 5.12 (d, *J* = 5.2 Hz, 1H), 4.14–4.01 (m, 2H), 3.91–3.88 (m, 1H), 3.80–3.72 (m, 1H), 3.67–3.58 (m, 1H). ^{19}F NMR (376 MHz, DMSO) δ -60.8. ^{13}C NMR (101 MHz, DMSO) δ 162.1, 149.7, 141.6, 139.3, 127.4, 127.0, 126.5, 125.8, 125.6 (q, J_{C-F} = 3.8 Hz), 124.4 (m, J_{C-F} = 270.0 Hz), 124.1, 110.2, 88.4, 84.6, 73.9, 69.3, 60.3. HRMS-ESI m/z calcd for $C_{18}H_{17}F_3N_2NaO_6$ $[M+Na]^+$ 437.0931; found 437.0934.

1-((*2R,3R,4S,5R*)-3,4-dihydroxy-5-(hydroxymethyl)tetrahydrofuran-2-yl)-5-((*E*)-2-(phenylsulfonyl)vinyl)pyrimidine-2,4(1H,3H)-dione (3ai)

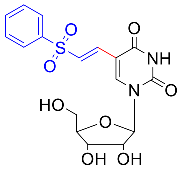

3ai was obtained following the general procedure **A** from **1a**. After purification by PTLC (preparative TLC) using CH_2Cl_2/MeOH (10/1) as the eluent, **3ai** was obtained as a white solid (20.5 mg, 50%). mp 234.0-235.8 °C; $[\alpha]_D^{25}$ -52.19 (c 0.350, MeOH); ^{1}H

NMR (400 MHz, MeOD) δ 8.64 (s, 1H), 7.89 (d, J = 7.2 Hz, 2H), 7.67 (t, J = 7.4 Hz, 1H), 7.63-7.56 (m, 2H), 7.52 (d, J = 15.2 Hz, 1H), 7.35 (d, J = 14.8 Hz, 1H), 5.89 (d, J = 2.8 Hz, 1H), 4.22–4.17 (m, 1H), 4.07-4.02 (m, 1H), 3.95 (dd, J = 12.4, 2.4 Hz, 1H), 3.80 (dd, J = 12.4, 2.4 Hz, 1H). ^{13}C NMR (126 MHz, DMSO) δ 161.7, 149.7, 145.9, 141.0, 135.9, 133.5, 129.7, 127.0, 125.5, 106.5, 88.8, 84.6, 73.8, 68.8, 60.1. HRMS-ESI m/z calcd for $C_{17}H_{18}N_2NaO_8S$ [M+Na]$^+$ 433.0676; found 433.0682.

(*E*)-2-(1-((*2R,3R,4S,5R*)-3,4-dihydroxy-5-(hydroxymethyl)tetrahydrofuran-2-yl)-2,4-dioxo-1,2,3,4-tetrahydropyrimidin-5-yl)ethene-1-sulfonyl fluoride (3aj)

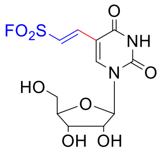

3aj was obtained following the general procedure **A** from **1a**. After purification by PTLC (preparative TLC) using CH_2Cl_2/MeOH (10/1) as the eluent, **3aj** was obtained as a yellow solid (9.2 mg, 26%). mp 162.5-166.3 °C; $[\alpha]_D^{25}$ -17.81 (c 0.640, MeOH); ^{1}H NMR (400 MHz, MeOD) δ 8.76 (s, 1H), 7.61 (dd, J = 15.0, 2.2 Hz, 1H), 7.51 (d, J = 14.8 Hz, 1H), 5.88 (d, J = 2.4 Hz, 1H), 4.22–4.17 (m, 2H), 4.07–4.03 (m, 1H), 3.95 (dd, J = 12.4, 2.4 Hz, 1H), 3.79 (dd, J = 12.4, 2.4 Hz, 1H). ^{19}F NMR (377 MHz, MeOD) δ 60.4 (s). ^{13}C NMR (101 MHz, MeOD) δ 163.1, 150.9, 148.9, 143.3, 117.8 (d, J = 27.4 Hz), 107.7, 91.6, 86.1, 76.3, 70.2, 61.3. HRMS-ESI m/z calcd for $C_{11}H_{13}FN_2NaO_8S$ [M+Na]$^+$ 375.0269; found 375.0265.

Ethyl (*E*)-3-(1-((*2R,4S,5R*)-4-hydroxy-5-(hydroxymethyl)tetrahydrofuran-2-yl)-2,4-dioxo-1,2,3,4-tetrahydropyrimidin-5-yl)acrylate (3bb) [9]

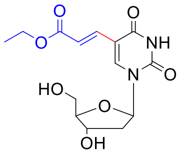

3bb was obtained following the general procedure **A** from **1b**. After purification by PTLC (preparative TLC) using CH_2Cl_2/MeOH (12/1) as the eluent, **3bb** was obtained as a white solid (23.4 mg, 72%). mp 167.5-169.4 °C; $[\alpha]_D^{25}$ +1.00 (c 0.400, MeOH); ^{1}H NMR (400 MHz, DMSO) δ 11.65 (s, 1H), 8.41 (s, 1H), 7.35 (d, J = 16.0 Hz, 1H), 6.84 (d, J = 16.0 Hz, 1H), 6.13 (t, J = 6.4 Hz, 1H), 5.27 (d, J = 4.4 Hz, 1H), 5.18 (t, J = 5.2 Hz, 1H), 4.29–4.22 (m, 1H), 4.14 (q, J = 7.1 Hz, 2H), 3.79 (dd, J = 6.8, 3.6 Hz, 1H), 3.69–3.53 (m, 2H), 2.24–2.11 (m, 2H), 1.22 (t, J = 7.0 Hz, 3H). ^{13}C NMR (101 MHz, MeOD) δ 169.3, 163.7, 151.1, 144.7, 138.6, 118.9, 110.4, 89.2, 87.1, 71.7, 62.4, 61.5,

41.9, 14.6. HRMS-ESI m/z calcd for $C_{14}H_{18}N_2NaO_7$ $[M+Na]^+$ 349.1006; found 349.1004.

Tert-butyl(*E*)-3-(1-((*2R,4S,5R*)-4-hydroxy-5-(hydroxymethyl)tetrahydrofuran-2-yl)-2,4-dioxo-1,2,3,4-tetrahydropyrimidin-5-yl)acrylate (3bc) [10]

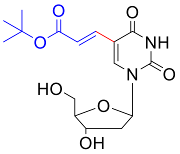

3bc was obtained following the general procedure **A** from **1b**. After purification by PTLC (preparative TLC) using CH_2Cl_2/MeOH (12/1) as the eluent, **3bc** was obtained as a white solid (24.2 mg, 68%). mp 105.8-107.4 °C; $[\alpha]_D^{25}$ +0.40 (c 0.420, MeOH); ^{1}H NMR (400 MHz, MeOD) δ 8.44 (s, 1H), 7.28 (d, *J* = 16.0 Hz, 1H), 6.78 (d, *J* = 16.0 Hz, 1H), 6.26 (t, *J* = 6.4 Hz, 1H), 4.45–4.40 (m, 1H), 3.95 (dd, *J* = 6.4, 3.2 Hz, 1H), 3.86 (dd, *J* = 12.0, 2.4 Hz, 1H), 3.76 (dd, *J* = 12.4, 3.2 Hz, 1H), 2.38–2.23 (m, 2H), 1.49 (s, 9H). ^{13}C NMR (101 MHz, MeOD) δ 168.7, 163.7, 151.1, 144.4, 137.5, 120.8, 110.5, 89.2, 87.0, 81.5, 71.7, 62.4, 41.9, 28.4. HRMS-ESI m/z calcd for $C_{16}H_{22}N_2NaO_7$ $[M+Na]^+$ 377.1319; found 377.1322.

Benzyl(*E*)-3-(1-((*2R,4S,5R*)-4-hydroxy-5-(hydroxymethyl)tetrahydrofuran-2-yl)-2,4-dioxo-1,2,3,4-tetrahydropyrimidin-5-yl)acrylate (3bd)

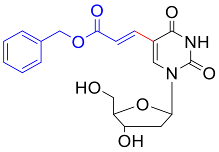

3bd was obtained following the general procedure **A** from **1b**. After purification by PTLC (preparative TLC) using CH_2Cl_2/MeOH (12/1) as the eluent, **3bd** was obtained as a white solid (23.7 mg, 61%). mp 89.6-90.3 °C; $[\alpha]_D^{25}$ +1.11 (c 0.330, MeOH); ^{1}H NMR (400 MHz, MeOD) δ 8.49 (s, 1H), 7.44 (d, *J* = 15.6 Hz, 1H), 7.40–7.29 (m, 4H), 6.94 (d, *J* = 16.0 Hz, 1H), 6.25 (t, *J* = 6.4 Hz, 1H), 5.20 (s, 2H), 4.42 (dt, *J* = 6.1, 4.0 Hz, 1H), 3.95 (q, *J* = 3.3 Hz, 1H), 3.85 (dd, *J* = 12.2, 3.0 Hz, 1H), 3.75 (dd, *J* = 12.2, 3.4 Hz, 1H), 2.38–2.22 (m, 2H). ^{13}C NMR (101 MHz, MeOD) δ 169.0, 163.7, 151.1, 144.9, 139.0, 137.7, 129.5(2C), 129.2(2C), 118.5, 110.4, 89.2, 87.1, 71.7, 67.2, 62.4, 41.9. HRMS-ESI m/z calcd for $C_{19}H_{20}N_2NaO_7$ $[M+Na]^+$ 411.1163; found 411.1171.

Methyl (*E*)-3-(1-((*2R,4S,5R*)-4-hydroxy-5-(hydroxymethyl)tetrahydrofuran-2-yl)-2,4-dioxo-1,2,3,4-tetrahydropyrimidin-5-yl)but-2-enoate (3be)

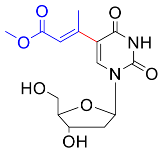

3be was obtained following the general procedure **A** from **1b**. After purification by PTLC (preparative TLC) using CH_2Cl_2/MeOH (12/1) as the eluent, **3be** was obtained as a white solid (13.4 mg, 41%). mp 175.4-176.7 °C; $[\alpha]_D^{25}$ +6.67 (c 0.270, MeOH); ^{1}H NMR (400 MHz, MeOD) δ 8.35 (s, 1H), 6.66 (s, 1H), 6.31 (t, *J* = 6.6 Hz, 1H), 4.46–4.40 (m, 1H), 3.96 (q, *J* = 2.9 Hz, 1H), 3.83 (dd, *J* = 12.0, 2.8 Hz, 1H), 3.75 (dd, *J* = 12.0, 2.8 Hz, 1H), 3.69 (s, 3H), 2.40 (s, 3H), 2.34–2.25 (m, 2H). ^{13}C NMR (126 MHz, MeOD) δ 169.1, 163.7, 151.4, 149.3, 141.4, 118.9, 116.1, 89.2, 87.0, 72.1, 62.5, 51.5, 41.9, 17.4. HRMS-ESI m/z calcd for $C_{14}H_{18}N_2NaO_7$ $[M+Na]^+$ 349.1006; found 349.1009.

1-((*2R,4S,5R*)-4-hydroxy-5-(hydroxymethyl)tetrahydrofuran-2-yl)-5-(1-methyl-2,5-dioxo-2,5-dihydro-1H-pyrrol-3-yl)pyrimidine-2,4(1H,3H)-dione (3bf) [6]

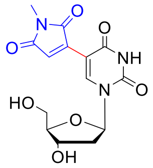

3bf was obtained following the general procedure **A** from **1b** under O_2 at 90℃. After purification by PTLC (preparative TLC) using CH_2Cl_2/MeOH (12/1) as the eluent, **3bf** was obtained as a yellow solid (12.5 mg, 37%). mp >300 °C; $[\alpha]_D^{25}$ -20.42 (c 0.360, DMSO); ^{1}H NMR (400 MHz, DMSO) δ 11.84 (s, 1H), 8.99 (s, 1H), 7.12 (s, 1H), 6.15 (t, *J* = 6.4 Hz, 1H), 5.37 (d, *J* = 4.0 Hz, 1H), 4.92 (t, *J* = 5.2 Hz, 1H), 4.27-4.21 (m, 1H), 3.91-3.85 (m, 1H), 3.65–3.51 (m, 2H), 2.89 (s, 3H), 2.24-2.21 (m, 1H), 2.17–2.07 (m, 1H). ^{13}C NMR (101 MHz, DMSO) δ 171.1, 171.0, 161.6, 149.0, 143.1, 135.9, 122.2, 103.2, 88.1, 85.7, 70.7, 61.7, 23.6. HRMS-ESI m/z calcd for $C_{14}H_{15}N_3NaO_7$ $[M+Na]^+$ 360.0802; found 360.0804.

1-((*2R,4S,5R*)-4-hydroxy-5-(hydroxymethyl)tetrahydrofuran-2-yl)-5-((*E*)-styryl)pyrimidine-2,4(1H,3H)-dione (3bg) [11]

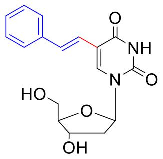

3bg was obtained following the general procedure **A** from **1b** under O_2 at 90℃. After

purification by PTLC (preparative TLC) using CH_2Cl_2/MeOH (12/1) as the eluent, **3bg** was obtained as a faint yellow solid (17.2 mg, 52%). mp 99.8-101.4 °C; $[\alpha]_D^{25}$ -3.49 (c 0.430, MeOH); ^{1}H NMR (400 MHz, MeOD) δ 8.32 (s, 1H), 7.45 (d, *J* = 8.0 Hz, 2H), 7.41 (d, *J* = 16.4 Hz, 1H), 7.30 (t, *J* = 7.4 Hz, 2H), 7.20 (t, *J* = 7.2 Hz, 1H), 6.89 (d, *J* = 16.4 Hz, 1H), 6.32 (t, *J* = 6.6 Hz, 1H), 4.46 (dd, *J* = 9.2, 4.0 Hz, 1H), 3.96 (dd, *J* = 6.2, 3.0 Hz, 1H), 3.88 (dd, *J* = 12.2, 2.6 Hz, 1H), 3.79 (dd, *J* = 12.0, 3.2 Hz, 1H), 2.38–2.25 (m, 2H). ^{13}C NMR (101 MHz, MeOD) δ 164.5, 151.4, 139.1, 138.8, 130.3, 129.6(2C), 128.5, 127.3(2C), 121.0, 113.3, 89.0, 86.7, 71.9, 62.6, 41.7. HRMS-ESI m/z calcd for $C_{17}H_{18}N_2NaO_5$ $[M+Na]^+$ 353.1108; found 353.1112.

1-((*2R,4S,5R*)-4-hydroxy-5-(hydroxymethyl)tetrahydrofuran-2-yl)-5-((*E*)-2-(phenylsulfonyl)vinyl)pyrimidine-2,4(1H,3H)-dione (3bi)

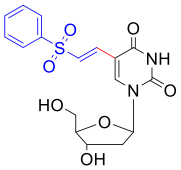

3bi was obtained following the general procedure **A** from **1b**. After purification by PTLC (preparative TLC) using CH_2Cl_2/MeOH (12/1) as the eluent, **3bi** was obtained as a yellow solid (15.4 mg, 39%). mp >300 °C; $[\alpha]_D^{25}$ +0.71 (c 0.380, MeOH); ^{1}H NMR (400 MHz, DMSO) δ 11.73 (s, 1H), 8.48 (s, 1H), 7.91–7.82 (m, 2H), 7.75–7.68 (m, 1H), 7.68–7.61 (m, 2H), 7.46 (d, *J* = 15.2 Hz, 1H), 7.39 (d, *J* = 14.8 Hz, 1H), 6.10 (t, *J* = 6.4 Hz, 1H), 5.29 (d, *J* = 4.4 Hz, 1H), 5.19 (t, *J* = 5.2 Hz, 1H), 4.29–4.21 (m, 1H), 3.80 (q, *J* = 3.7 Hz, 1H), 3.71–3.52 (m, 2H), 2.21–2.13 (m, 2H). ^{13}C NMR (101 MHz, MeOD) δ 163.4, 150.9, 146.9, 142.6, 136.9, 134.5, 130.5(2C), 128.4(2C), 127.8, 108.6, 89.2, 87.2, 71.5, 62.3, 41.9. HRMS-ESI m/z calcd for $C_{17}H_{18}N_2NaO_7S$ $[M+Na]^+$ 417.0727; found 417.0727.

(*E*)-2-(1-((*2R,4S,5R*)-4-hydroxy-5-(hydroxymethyl)tetrahydrofuran-2-yl)-2,4-dioxo-1,2,3,4-tetrahydropyrimidin-5-yl)ethene-1-sulfonyl fluoride (3bj)

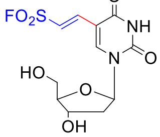

3bj was obtained following the general procedure **A** from **1b**. After purification by PTLC (preparative TLC) using CH_2Cl_2/MeOH (12/1) as the eluent, **3bj** was obtained as a white solid (8.1 mg, 24%). mp 193.3-194.2 °C; $[\alpha]_D^{25}$ +5.68 (c 0.370, MeOH); ^{1}H

NMR (400 MHz, MeOD) δ 8.66 (s, 1H), 7.62 (dd, J = 14.8, 2.4 Hz, 1H), 7.55 (dd, J = 15.2, 0.8 Hz, 1H), 6.22 (t, J = 6.2 Hz, 1H), 4.41 (dt, J = 6.4, 4.0 Hz, 1H), 3.96 (dd, J = 6.8, 3.2 Hz, 1H), 3.87 (dd, J = 12.0, 2.8 Hz, 1H), 3.76 (dd, J = 12.2, 3.4 Hz, 1H), 2.42-2.34 (m, 1H), 2.30–2.20 (m, 1H). ^{19}F NMR (377 MHz, MeOD) δ 60.4 (s). ^{13}C NMR (101 MHz, MeOD) δ 163.1, 150.7, 149.0, 143.4, 117.7 (d, J = 27.6 Hz), 107.6, 89.3, 87.6, 71.4, 62.2, 42.1. HRMS-ESI m/z calcd for $C_{11}H_{13}FN_2NaO_7S$ $[M+Na]^+$ 359.0320; found 359.0315.

3.1 Applications of the methodology

a) Gram scale preparation of 3aa

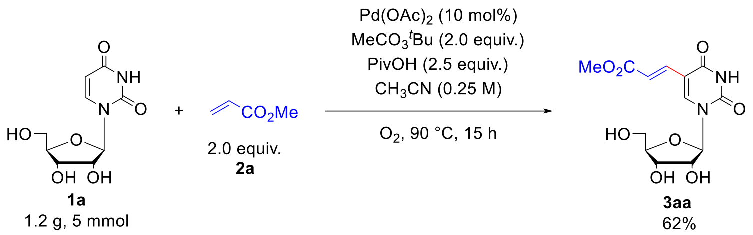

General procedure B (gram scale): A 250 mL reaction tube was charged with substrate **1a** (1.2 g, 5 mmol, 1.0 equiv.), $Pd(OAc)_2$ (0.5 mmol, 10 mol%), $CH_3CO_3{}^tBu$ (10 mmol, 2.0 equiv.) (50% solution in aromatic free mineral spirit), PivOH (12.5 mmol, 2.5 equiv.) and **2a** (10 mmol, 2.0 equiv.), then 20 mL CH_3CN were added to dissolved the mixture. The reaction solution was bubbled with O_2 for 30 min. The tube was sealed with a Teflon-lined cap and the reaction mixture was then placed to a pre-heated oil bath to stir at 90 °C for 15 h (*Caution: The tube was carefully capped and covered with safety shield.*). The reaction mixture was then cooled to room temperature. It was filtered through a pad of celite, and then washed with methanol. The filtrate was concentrated under reduced pressure and the residue was purified by column chromatography on silica gel using CH_2Cl_2/MeOH (50/1 to 25/1) as the eluent to give the pure product **3aa**.

b) On-water reaction [a]

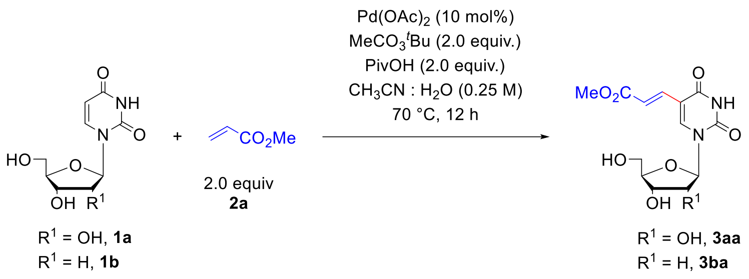

Entry	CH_3CN:H_2O	Yield[b] of 3		Recovery[b] of 1	
		3aa	3ba	1a	1b
**1**	10:1	61%	61%	32%	23%
**2**	7:1	48%[c]	52%	48%	35%
**3**	5:1	39%	38%	60%	55%
**4**	3:1	23%	26%	58%	65%
**5**	1:1	5%	9%	95%	90%
**6**	1:3	2%	2%	98%	98%
**7**	H_2O	2%	2%	98%	94%
**8**	CH_3CN	79%	73%	13%	8%

[a] Conditions: uridine **1a** or **1b** (0.1 mmol), methyl acrylate **2a** (0.2 mmol), $Pd(OAc)_2$ (0.01 mmol), $MeCO_3{}^tBu$ (0.2 mmol), PivOH (0.2 mmol), CH_3CN : H_2O (v/v, 0.4 mL) under air at 70 ℃ for 12 hours. [b] Yields and recovery were determined by LC-MS. [c] Isolated yield.

General procedure C (0.1 mmol scale): A 10 mL reaction tube was charged with substrate **1a** (0.1mmol, 1.0 equiv.), $Pd(OAc)_2$ (2.2 mg, 0.01 mmol, 10 mol%), $CH_3CO_3{}^tBu$ (64 µL, 0.2 mmol, 2.0 equiv.) (50% solution in aromatic free mineral spirit), PivOH (20.4 mg, 0.2 mmol, 2.0 equiv.) and **2a** (18µL, 0.2 mmol, 2.0 equiv.), then 0.35 mL CH_3CN and 0.05 mL H_2O were added to dissolve the above mixture. The tube was sealed and the reaction mixture was then placed to a pre-heated oil bath to stir at 70 °C for 12 h. The reaction mixture was then cooled to room temperature. It was filtered through a pad of celite and washed with methanol. The filtrate was concentrated under reduced pressure and the residue was purified by PTLC (preparative TLC) (CH_2Cl_2:MeOH = 10:1) to give the pure product **3aa**.

c) Derivative of 3aj [12, 13]

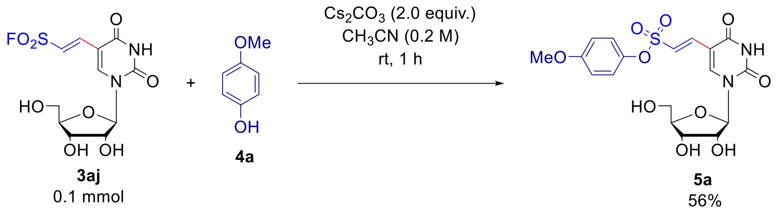

A 10 mL sample vial was charged with **3aj** (35 mg, 0.10 mmol, 1.0 equiv.), *p*-methoxyphenol (13.6 mg, 0.11 mmol, 1.1 equiv.) and Cs_2CO_3 (65.2 mg, 0.20 mmol, 2.0 equiv.), and then CH_3CN (0.5 mL) was added to dissolve the above mixture. The reaction was stirred at ambient temperature for 1 h. Then it was filtered through a pad of celite and washed with methanol. The filtrate was concentrated under reduced pressure and the residue was purified by PTLC (preparative TLC) (CH_2Cl_2:MeOH = 10:1) to give the pure product **5a** as a white solid (26.8 mg , 56% yield).

4-methoxyphenyl (*E*)-2-(1-((2*R*,3*R*,4*S*,5*R*)-3,4-dihydroxy-5-(hydroxymethyl) tetrahydrofuran-2-yl)-2,4-dioxo-1,2,3,4-tetrahydropyrimidin-5-yl) ethene-1-sulfonate (5a)

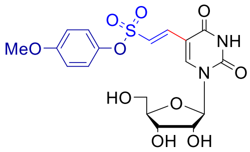

After purification by PTLC (preparative TLC) using CH_2Cl_2/MeOH (10/1) as the eluent, **5a** was obtained as a white solid (26.8 mg, 56%). mp 180.5-184.6 °C; $[\alpha]_D^{25}$ -17.36 (c 0.457, MeOH); ^{1}H NMR (400 MHz, DMSO) δ 11.84 (s, 1H), 8.54 (s, 1H), 7.46 (d, *J* = 15.2 Hz, 1H), 7.26 – 7.12 (m, 3H), 6.97 (d, *J* = 8.8 Hz, 2H), 5.73 (d, *J* = 4.0 Hz, 1H), 5.49 (d, *J* = 5.2 Hz, 1H), 5.27 (t, *J* = 5.2 Hz, 1H), 5.11 (d, *J* = 5.6 Hz, 1H), 4.06 (dd, *J* = 9.2, 4.4 Hz, 1H), 3.98 (dd, *J* = 10.4, 5.2 Hz, 1H), 3.89–3.82 (m, 1H), 3.75 (s, 3H), 3.72–3.65 (m, 1H), 3.61–3.53 (m, 1H). ^{13}C NMR (101 MHz, DMSO) δ 161.6, 157.9, 149.3, 146.6, 142.5, 139.5, 123.5, 118.4, 114.9, 106.1, 88.9, 84.6, 73.8, 68.8, 60.1, 55.5. HRMS-ESI m/z calcd for $C_{18}H_{20}N_2NaO_{10}S$ $[M+Na]^+$ 479.0731; found 479.0740.

4. References

[1] D. Y. Piao, A. Basavapathruni, P. Iyidogan, G. X. Dai, W. Hinz, A. S. Ray, E. Murakami, J. Y. Feng, F. You, G. E. Dutschman, D. J. Austin, K. A. Parker and K. S. Anderson, *Bioorg. Med. Chem. Lett.,* 2013, **23**, 1511-1518.

[2] S. Bhilare, V. Gayakhe, A. V. Ardhapure, Y. S. Sanghvi, C. Schulzke, Y. Borozdina and A. R. Kapdi, *RSC. Adv.,* 2016, **6**, 83820-83830.

[3] Y. L. Ding, J. L. Girardet, Z. Hong, S. Z. Shaw and N. H. Yao, *Heterocycle.,* 2006, **68**, 521-530.

[4] R. Kumar, L. H. Xu, E. E. Knaus, L. I. Wiebe, D. R. Tovell, D. L. Tyrrell and T. M. Allen, *J. Med. Chem*., 1990, **33**, 717-723.

[5] R. F. Whale, P. L. Coe and R. T. Walker, *Nucleosides & Nucleotides.*, 1991, **10**, 1615-1624.

[6] T. Itahara, *Chem. Lett.*, 1986, **15**, 239-242.

[7] S. Izuta and M. Saneyoshi, *Chem. Pharm. Bull.,* 1987, **12**, 4829-4838.

[8] M. Segal and B. Fischer, *Org. Biomol. Chem.,* 2012, **10**, 1571-1580.

[9] W. E. Herve and C. Len, *RSC Adv.,* 2014, **4**, 46926-49929.

[10] V. Aucagne, S. B. Raboin, P. Guenot and L. A. Agrofoglio, *J. Comb. Chem.,* 2004, **6**, 717-723.

[11] V. Gayakhe, A. Ardhapure, A. R. Kapdi, Y. S. Sanghvi, J. L. Serrano, L. García, J. Peréz, J. García, G. Sánchez, C. Fischer and C. Schulzke, *J. Org. Chem.,* 2016, **81**, 2713-2729.

[12] T. S. B. Lou, S. W. Bagley and M. C. Willis, *Angew. Chem. Int. Ed*., 2019, **58**, 18859-18863.

[13] X. Y. Chen, Y. C. Wu, J. Zhou, P. Wang and J. Q. Yu, *Org. Lett.*, 2019, **21**, 1426-1429.

5. NMR Spectra

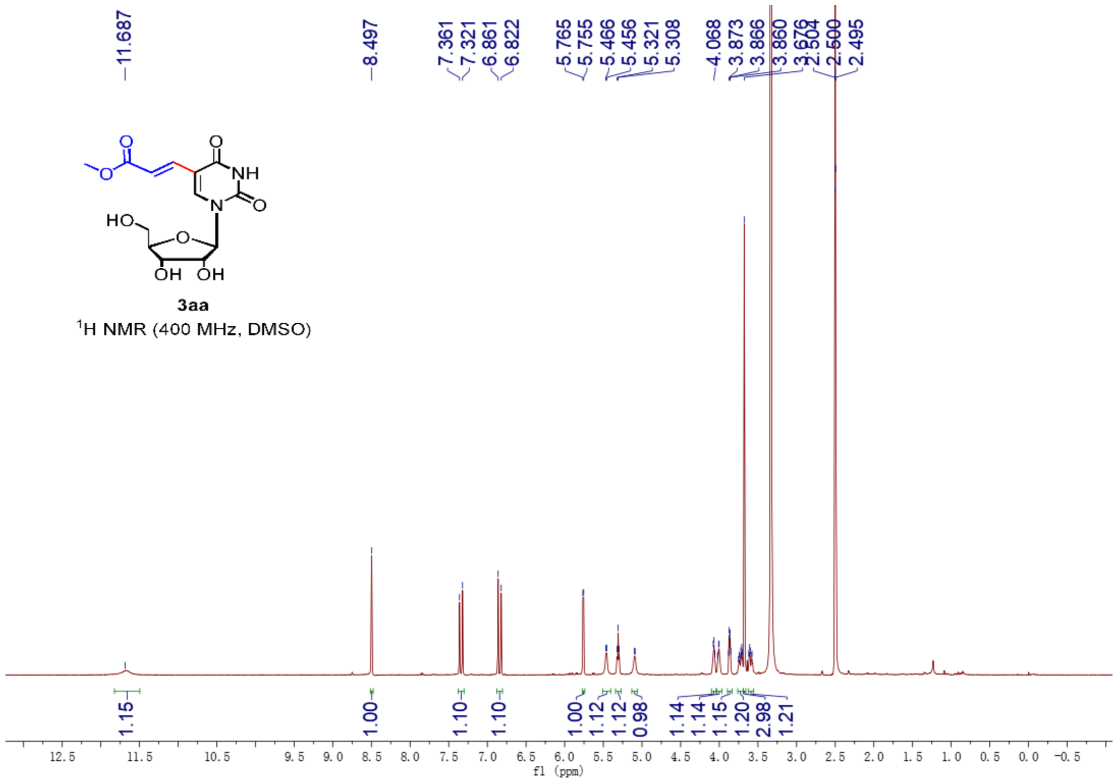

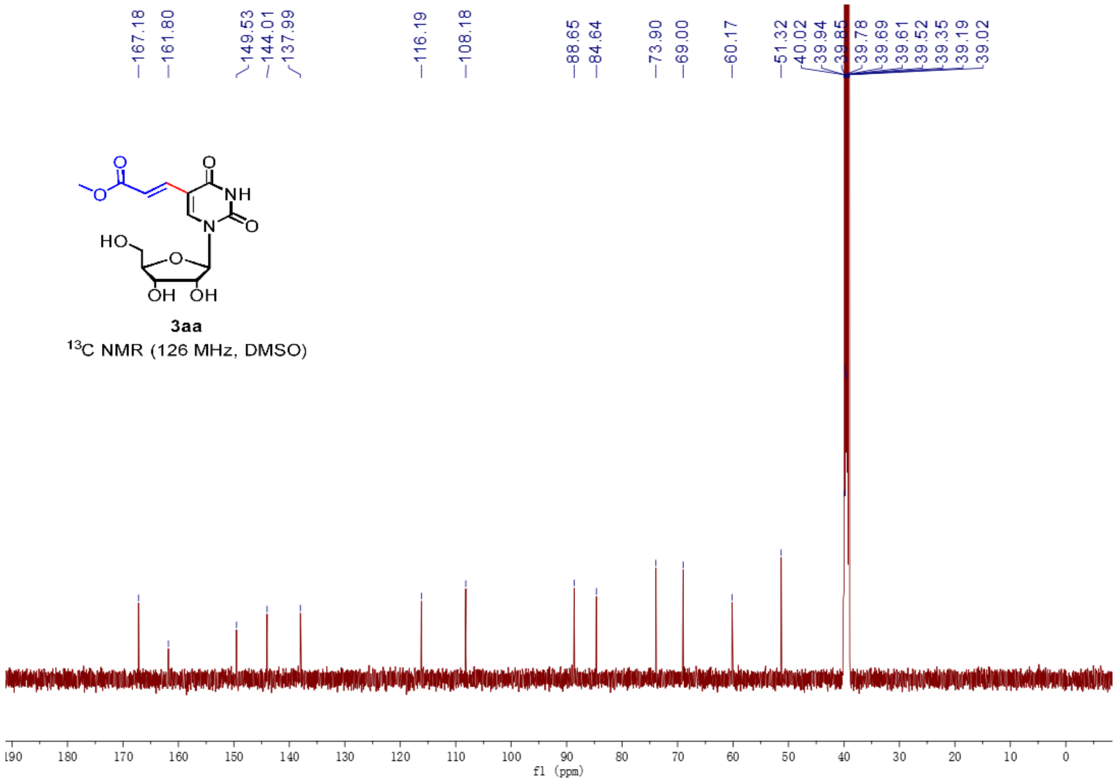

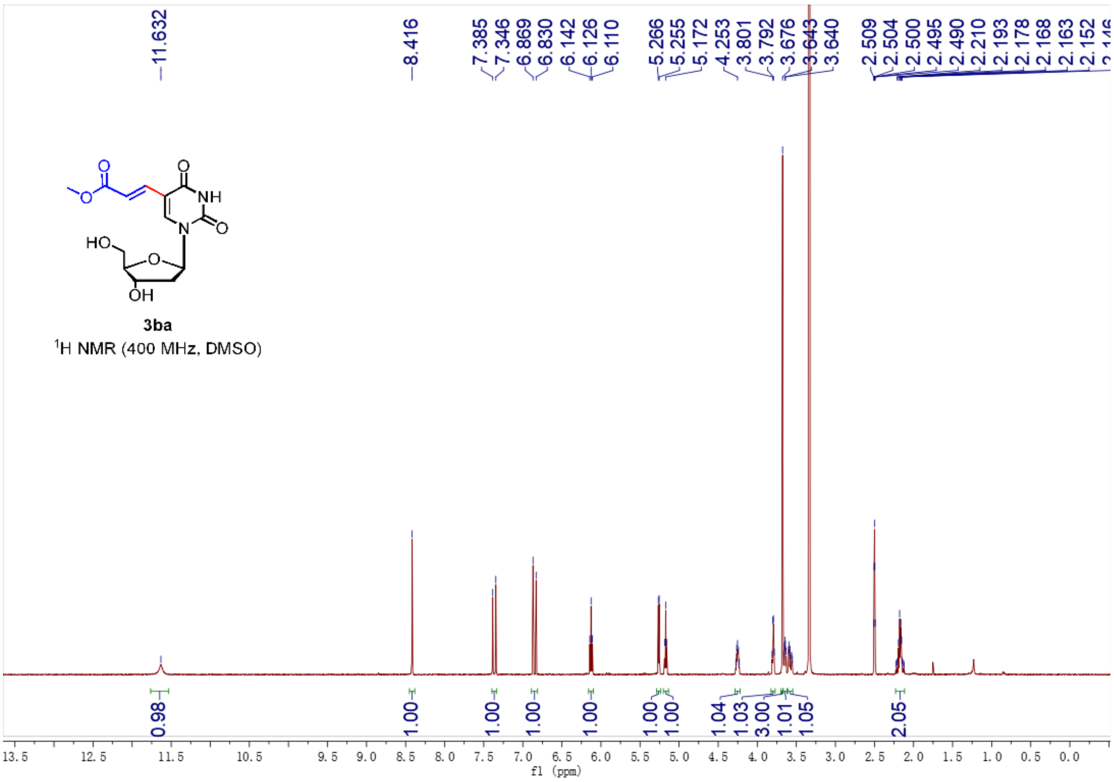
11.632
8.416
7.385
7.346
6.869
6.830
6.142
6.126
6.110
5.266
5.255
5.172
4.253
3.801
3.792
3.676
3.643
3.640
2.509
2.504
2.500
2.495
2.490
2.210
2.193
2.178
2.168
2.163
2.152
3ba
1H NMR (400 MHz, DMSO)
0.98
1.00
1.00
1.00
1.00
1.00
1.00
1.04
1.03
3.00
1.01
1.05
2.05
f1 (ppm)

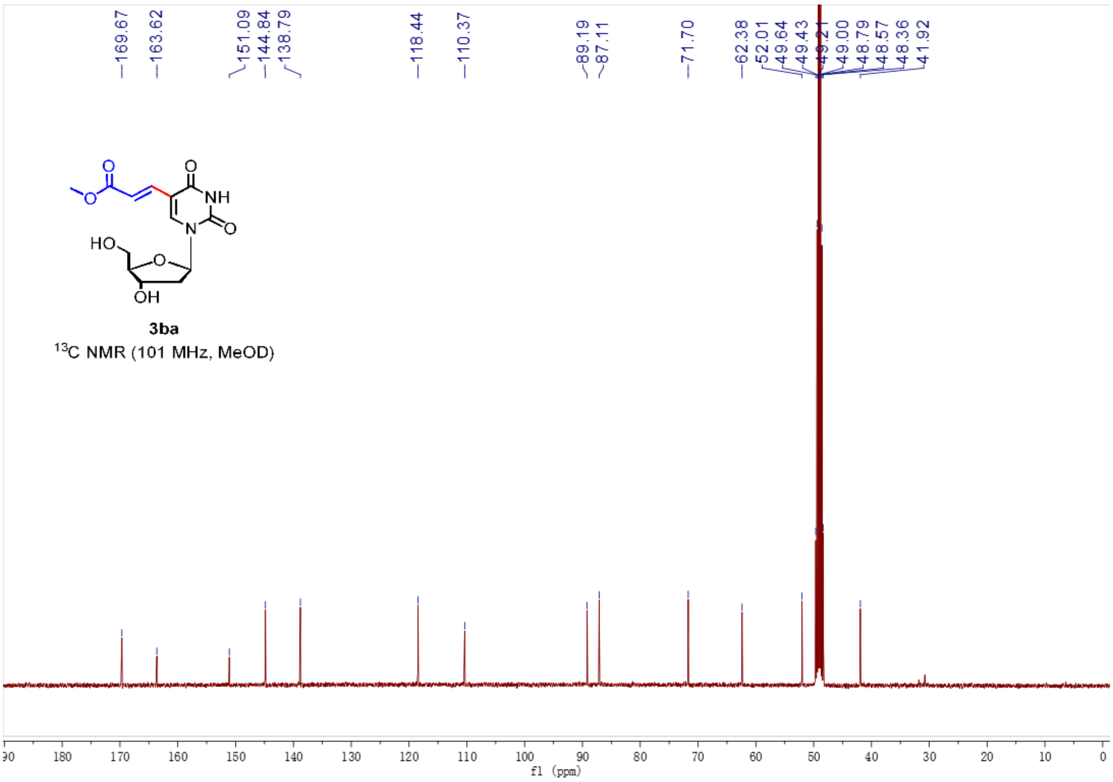
169.67
163.62
151.09
144.84
138.79
118.44
110.37
89.19
87.11
71.70
62.38
52.01
49.64
49.43
49.21
49.00
48.79
48.57
48.36
41.92
3ba
13C NMR (101 MHz, MeOD)
f1 (ppm)

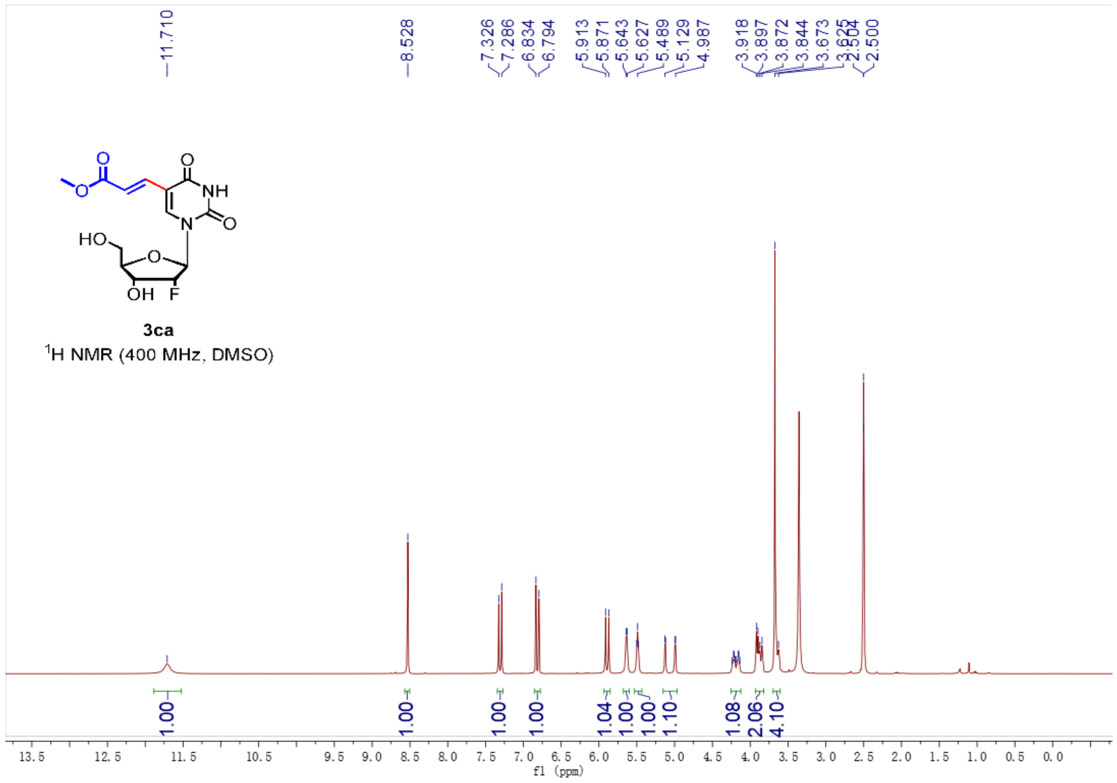
3ca
1H NMR (400 MHz, DMSO)
f1 (ppm)

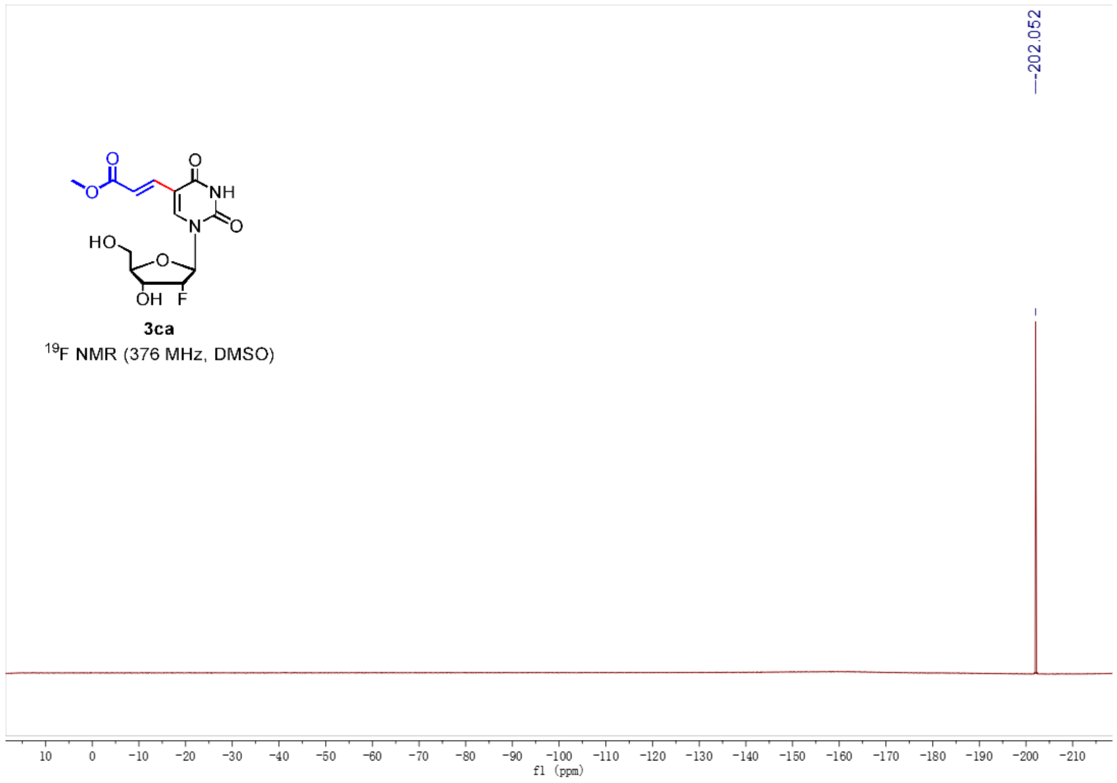
-202.052
3ca
19F NMR (376 MHz, DMSO)
f1 (ppm)

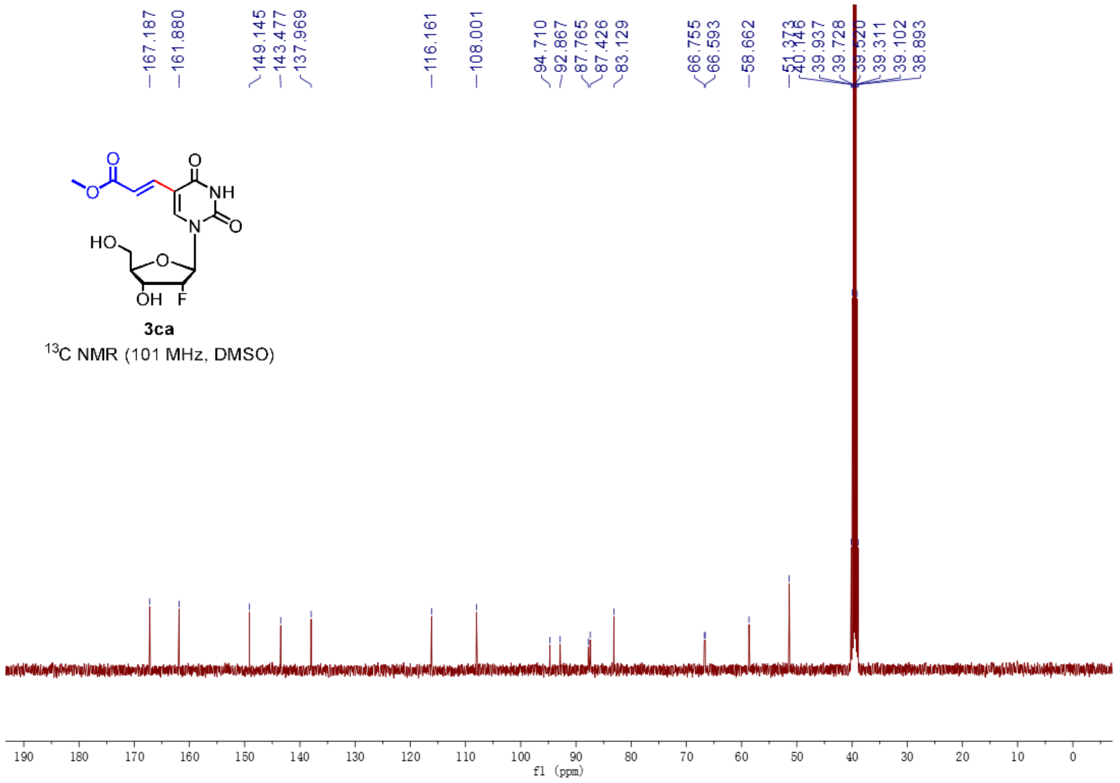

167.187
161.880
149.145
143.477
137.969
116.161
108.001
94.710
92.867
87.765
87.426
83.129
66.755
66.593
58.662
51.373
40.146
39.937
39.728
39.520
39.311
39.102
38.893
3ca
13C NMR (101 MHz, DMSO)
f1 (ppm)

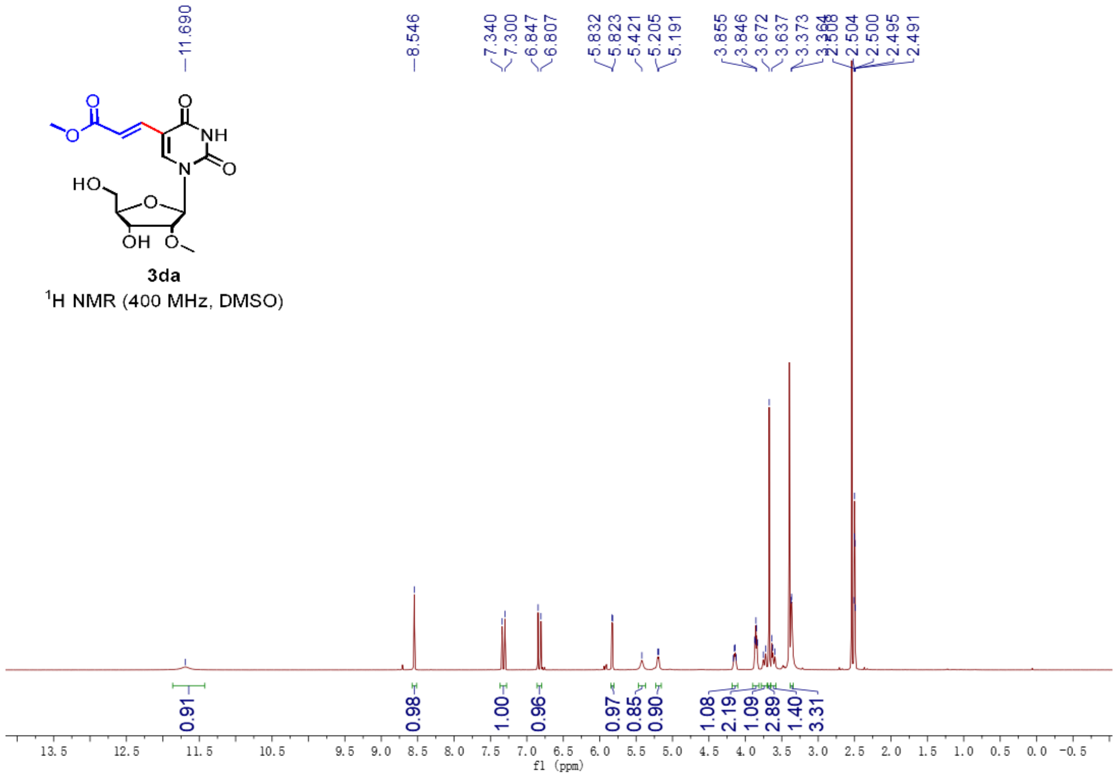

11.690
8.546
7.340
7.300
6.847
6.807
5.832
5.823
5.421
5.205
5.191
3.855
3.846
3.672
3.637
3.373
2.504
2.500
2.495
2.491
3da
1H NMR (400 MHz, DMSO)
0.91
0.98
1.00
0.96
0.97
0.85
0.90
1.08
2.19
1.09
2.89
1.40
3.31
f1 (ppm)

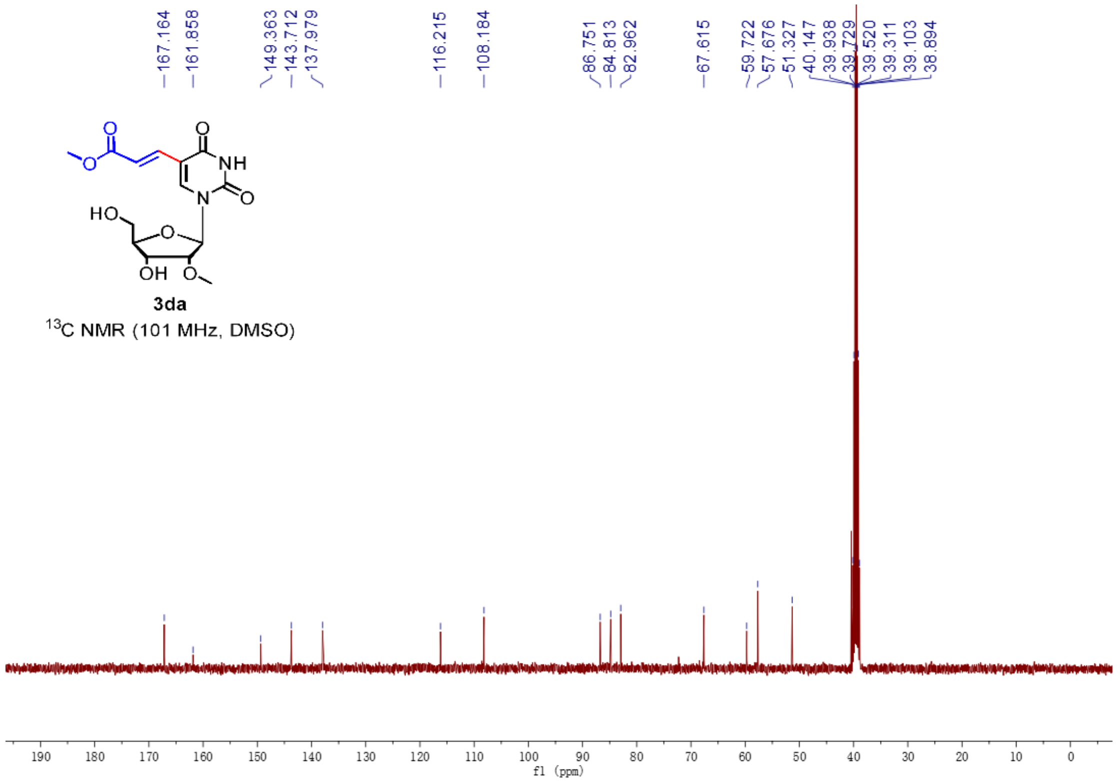
3da
^{13}C NMR (101 MHz, DMSO)
f1 (ppm)

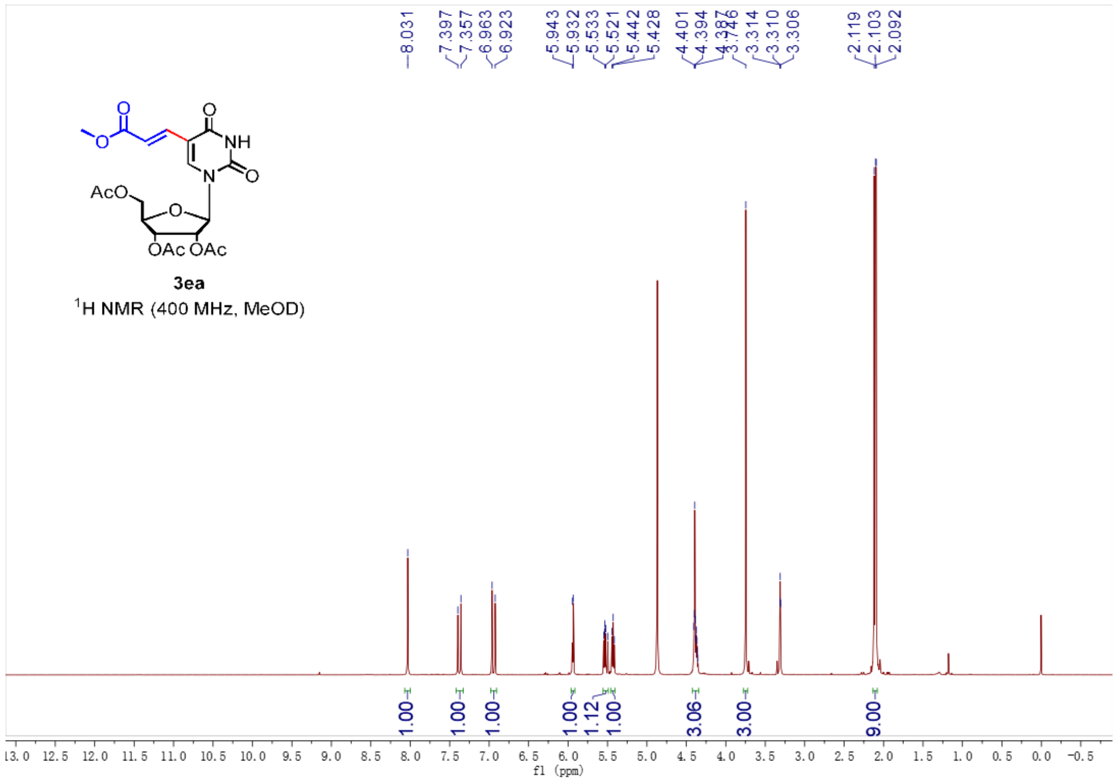
3ea
^{1}H NMR (400 MHz, MeOD)
f1 (ppm)

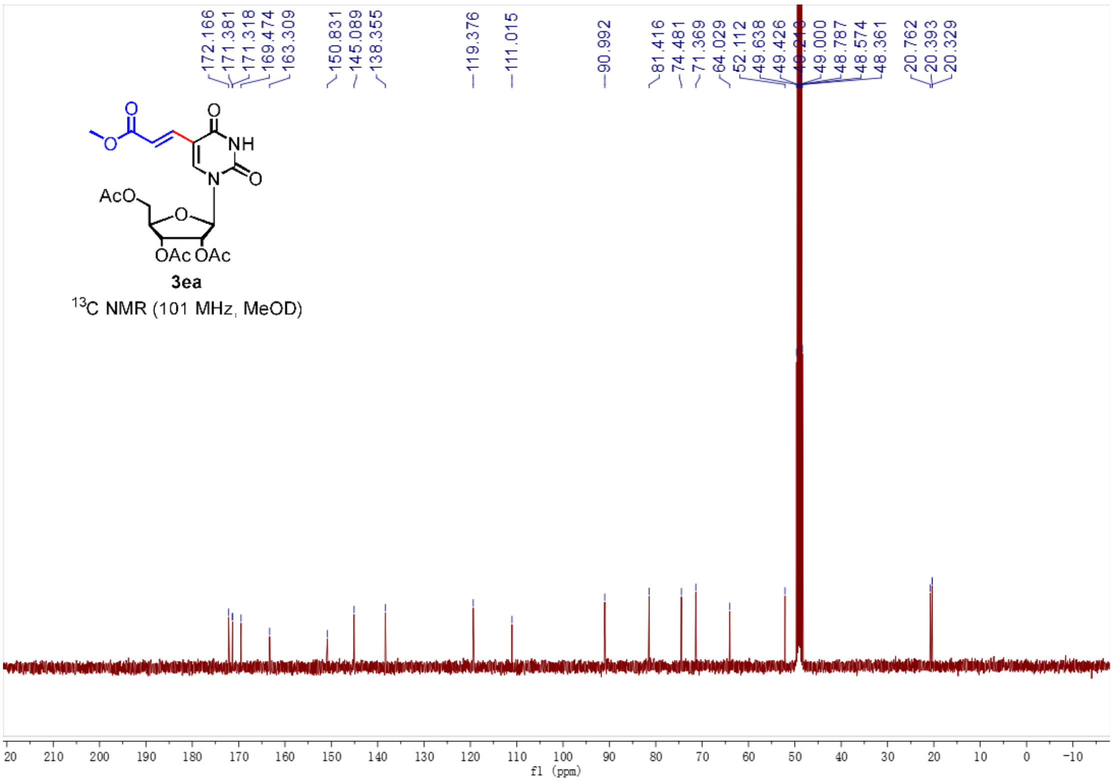
3ea
13C NMR (101 MHz, MeOD)
f1 (ppm)

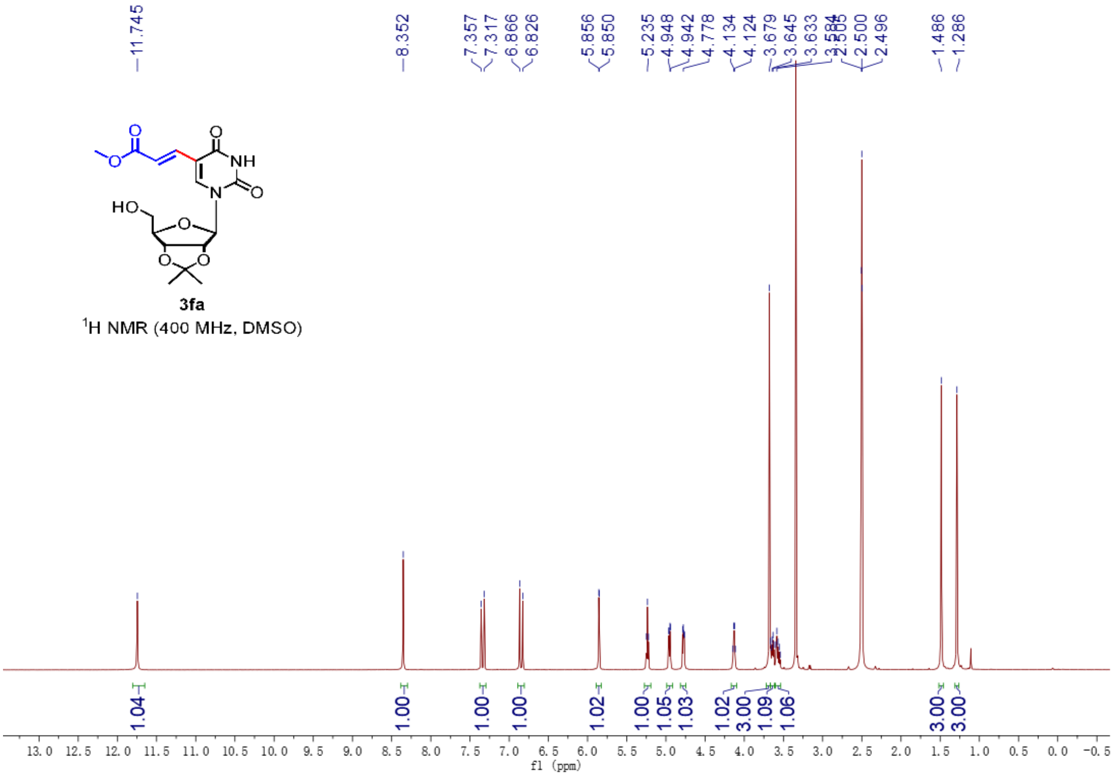
3fa
1H NMR (400 MHz, DMSO)
f1 (ppm)

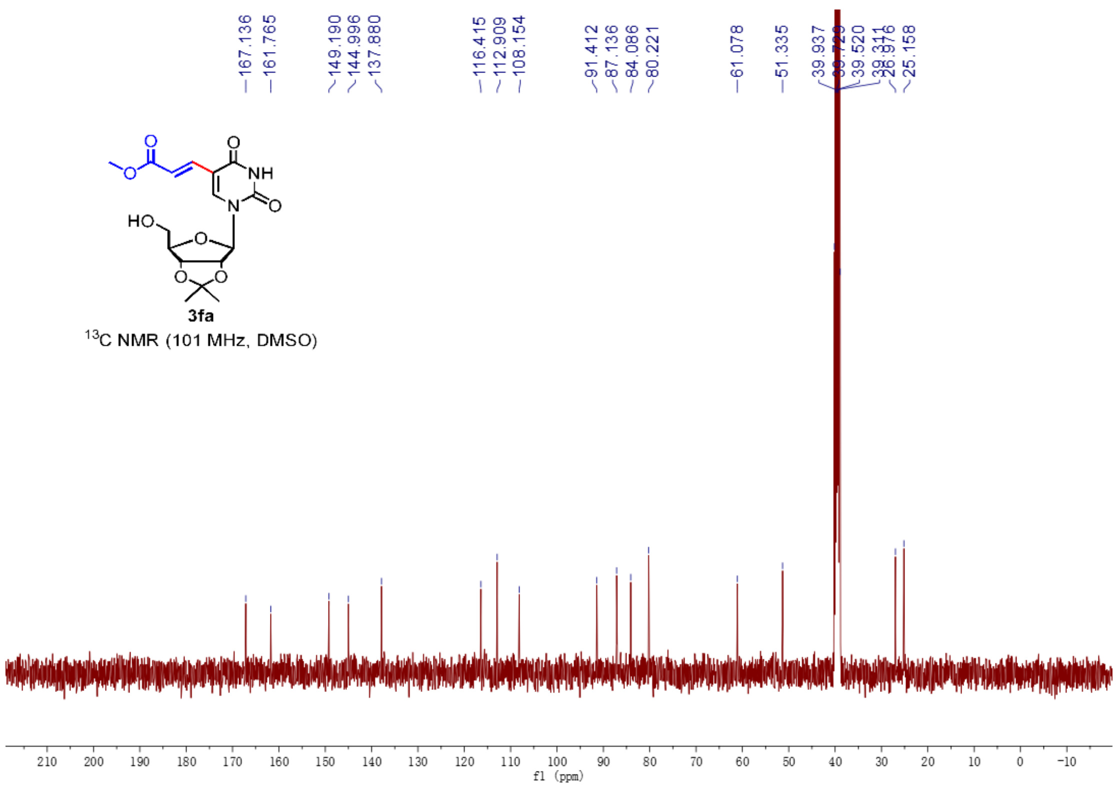
167.136
161.765
149.190
144.996
137.880
116.415
112.909
108.154
91.412
87.136
84.086
80.221
61.078
51.335
39.937
39.520
25.158
3fa
13C NMR (101 MHz, DMSO)
f1 (ppm)

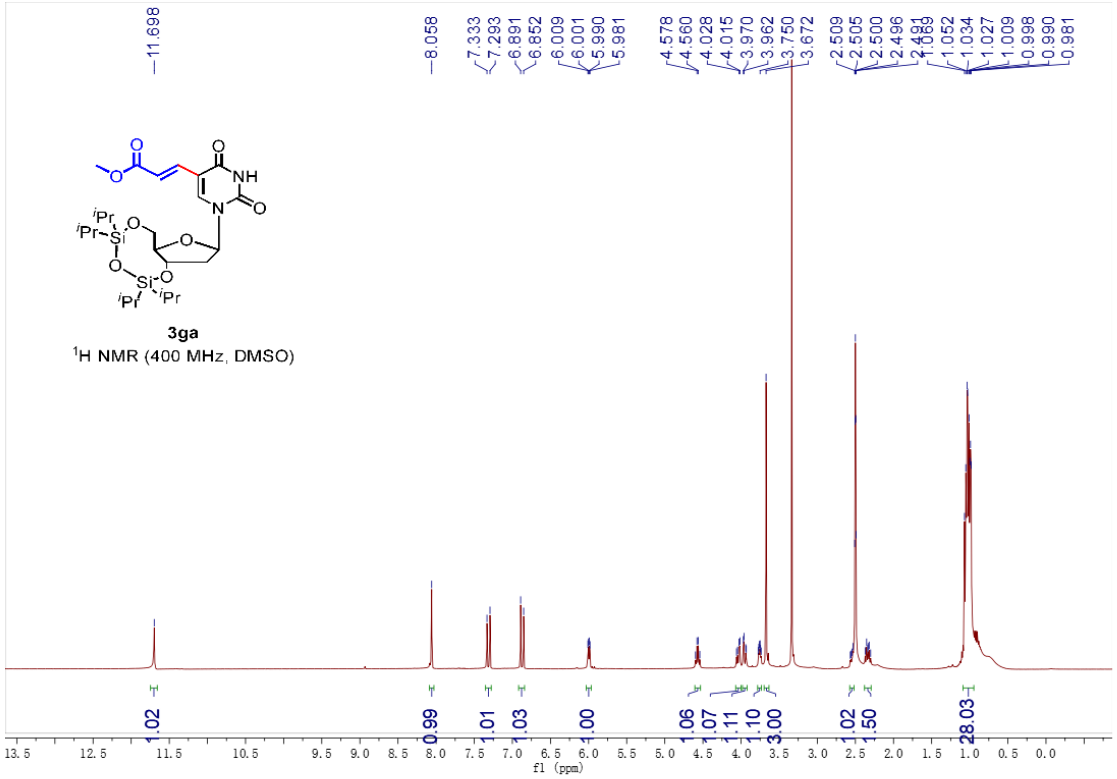
11.698
8.058
7.333
7.293
6.891
6.852
6.009
6.001
5.990
5.981
4.578
4.560
4.028
4.015
3.970
3.962
3.750
3.672
2.509
2.505
2.500
2.496
1.052
1.034
1.027
1.009
0.998
0.990
0.981
3ga
1H NMR (400 MHz, DMSO)
1.02
0.99
1.01
1.03
1.00
1.06
1.07
1.11
1.10
3.00
1.02
1.50
28.03
f1 (ppm)

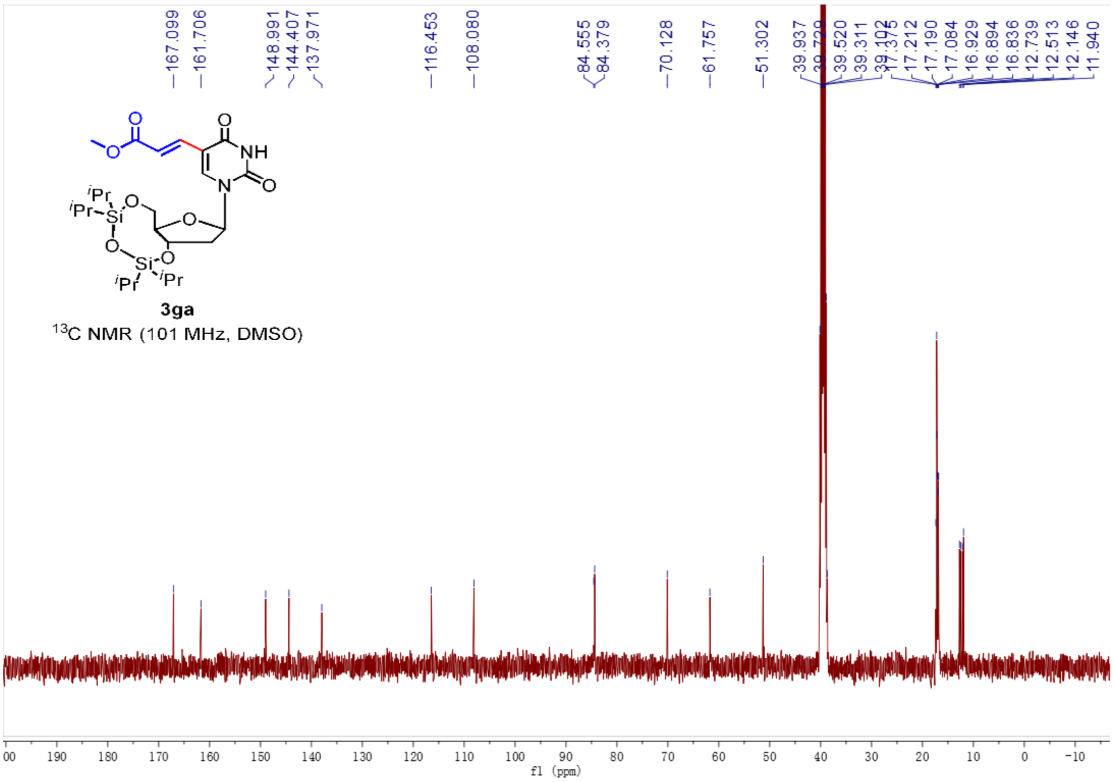
167.099
161.706
148.991
144.407
137.971
116.453
108.080
84.555
84.379
70.128
61.757
51.302
39.937
39.520
39.311
3ga
13C NMR (101 MHz, DMSO)
f1 (ppm)

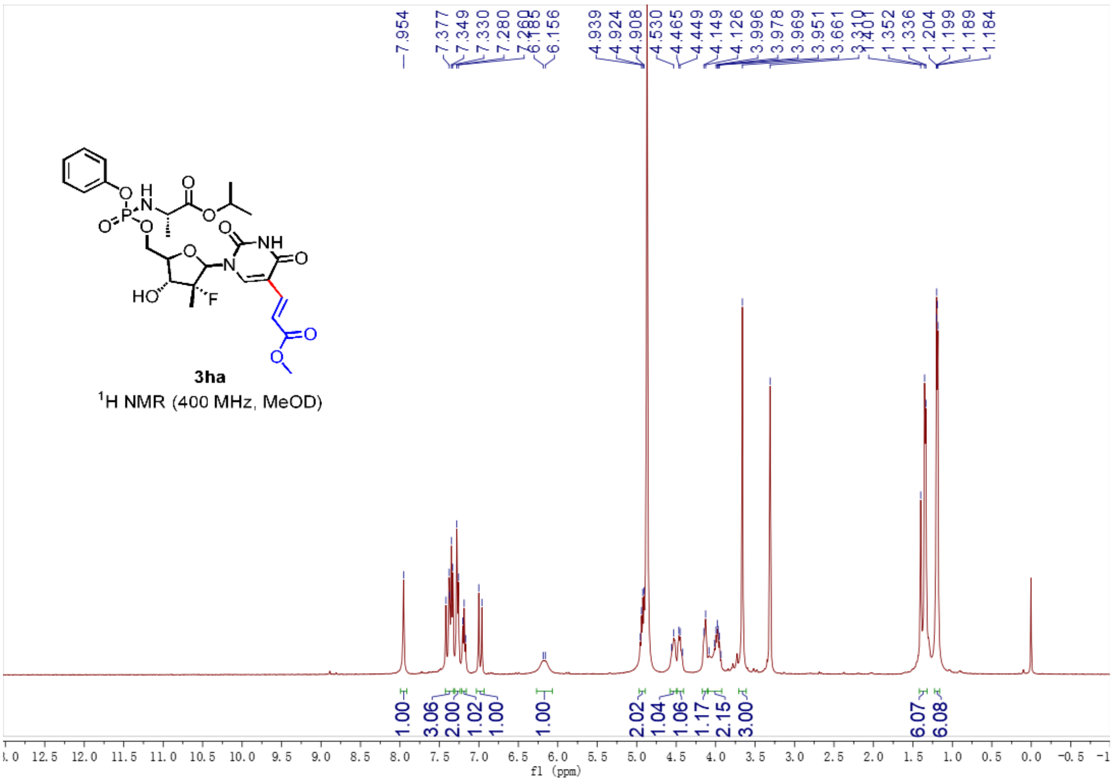
7.954
7.377
7.349
7.330
7.280
6.156
4.939
4.924
4.908
4.530
4.465
4.449
4.149
4.126
3.996
3.978
3.969
3.951
3.661
1.352
1.336
1.204
1.199
1.189
1.184
3ha
1H NMR (400 MHz, MeOD)
f1 (ppm)

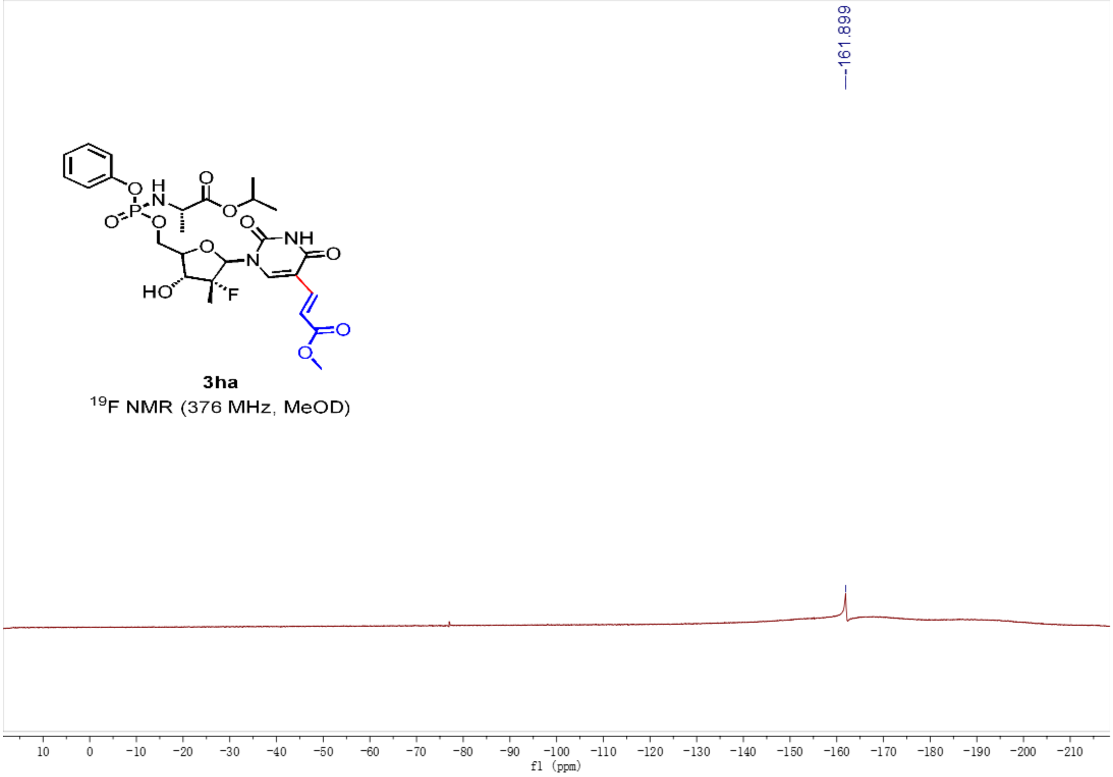

161.899
3ha
19F NMR (376 MHz, MeOD)
HO
F
NH
f1 (ppm)

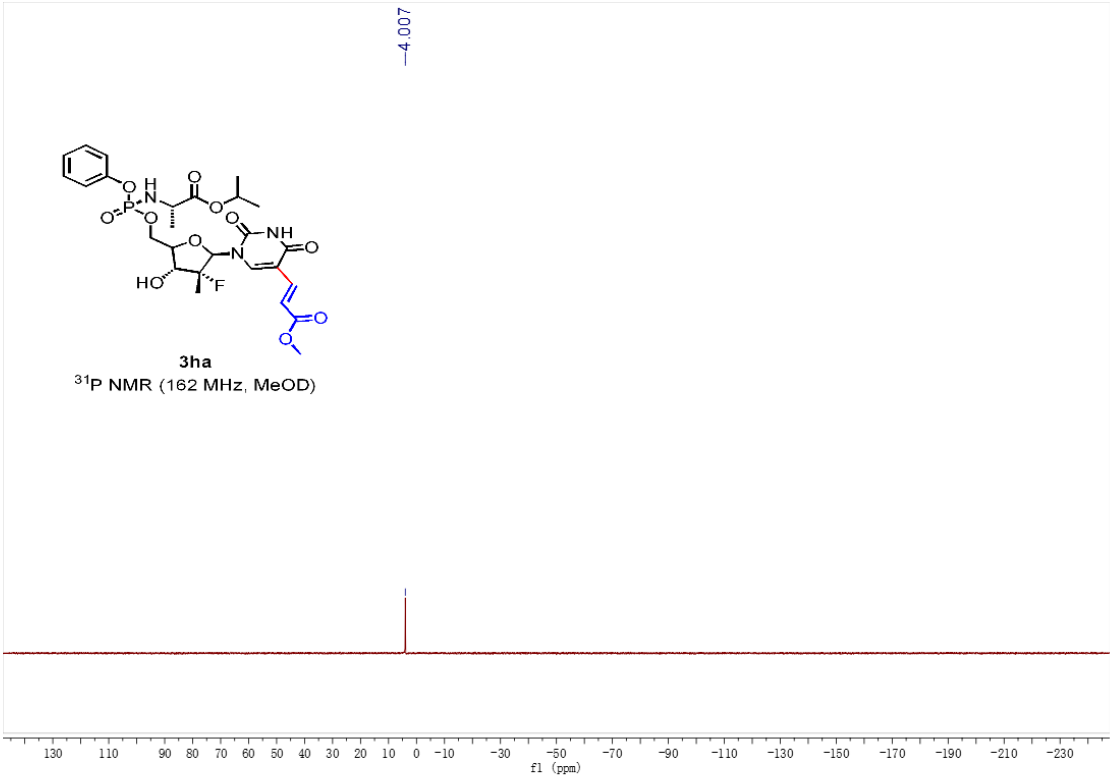

-4.007
3ha
31P NMR (162 MHz, MeOD)
HO
F
NH
f1 (ppm)

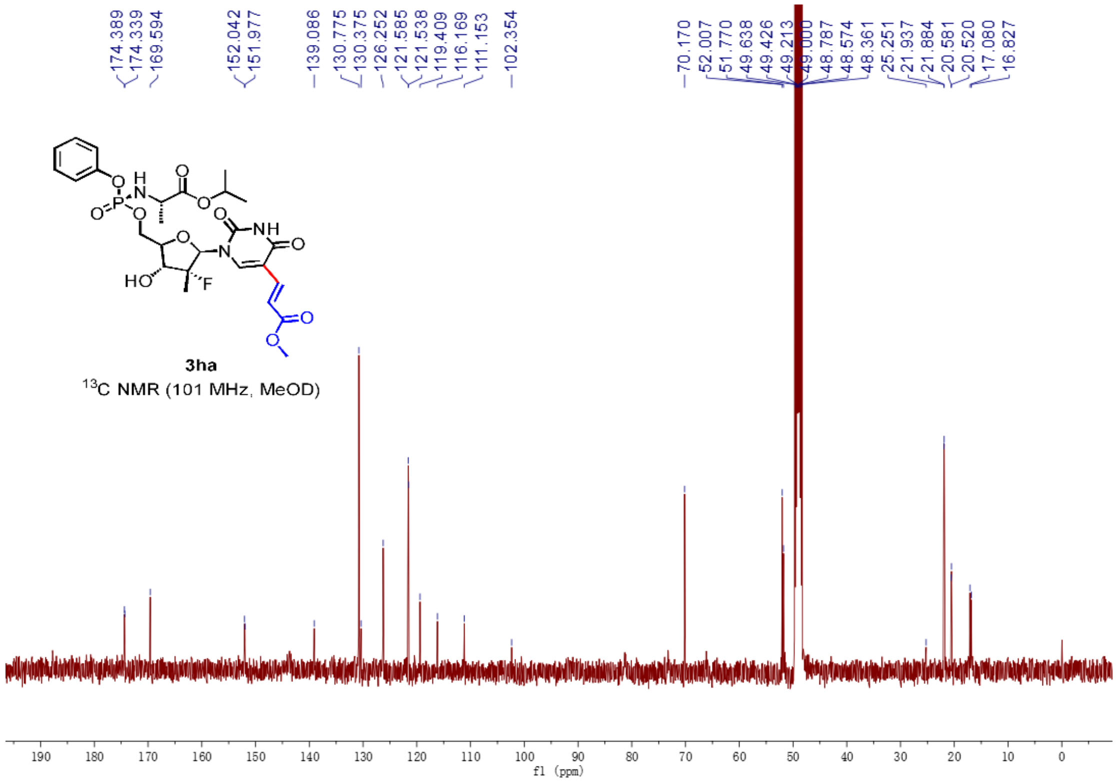

3ha

^{13}C NMR (101 MHz, MeOD)

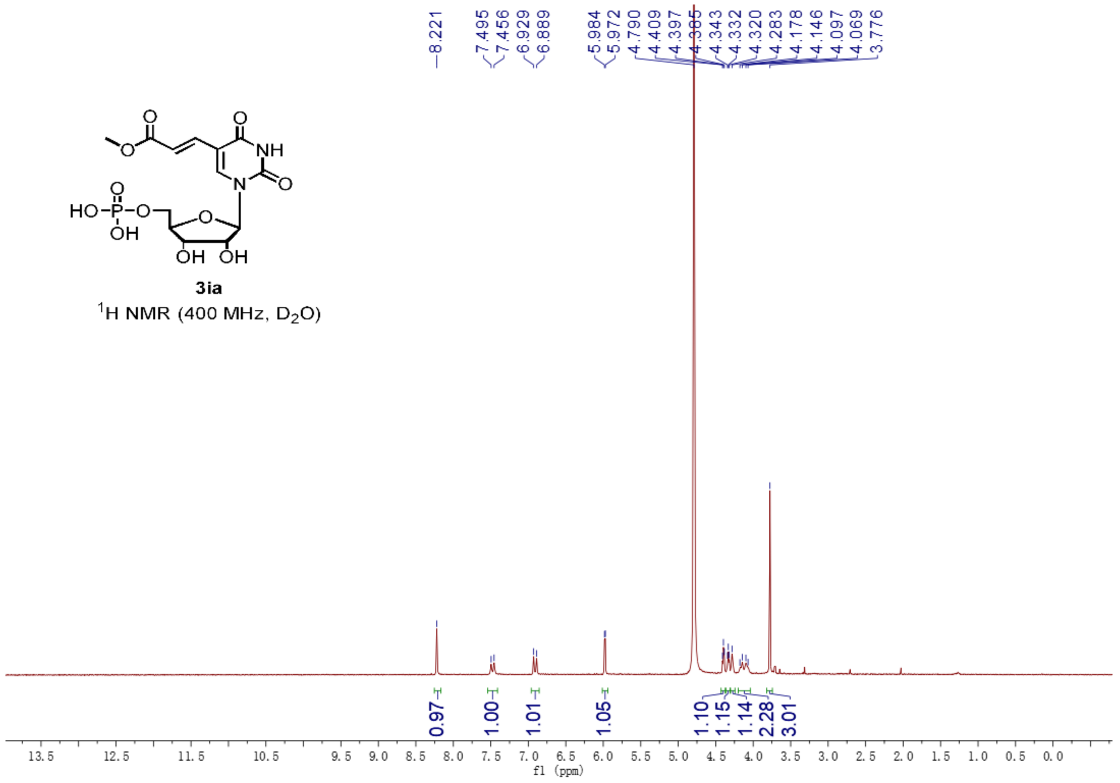

3ia

^{1}H NMR (400 MHz, D_2O)

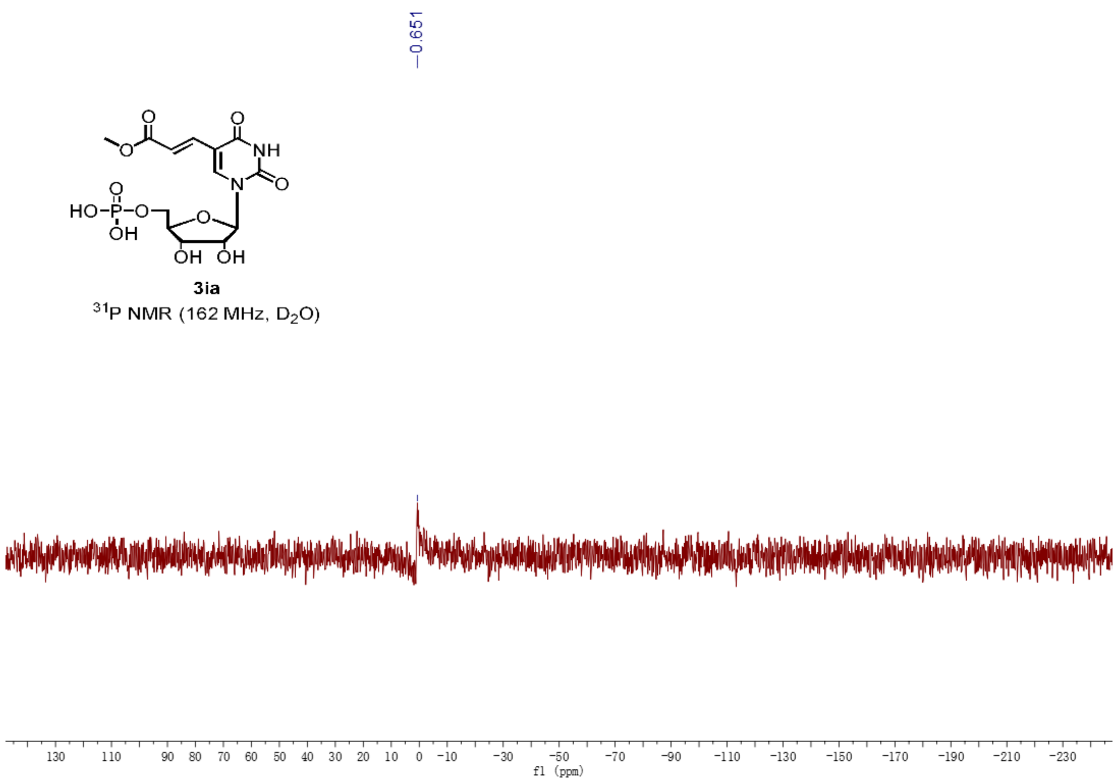
0.651
3ia
31P NMR (162 MHz, D2O)
f1 (ppm)

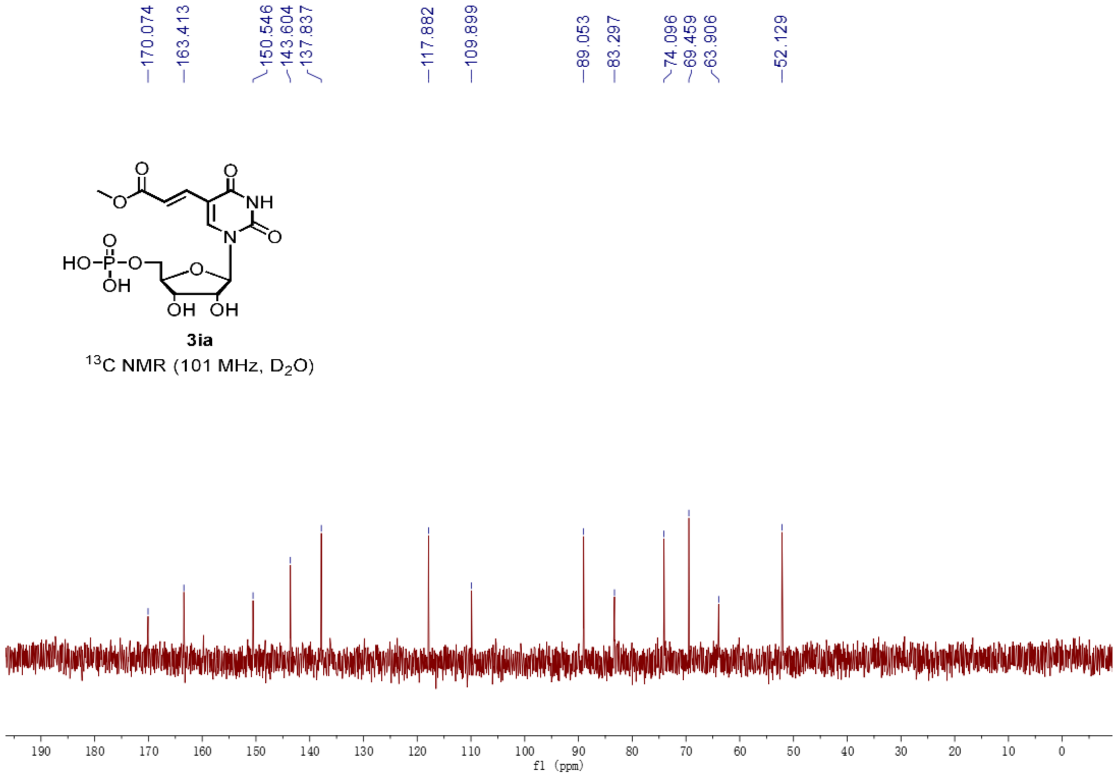
170.074
163.413
150.546
143.604
137.837
117.882
109.899
89.053
83.297
74.096
69.459
63.906
52.129
3ia
13C NMR (101 MHz, D2O)
f1 (ppm)

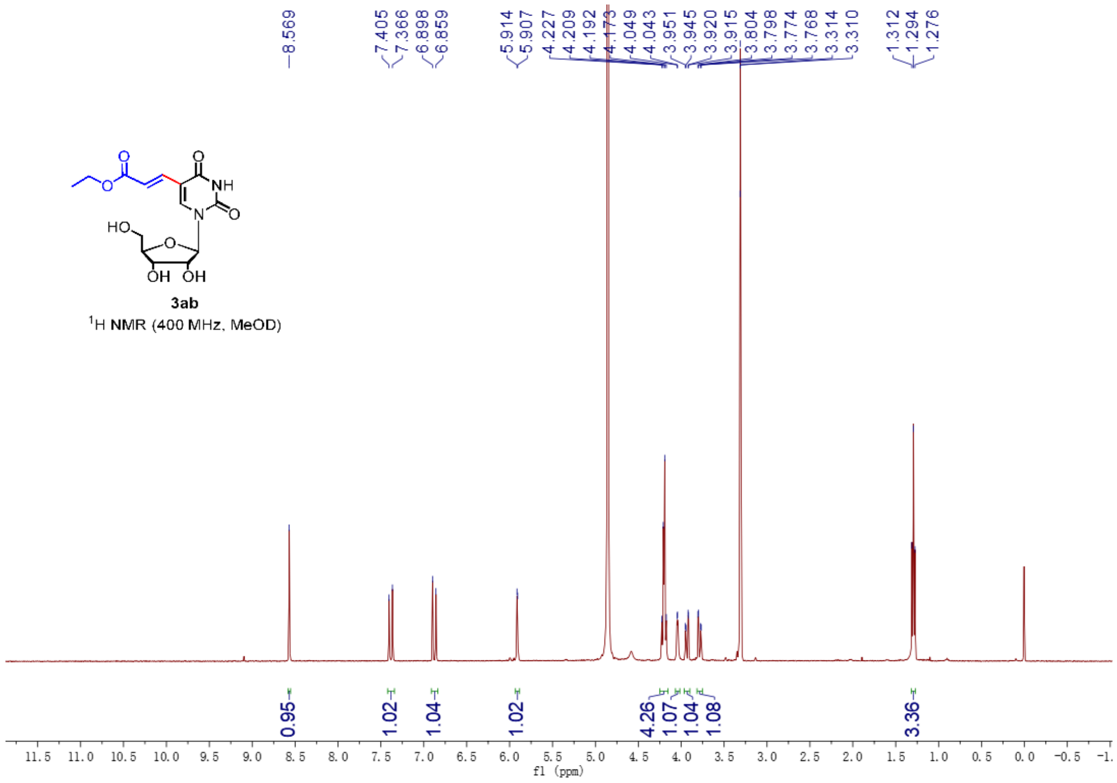
8.569
7.405
7.366
6.898
6.859
5.914
5.907
4.227
4.209
4.192
4.173
4.049
4.043
3.951
3.945
3.920
3.915
3.804
3.798
3.774
3.768
3.314
3.310
1.312
1.294
1.276
O
NH
N
O
O
O
HO
O
OH OH
3ab
1H NMR (400 MHz, MeOD)
0.95
1.02
1.04
1.02
4.26
1.07
1.04
1.08
3.36
f1 (ppm)

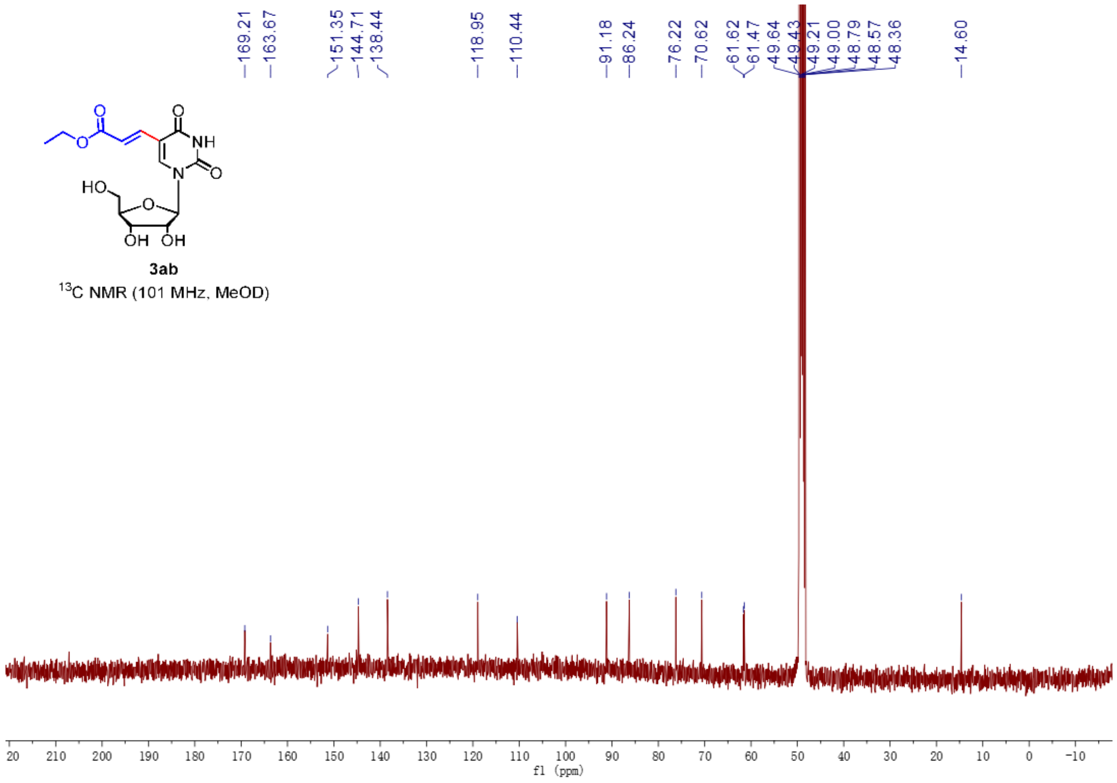
169.21
163.67
151.35
144.71
138.44
118.95
110.44
91.18
86.24
76.22
70.62
61.62
61.47
49.64
49.43
49.21
49.00
48.79
48.57
48.36
14.60
O
NH
N
O
O
O
HO
O
OH OH
3ab
13C NMR (101 MHz, MeOD)
f1 (ppm)

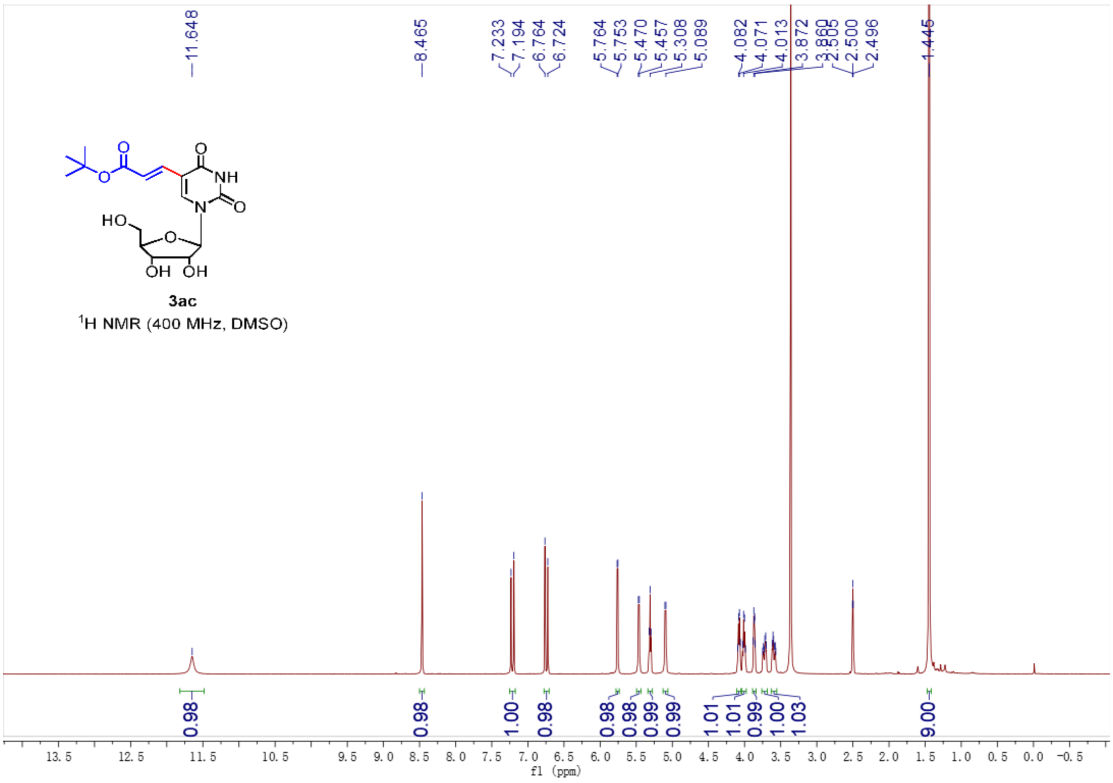
3ac
1H NMR (400 MHz, DMSO)
11.648
8.465
7.233
7.194
6.764
6.724
5.764
5.753
5.470
5.457
5.308
5.089
4.082
4.071
4.013
3.872
2.505
2.500
2.496
1.445
0.98
0.98
1.00
0.98
0.98
0.98
0.99
0.99
1.01
1.01
0.99
1.00
1.03
9.00
f1 (ppm)

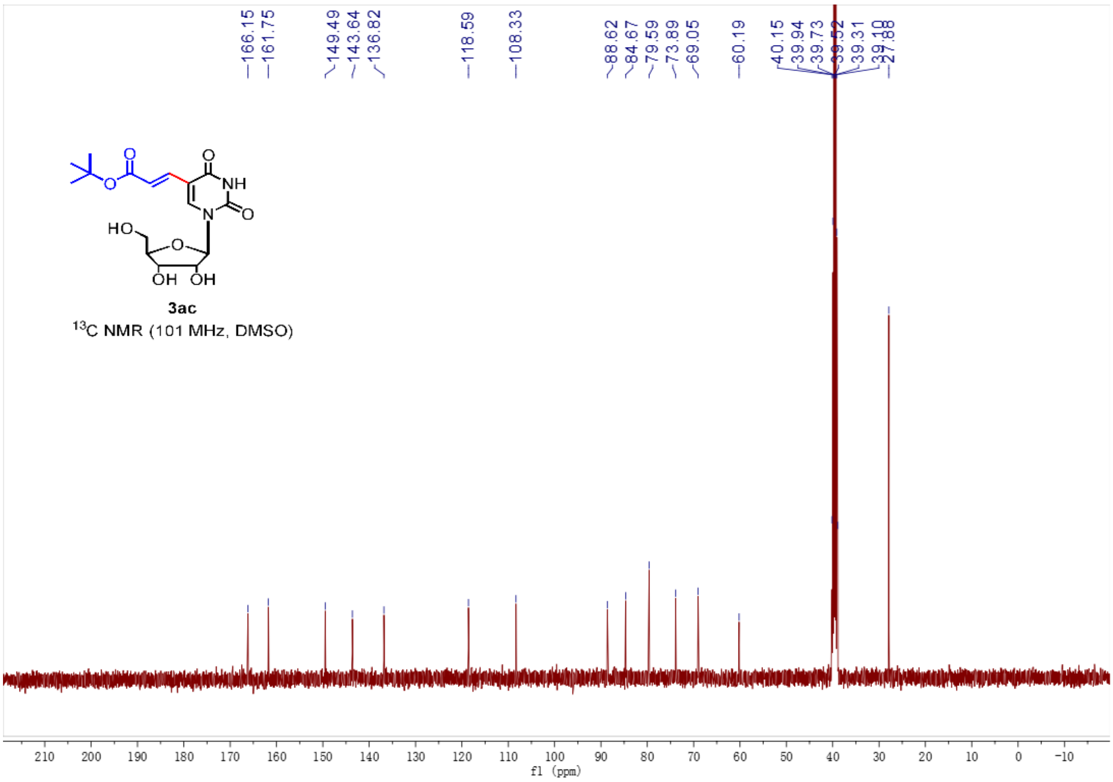
3ac
13C NMR (101 MHz, DMSO)
166.15
161.75
149.49
143.64
136.82
118.59
108.33
88.62
84.67
79.59
73.89
69.05
60.19
40.15
39.94
39.73
39.31
27.88
f1 (ppm)

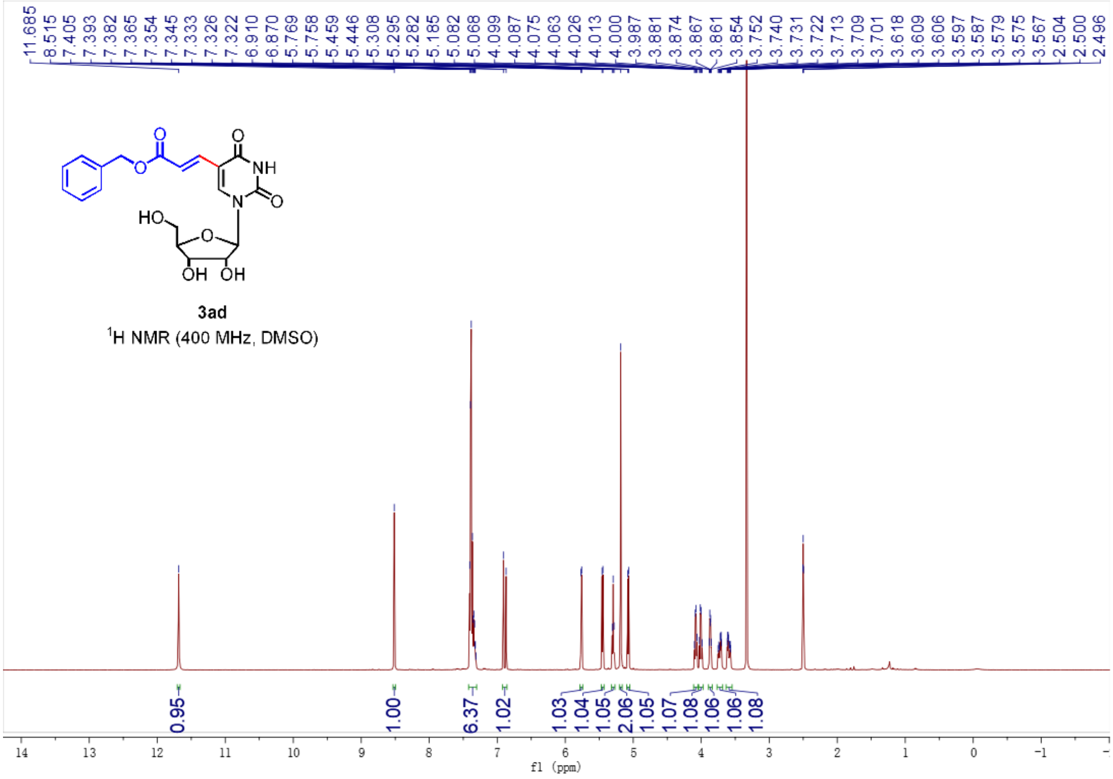
3ad
1H NMR (400 MHz, DMSO)
f1 (ppm)

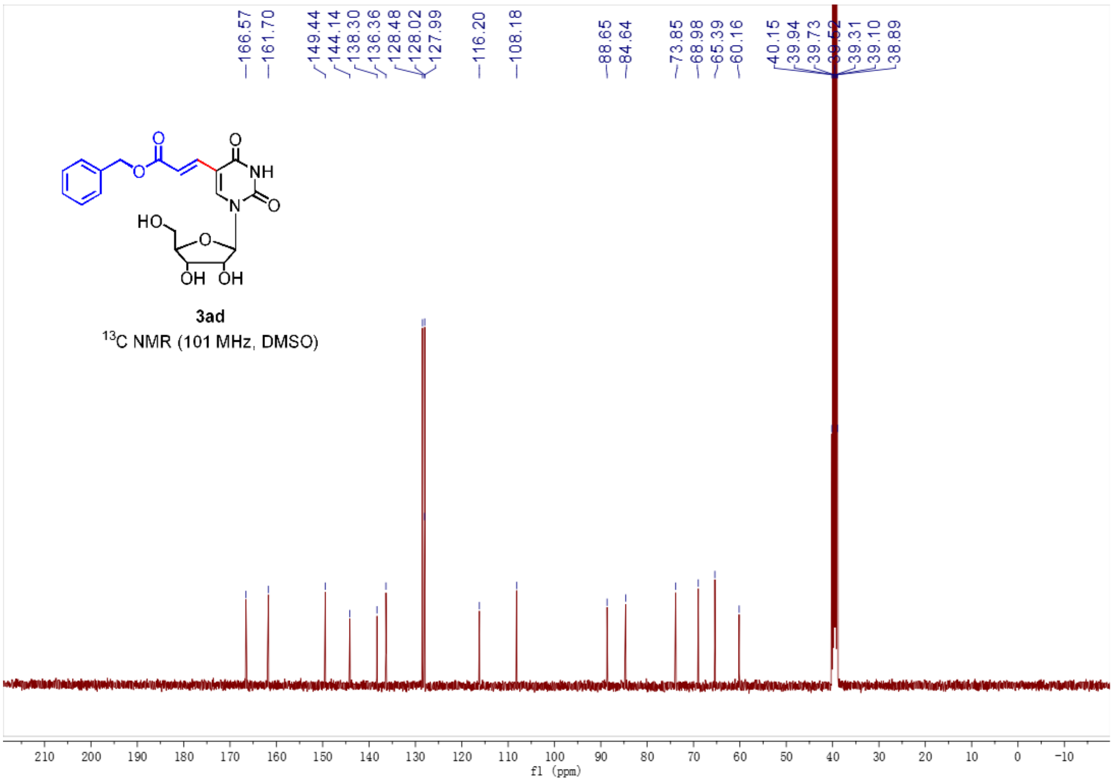
3ad
13C NMR (101 MHz, DMSO)
f1 (ppm)

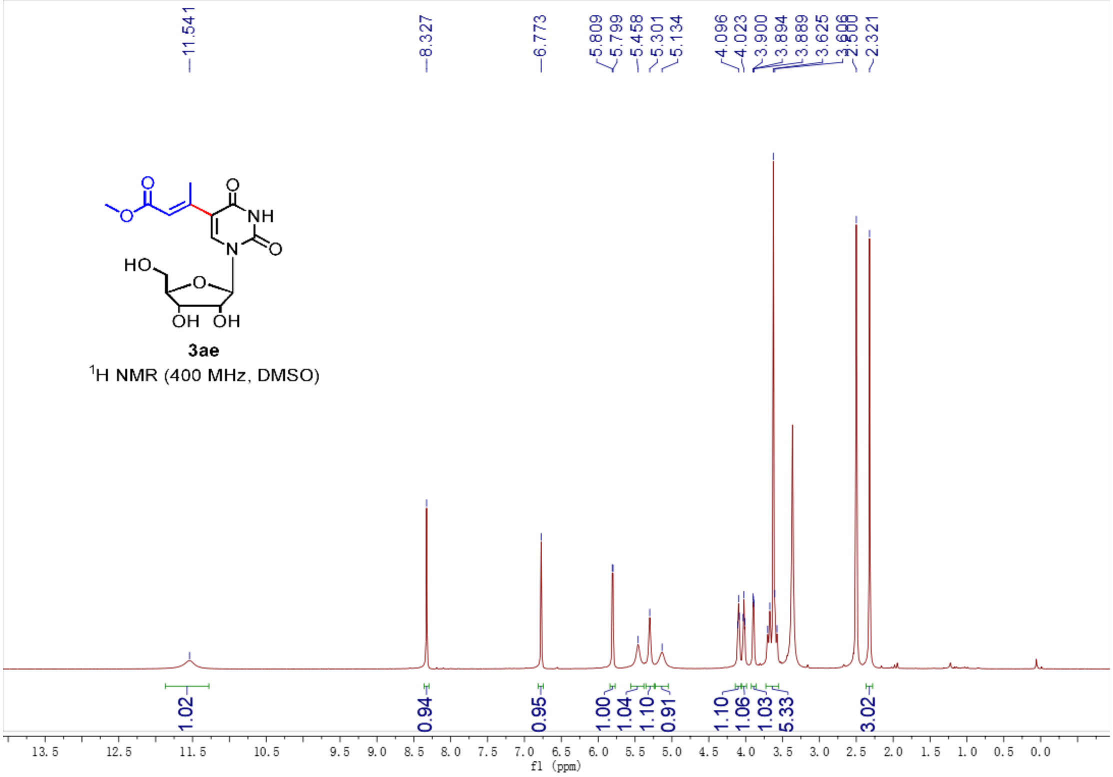
3ae
1H NMR (400 MHz, DMSO)
f1 (ppm)

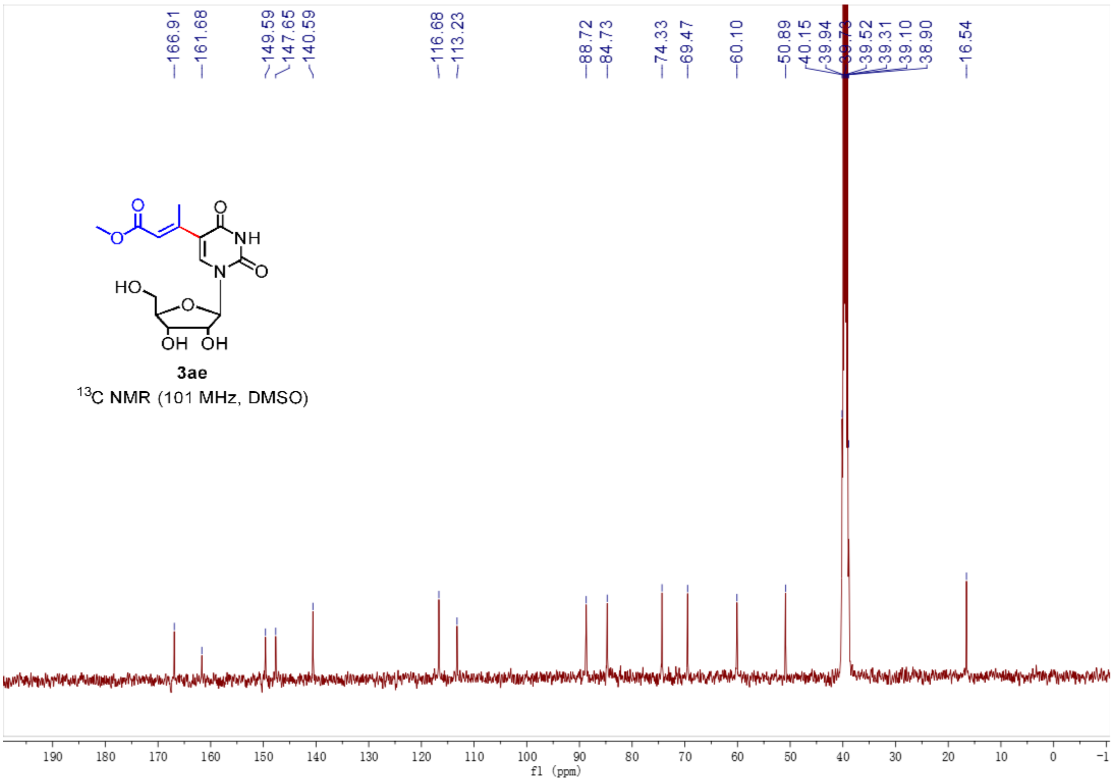
3ae
13C NMR (101 MHz, DMSO)
f1 (ppm)

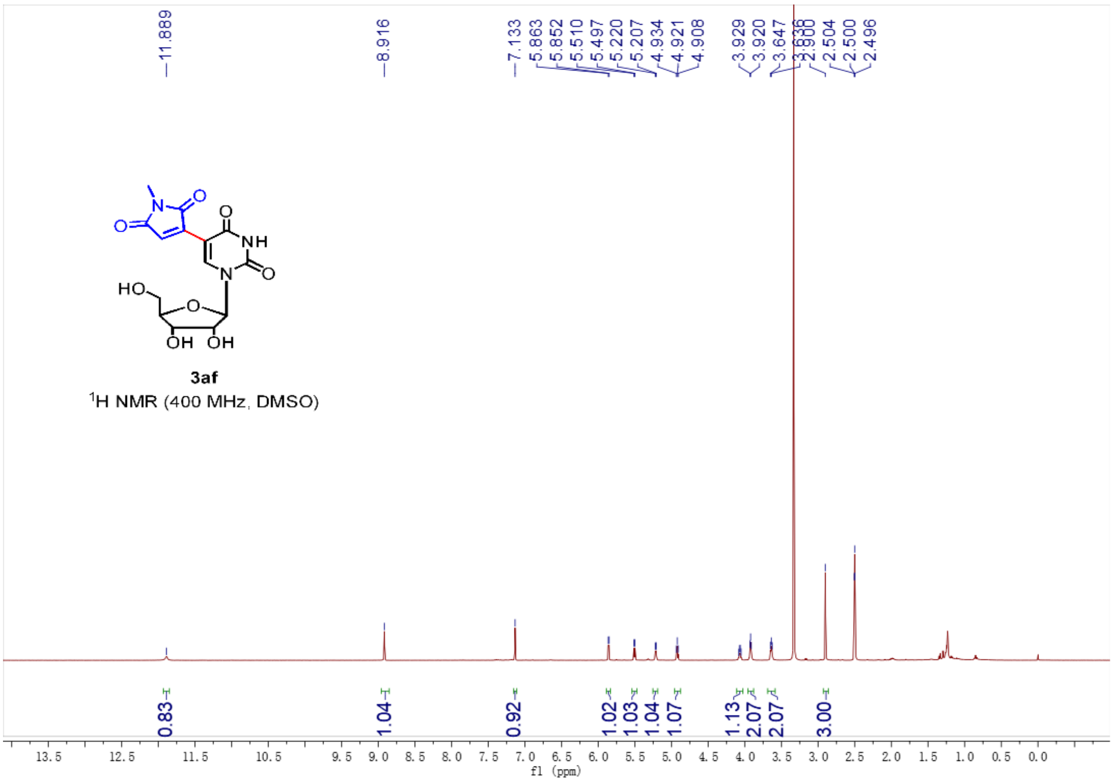
3af
1H NMR (400 MHz, DMSO)
f1 (ppm)

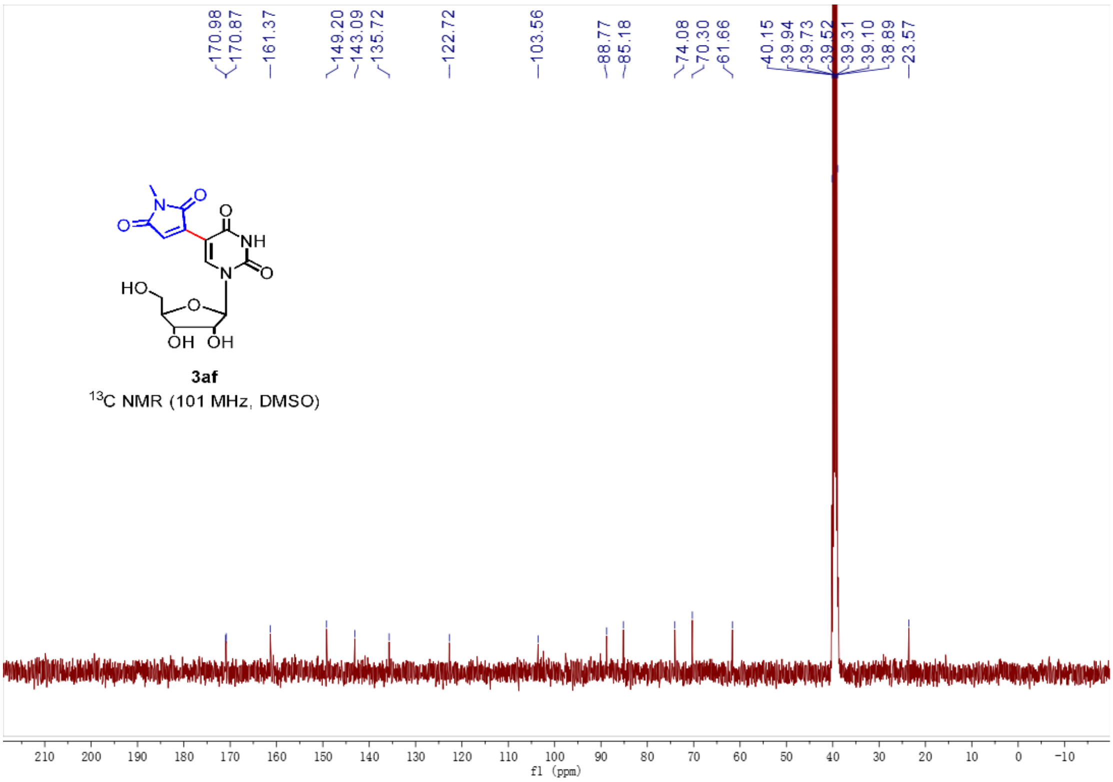
3af
13C NMR (101 MHz, DMSO)
f1 (ppm)

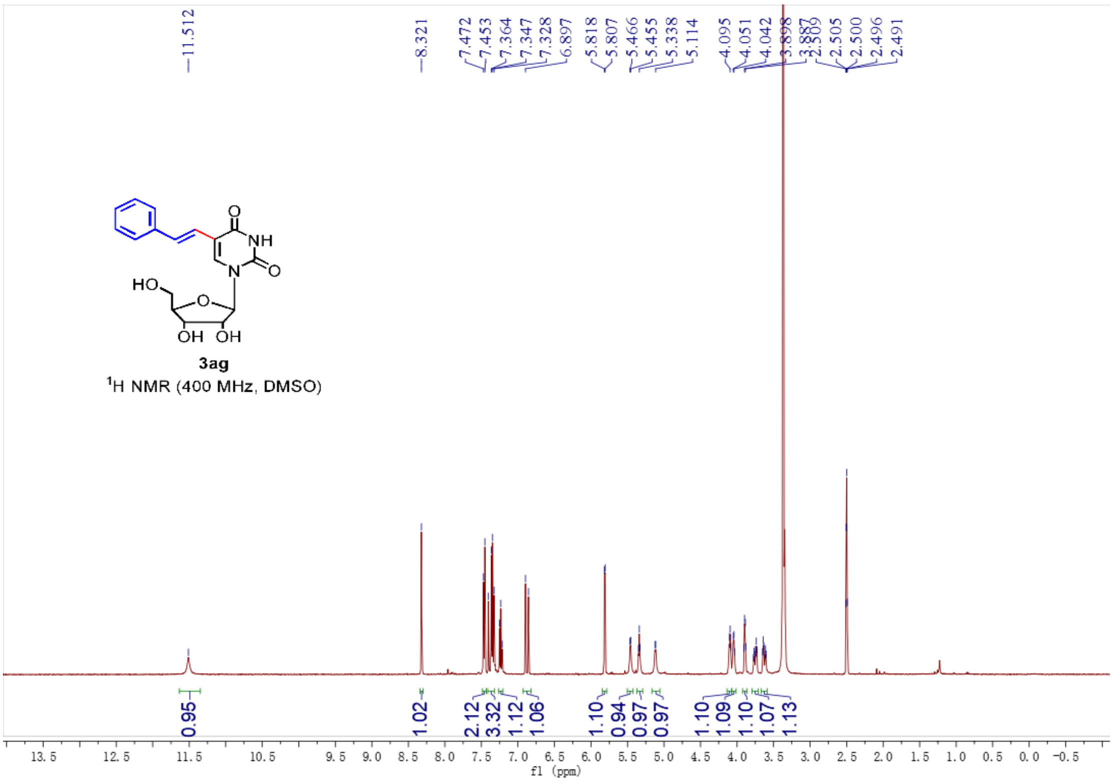

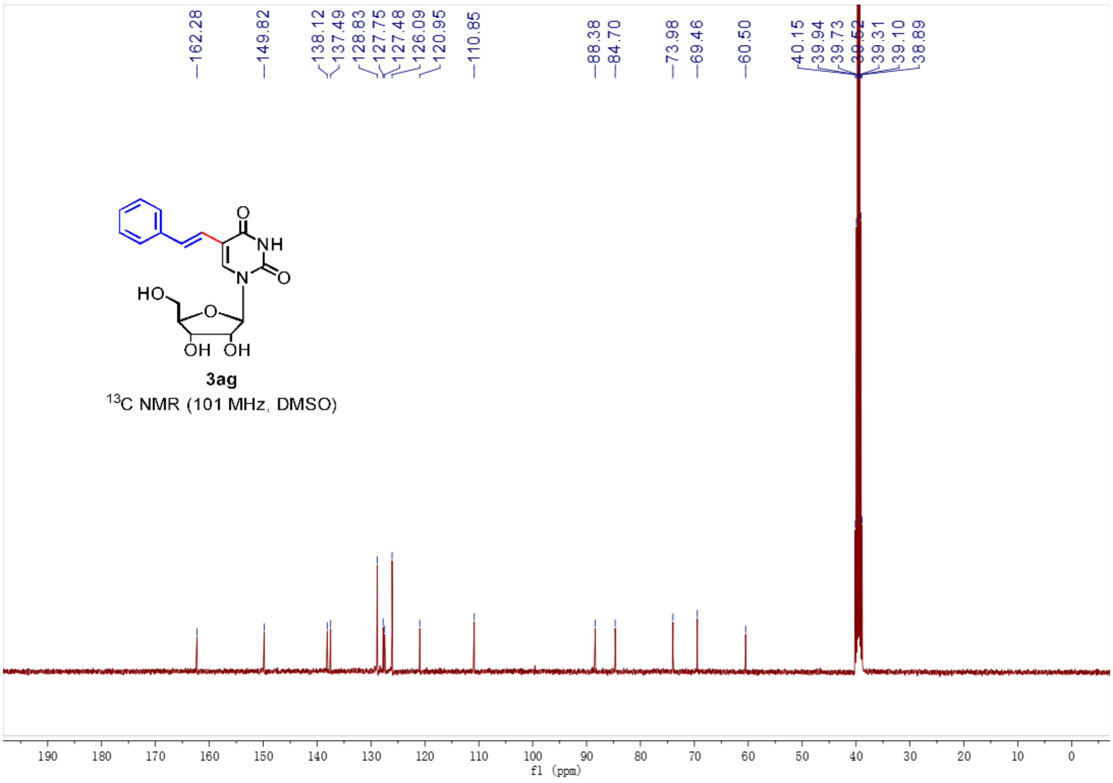

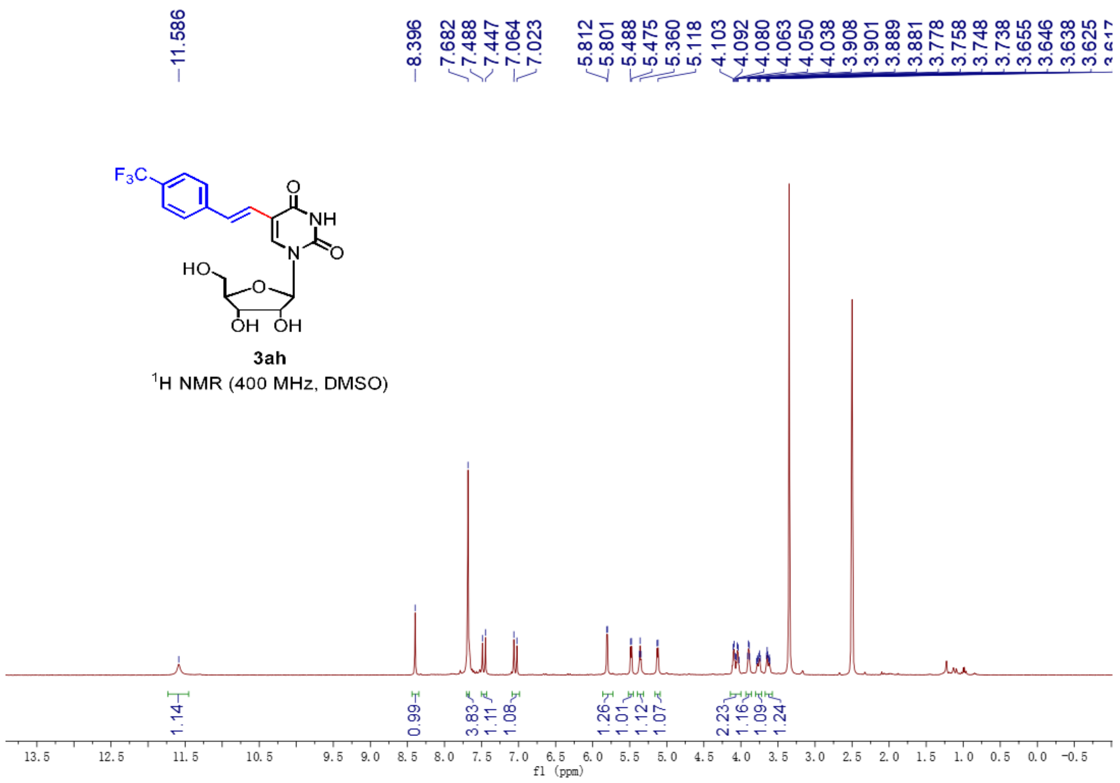
11.586
8.396
7.682
7.488
7.447
7.064
7.023
5.812
5.801
5.488
5.475
5.360
5.118
4.103
4.092
4.080
4.063
4.050
4.038
3.908
3.901
3.889
3.881
3.778
3.758
3.748
3.738
3.655
3.646
3.638
3.625
3ah
¹H NMR (400 MHz, DMSO)
f1 (ppm)

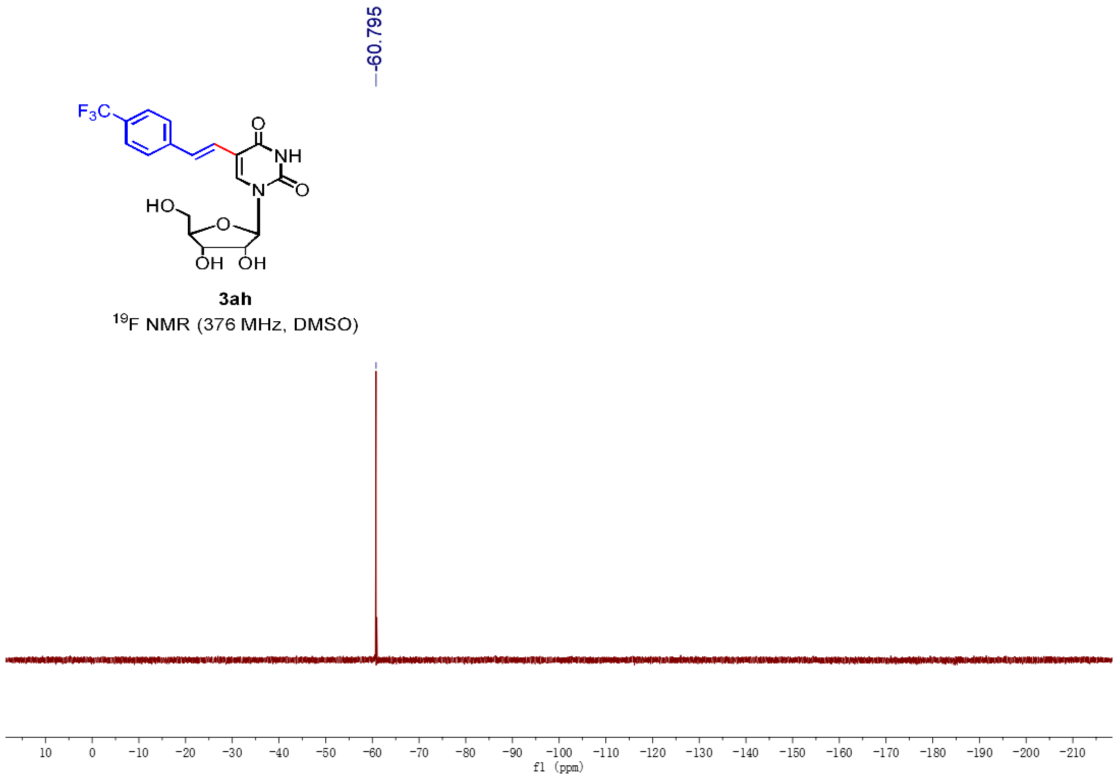
-60.795
3ah
¹⁹F NMR (376 MHz, DMSO)
f1 (ppm)

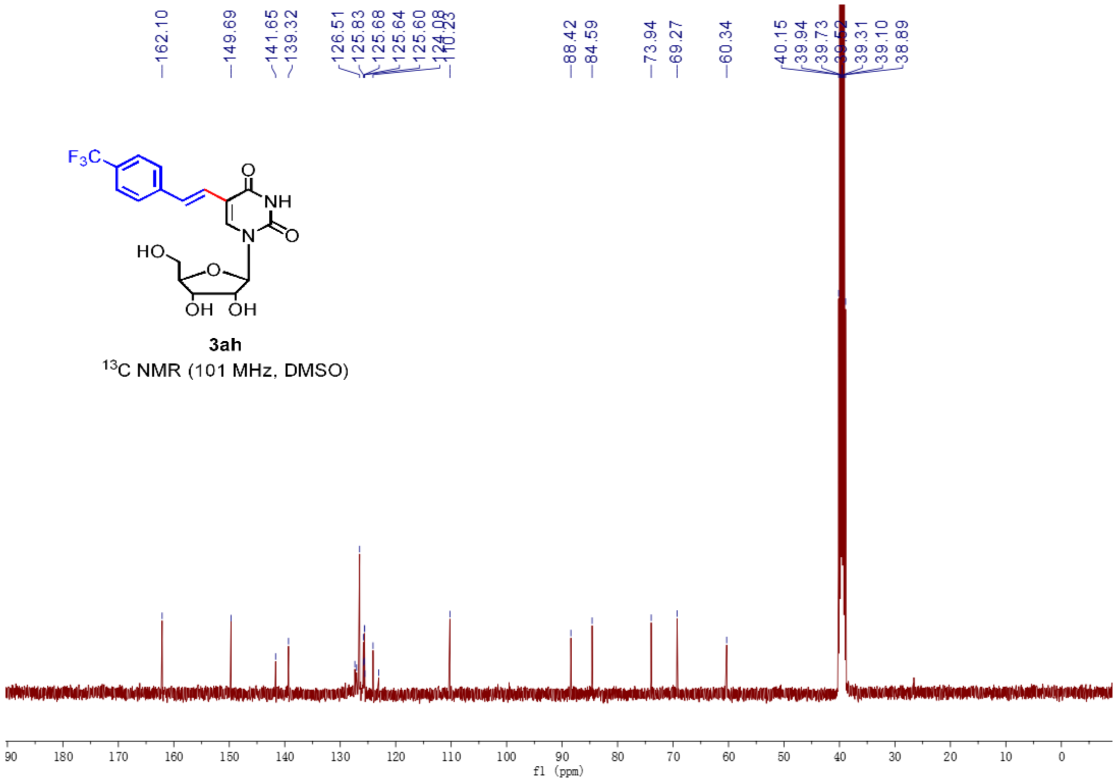

3ah

^{13}C NMR (101 MHz, DMSO)

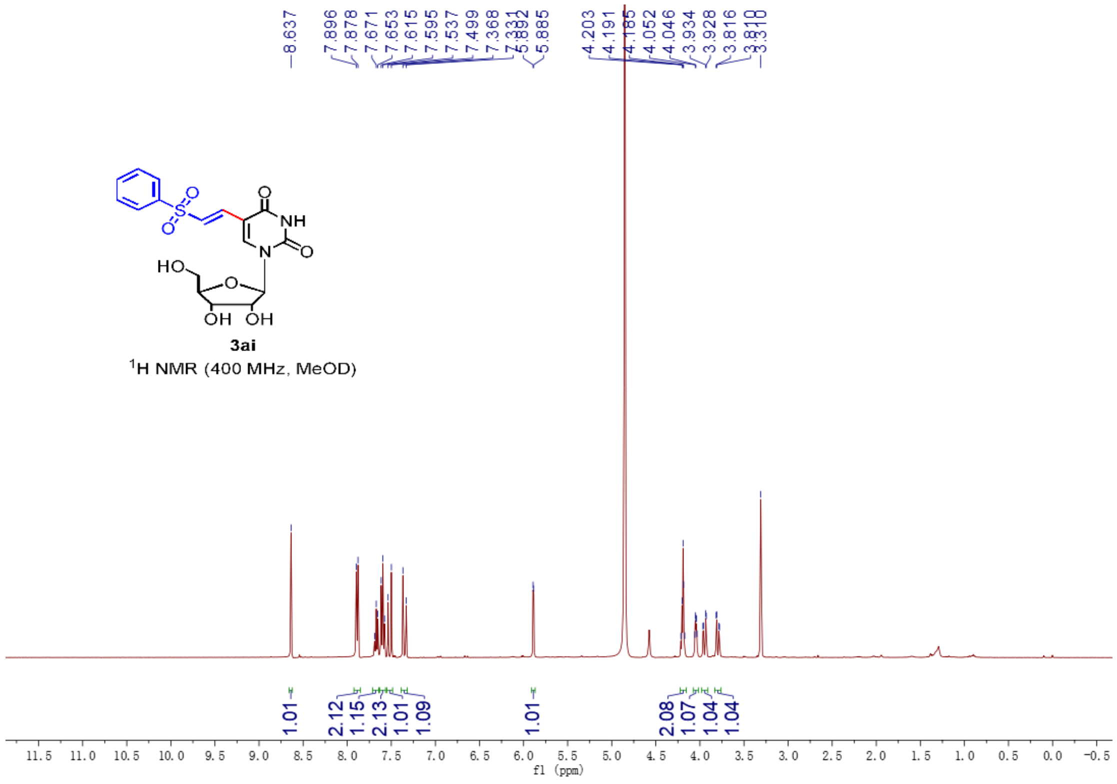

3ai

^{1}H NMR (400 MHz, MeOD)

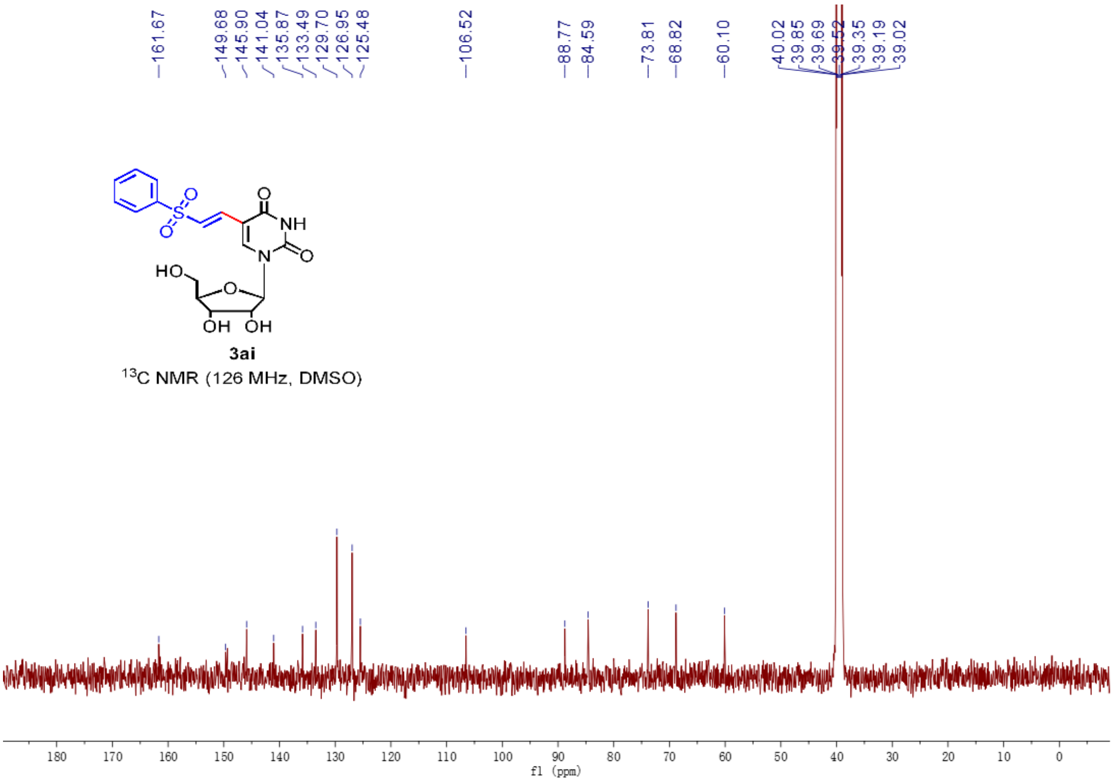
161.67
149.68
145.90
141.04
135.87
133.49
129.70
126.95
125.48
106.52
88.77
84.59
73.81
68.82
60.10
40.02
39.85
39.69
39.52
39.35
39.19
39.02
3ai
^{13}C NMR (126 MHz, DMSO)
f1 (ppm)

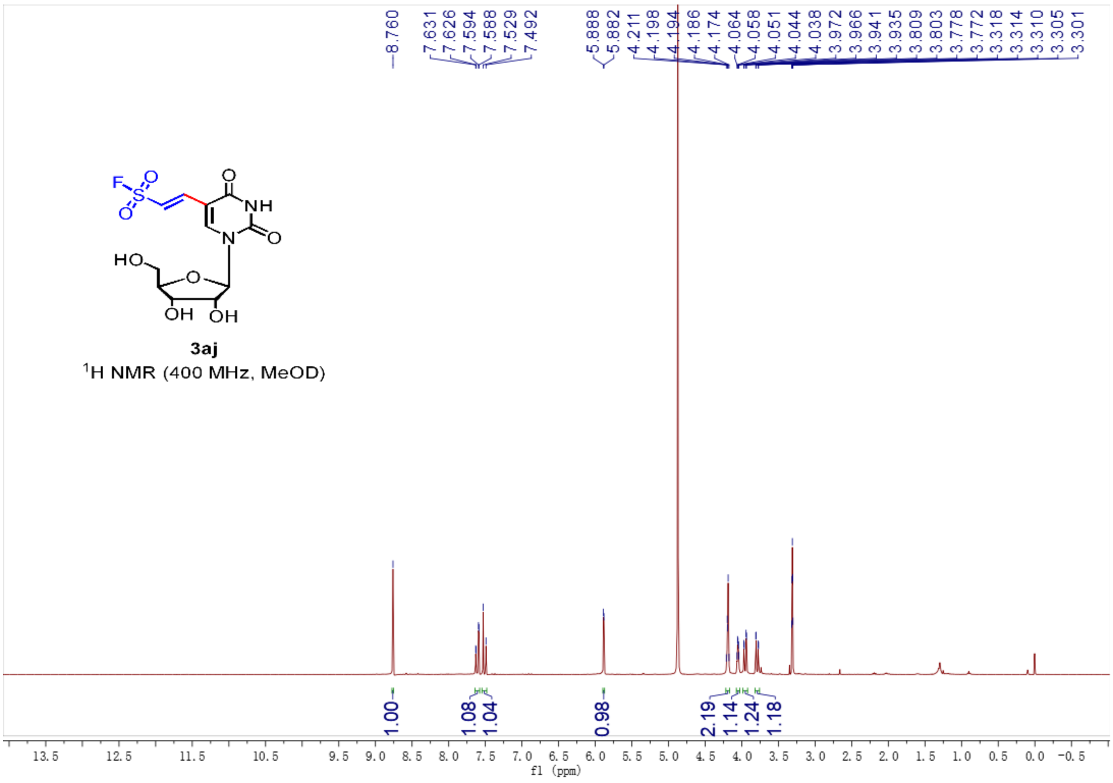
8.760
7.631
7.626
7.594
7.588
7.529
7.492
5.888
5.882
4.211
4.198
4.194
4.186
4.174
4.064
4.058
4.051
4.044
4.038
3.972
3.966
3.941
3.935
3.809
3.803
3.778
3.772
3.318
3.314
3.310
3.305
3.301
3aj
^{1}H NMR (400 MHz, MeOD)
1.00
1.08
1.04
0.98
2.19
1.14
1.24
1.18
f1 (ppm)

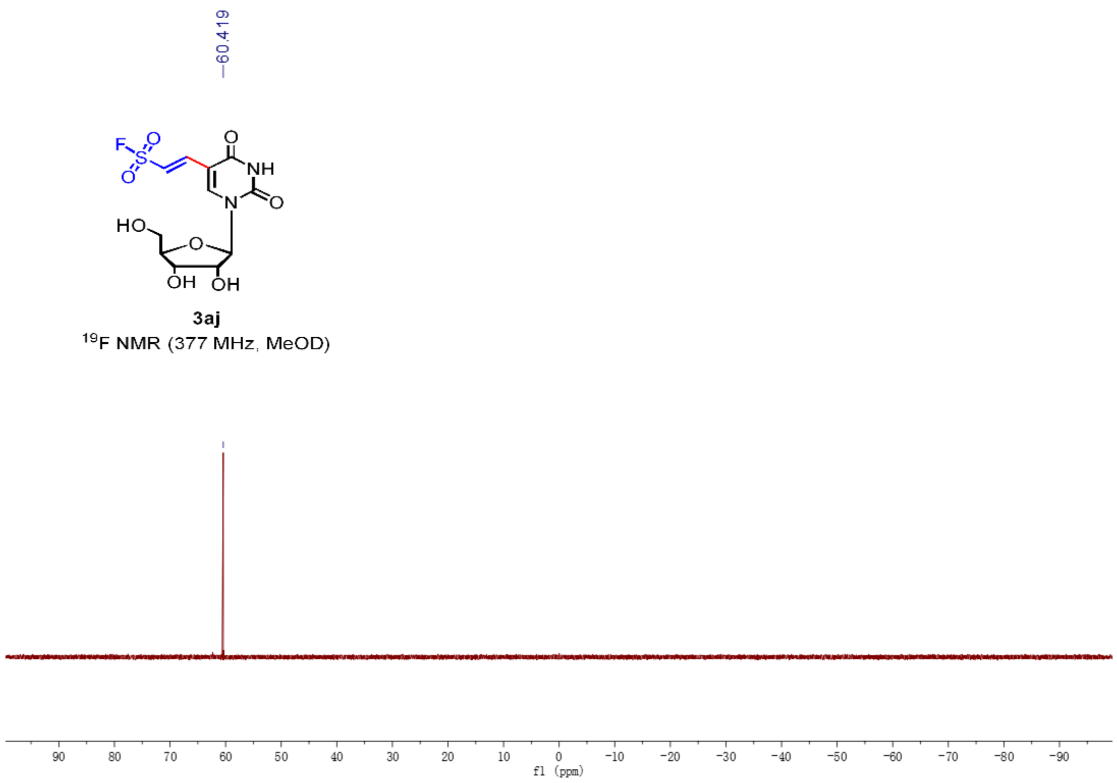

—60.419
F
S
O
O
O
NH
N
O
HO
O
OH
OH
3aj
19F NMR (377 MHz, MeOD)
f1 (ppm)

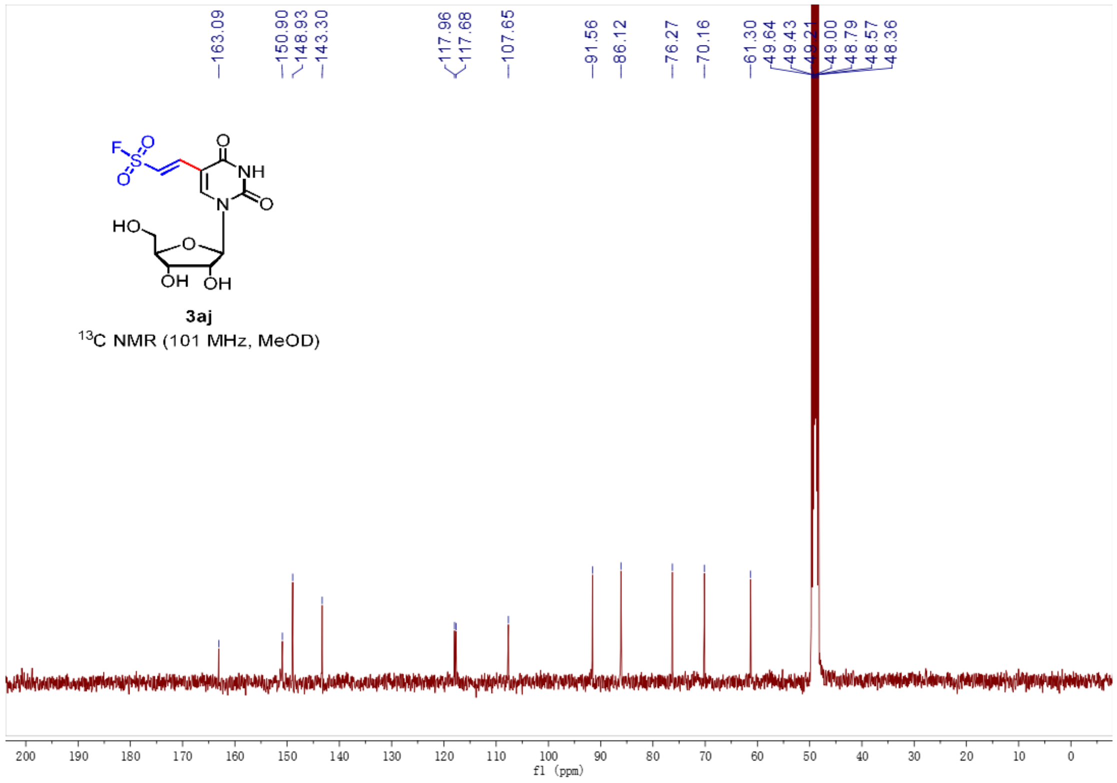

—163.09
—150.90
148.93
—143.30
117.96
117.68
—107.65
—91.56
—86.12
—76.27
—70.16
—61.30
49.64
49.43
49.21
49.00
48.79
48.57
48.36
F
S
O
O
O
NH
N
O
HO
O
OH
OH
3aj
13C NMR (101 MHz, MeOD)
f1 (ppm)

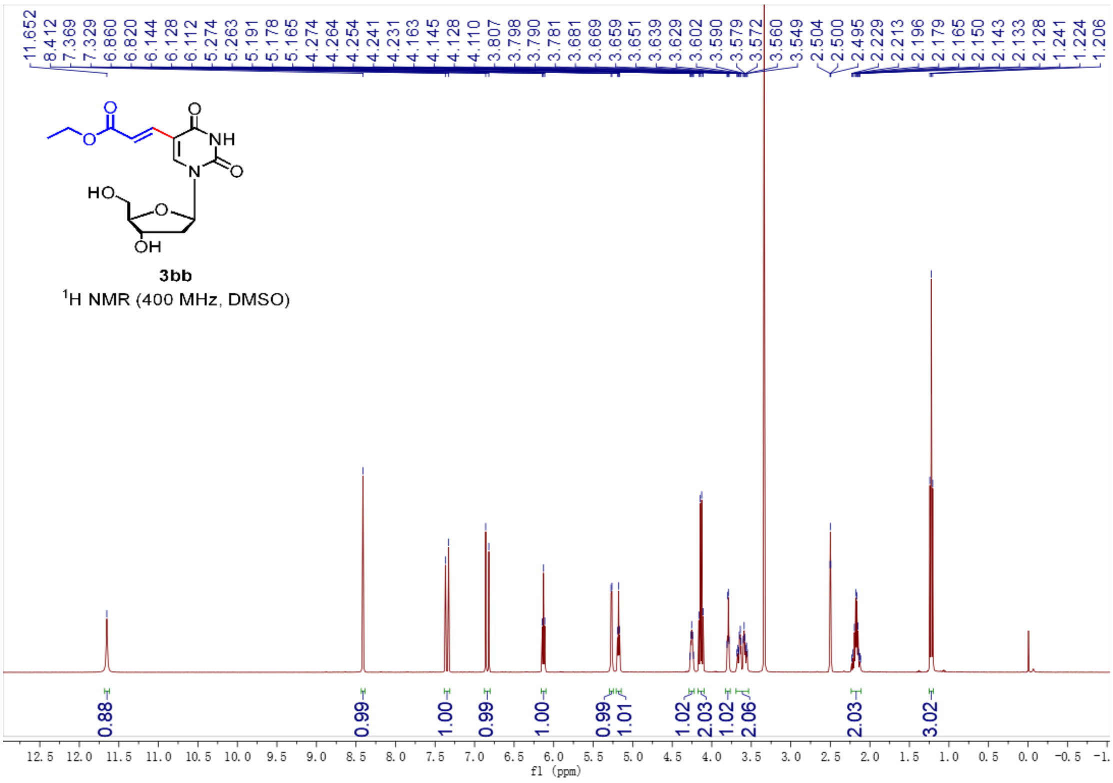

3bb

^{1}H NMR (400 MHz, DMSO)

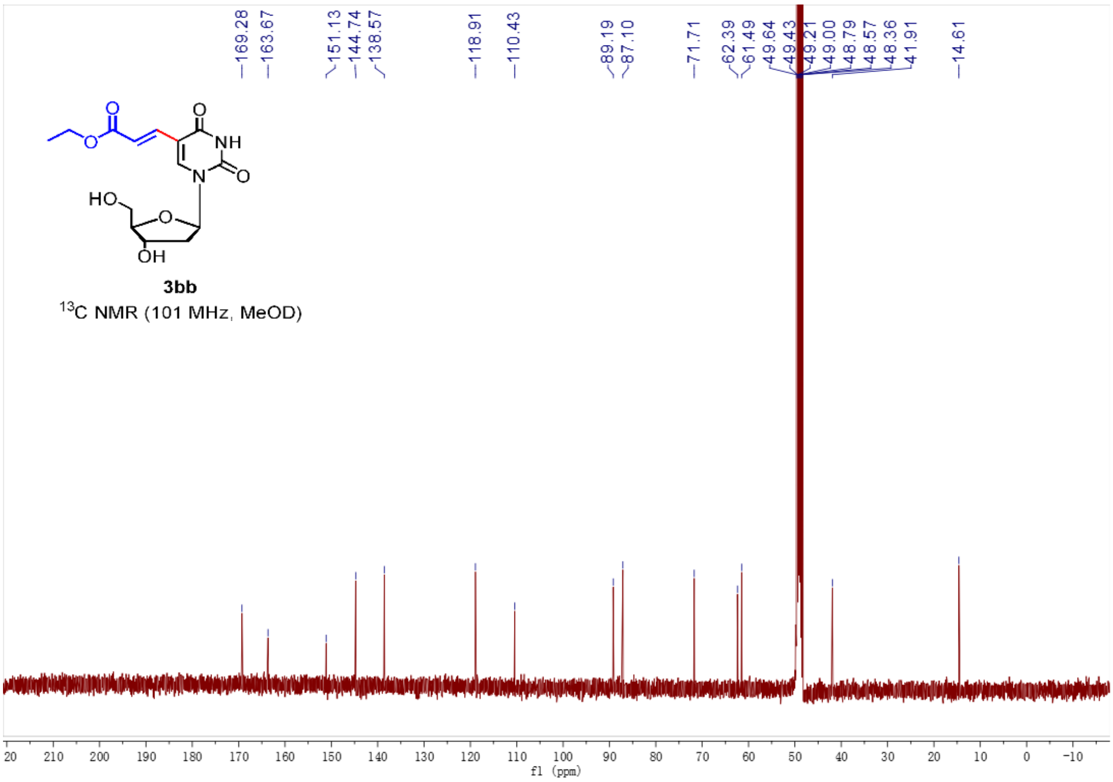

3bb

^{13}C NMR (101 MHz, MeOD)

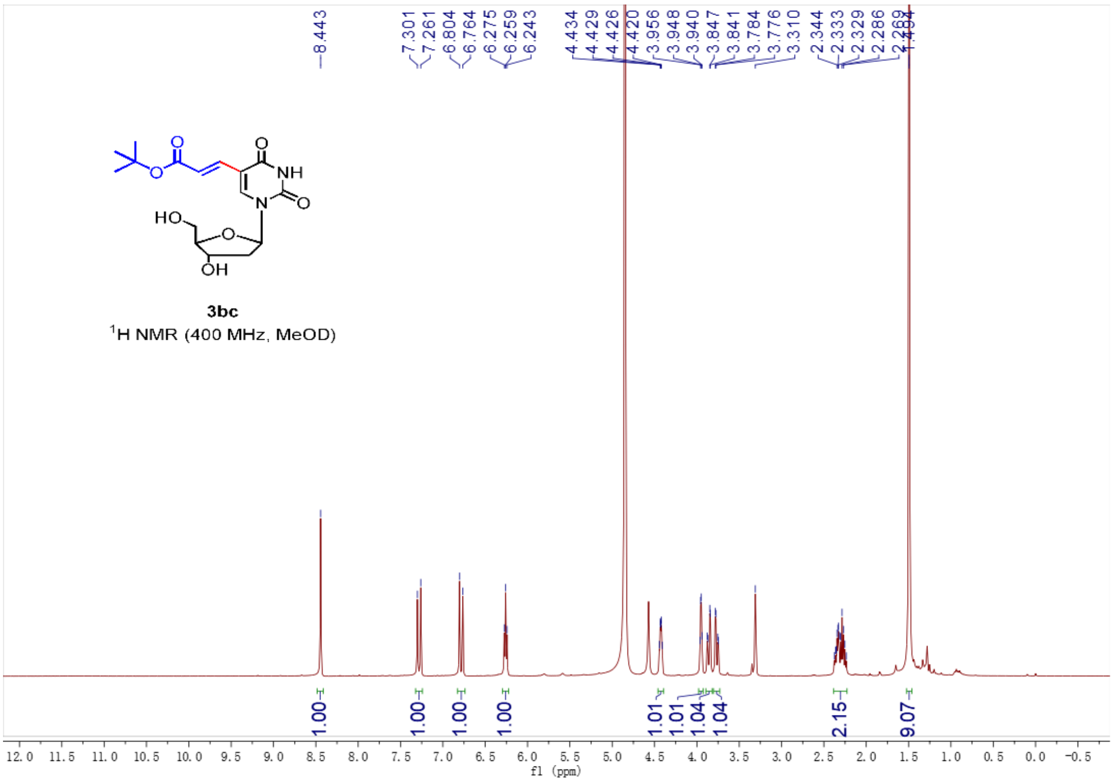
8.443
7.301
7.261
6.804
6.764
6.275
6.259
6.243
4.434
4.429
4.426
4.420
3.956
3.948
3.940
3.847
3.841
3.784
3.776
3.310
2.344
2.333
2.329
2.286
2.269
O
NH
N
O
O
O
HO
O
OH
3bc
¹H NMR (400 MHz, MeOD)
1.00
1.00
1.00
1.00
1.01
1.01
1.04
1.04
2.15
9.07
f1 (ppm)

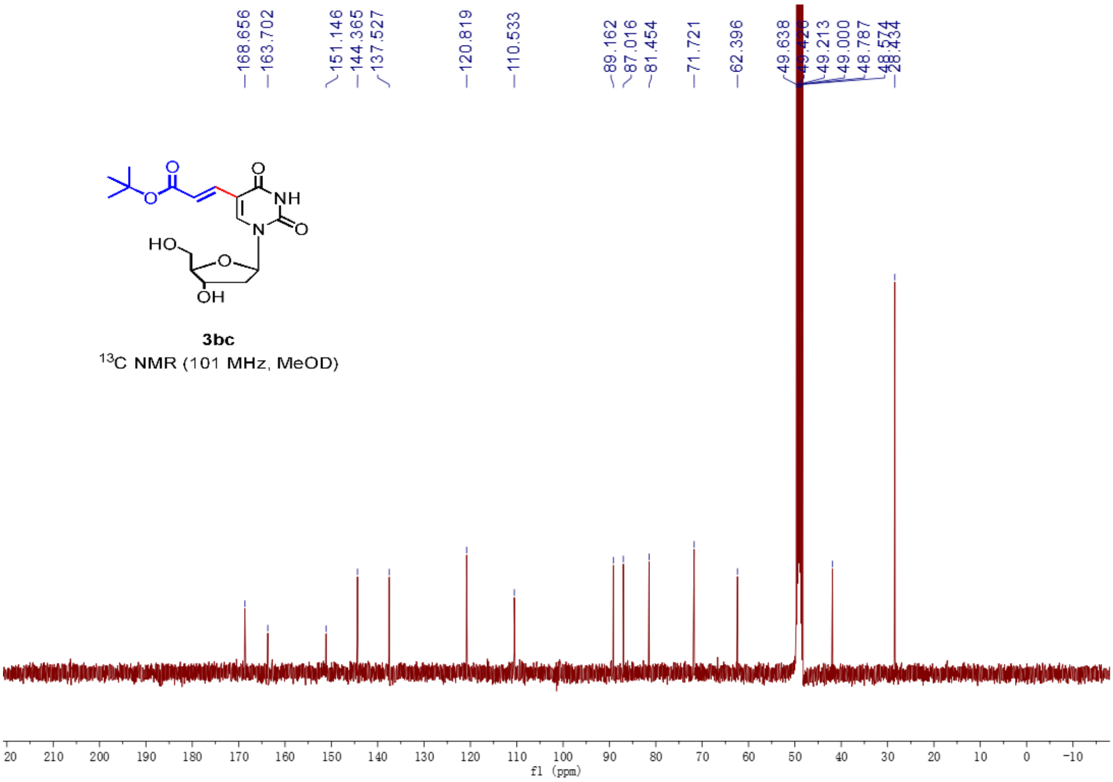
168.656
163.702
151.146
144.365
137.527
120.819
110.533
89.162
87.016
81.454
71.721
62.396
49.638
49.213
49.000
48.787
48.574
28.434
O
NH
N
O
O
O
HO
O
OH
3bc
¹³C NMR (101 MHz, MeOD)
f1 (ppm)

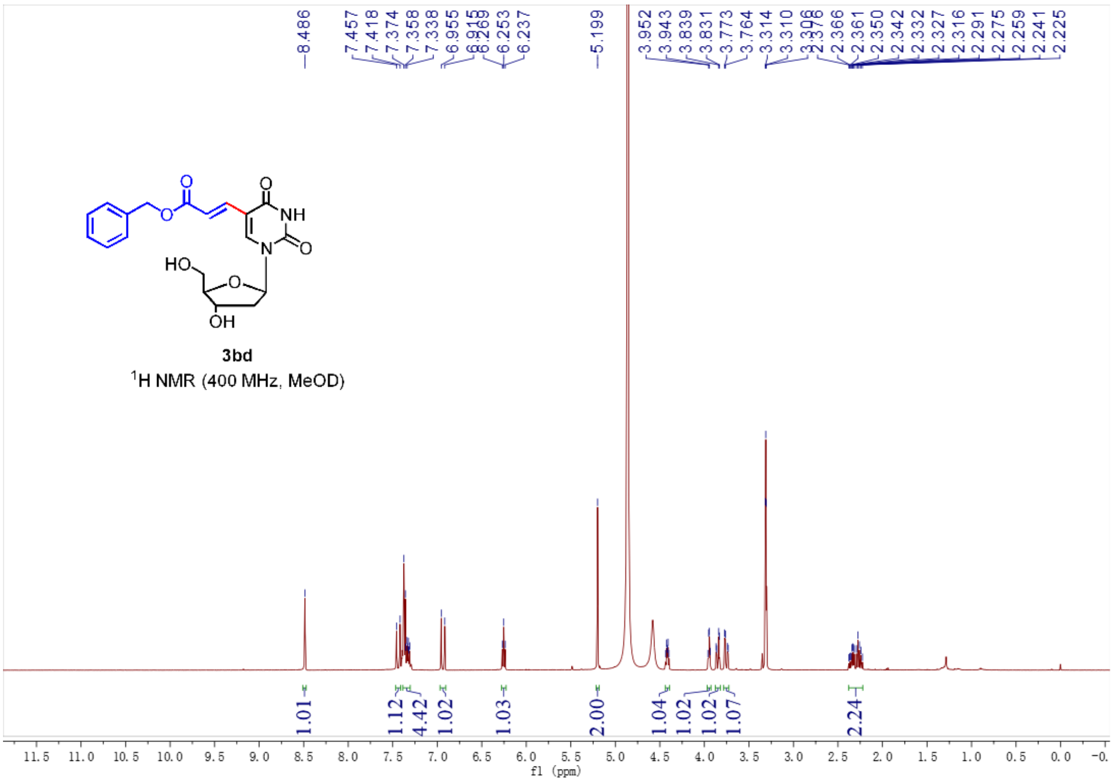
8.486
7.457
7.418
7.374
7.358
7.338
6.955
6.915
6.269
6.253
6.237
5.199
3.952
3.943
3.839
3.831
3.773
3.764
3.314
3.310
2.366
2.361
2.350
2.342
2.332
2.327
2.316
2.291
2.275
2.259
2.241
2.225
HO
O
OH
O
NH
N
O
O
O
3bd
1H NMR (400 MHz, MeOD)
1.01
1.12
4.42
1.02
1.03
2.00
1.04
1.02
1.02
1.07
2.24
f1 (ppm)

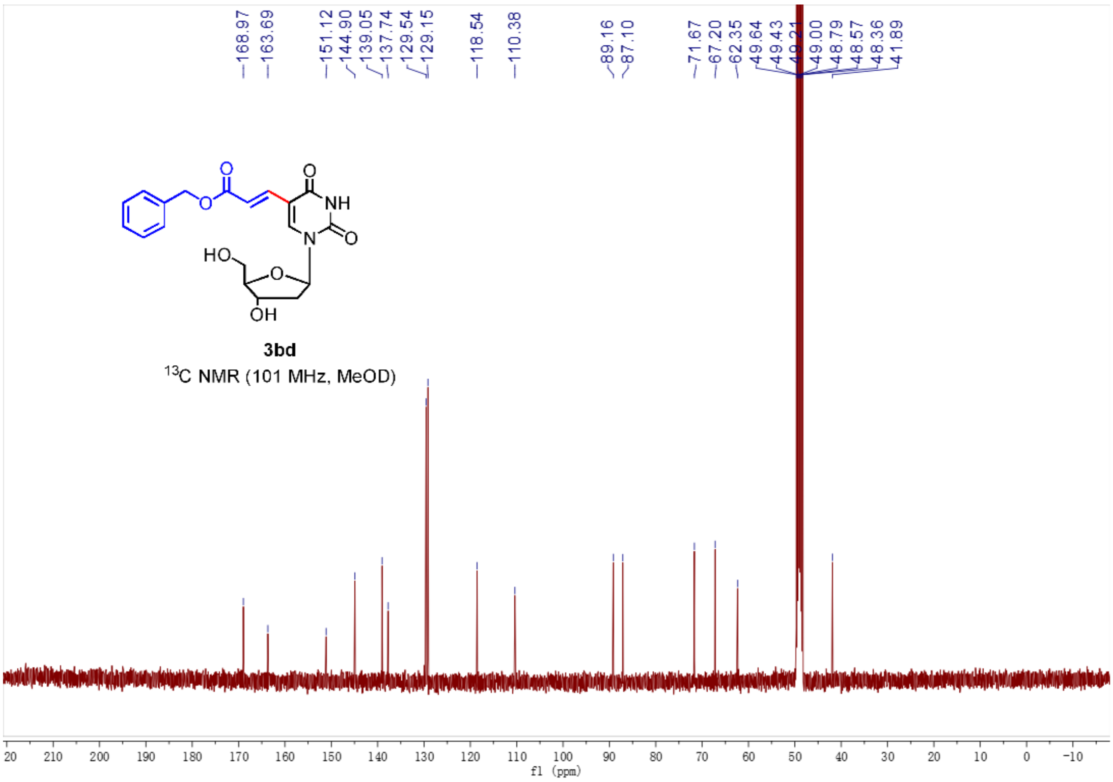
168.97
163.69
151.12
144.90
139.05
137.74
129.54
129.15
118.54
110.38
89.16
87.10
71.67
67.20
62.35
49.64
49.43
49.21
49.00
48.79
48.57
48.36
41.89
HO
O
OH
O
NH
N
O
O
O
3bd
13C NMR (101 MHz, MeOD)
f1 (ppm)

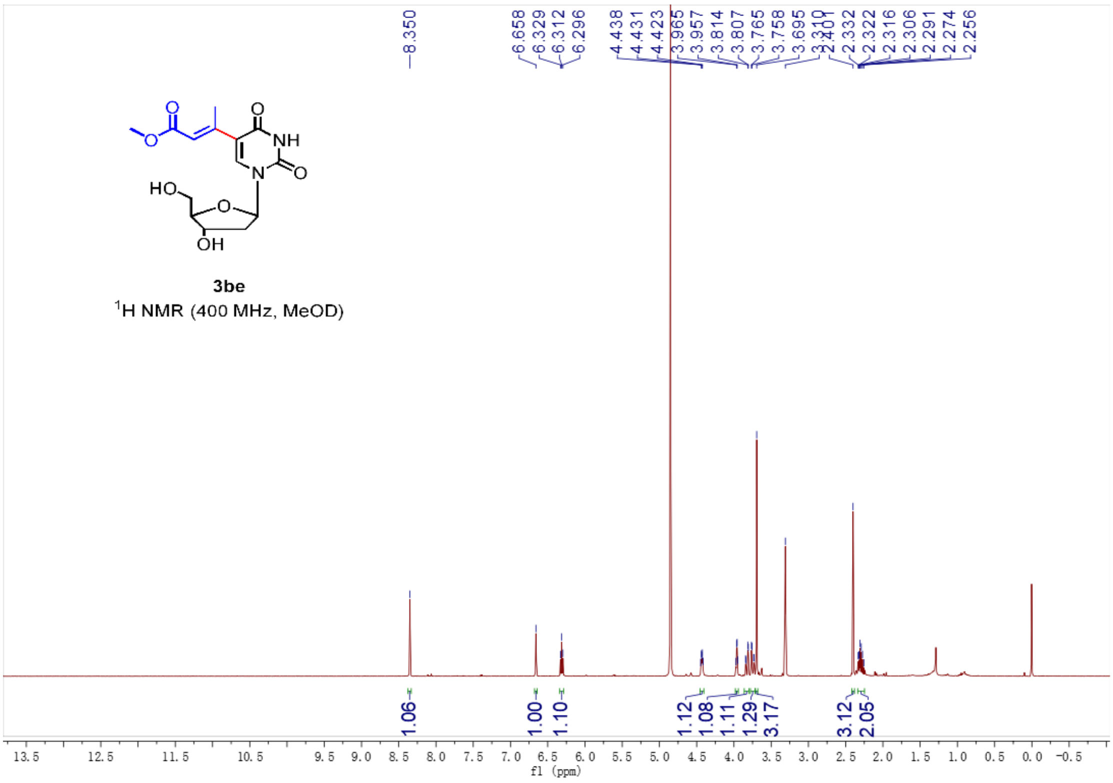

3be
1H NMR (400 MHz, MeOD)
8.350
6.658
6.329
6.312
6.296
4.438
4.431
4.423
3.965
3.957
3.814
3.807
3.765
3.758
3.695
3.310
2.401
2.332
2.322
2.316
2.306
2.291
2.274
2.256
1.06
1.00
1.10
1.12
1.08
1.11
1.29
3.17
3.12
2.05
f1 (ppm)

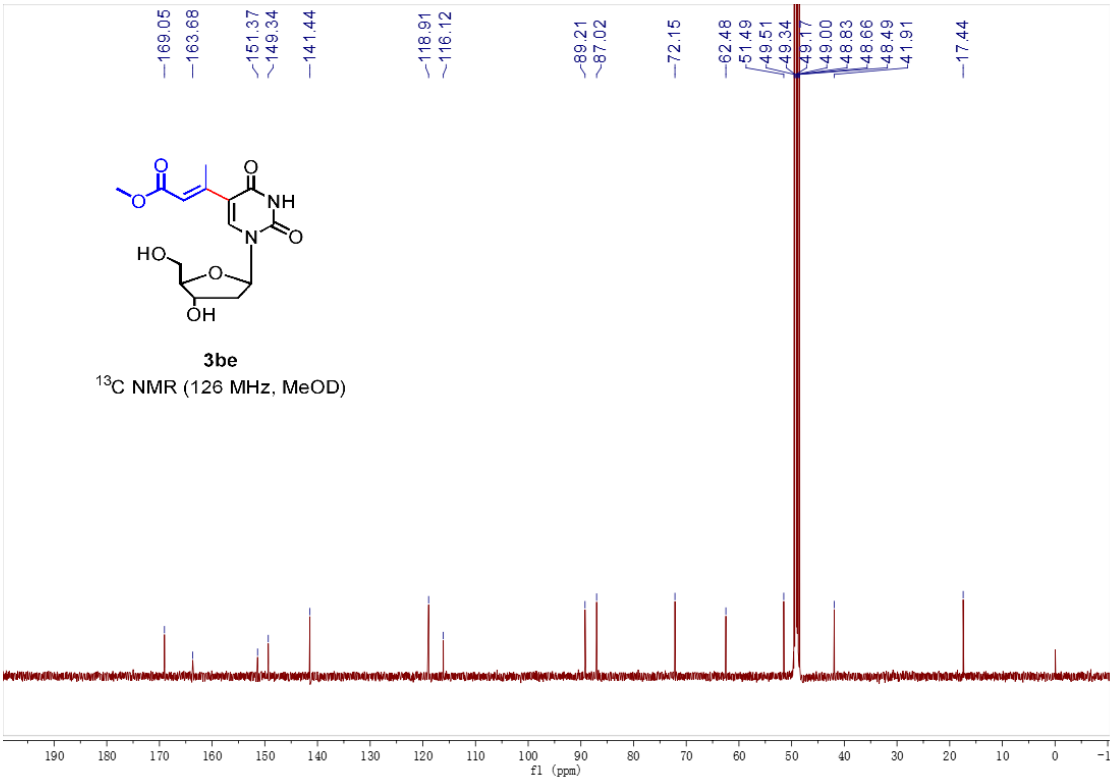

3be
13C NMR (126 MHz, MeOD)
169.05
163.68
151.37
149.34
141.44
118.91
116.12
89.21
87.02
72.15
62.48
51.49
49.51
49.34
49.17
49.00
48.83
48.66
48.49
41.91
17.44
f1 (ppm)

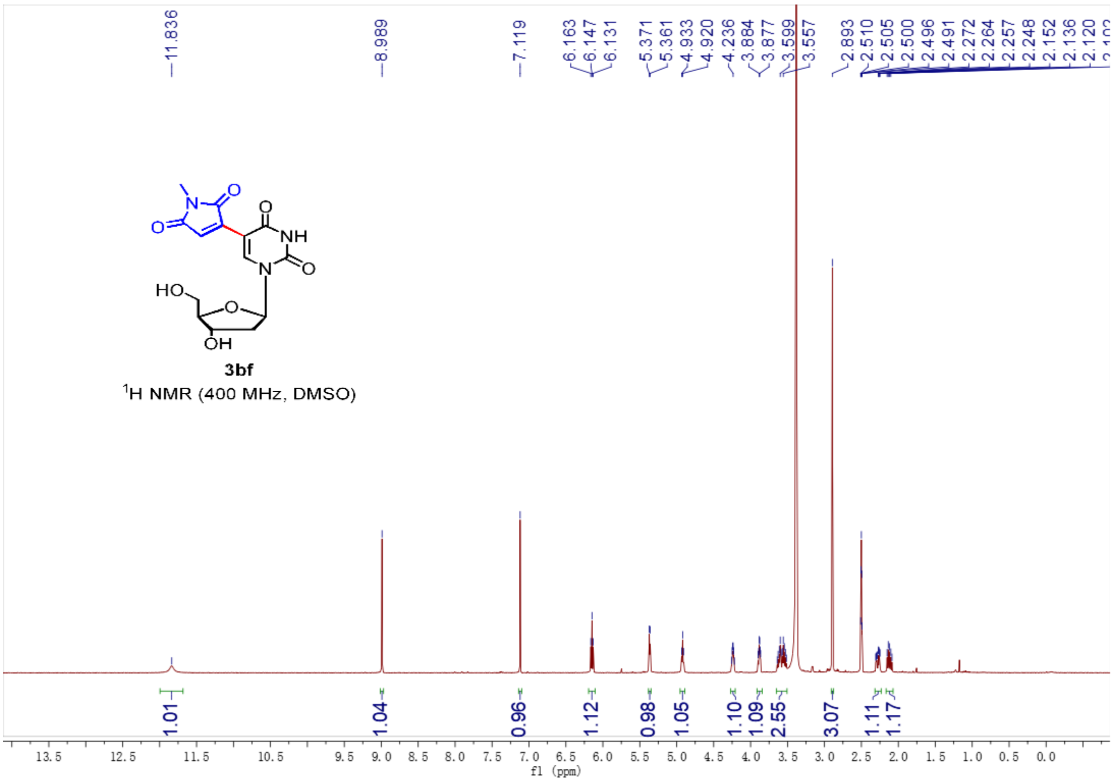
3bf
^{1}H NMR (400 MHz, DMSO)
f1 (ppm)

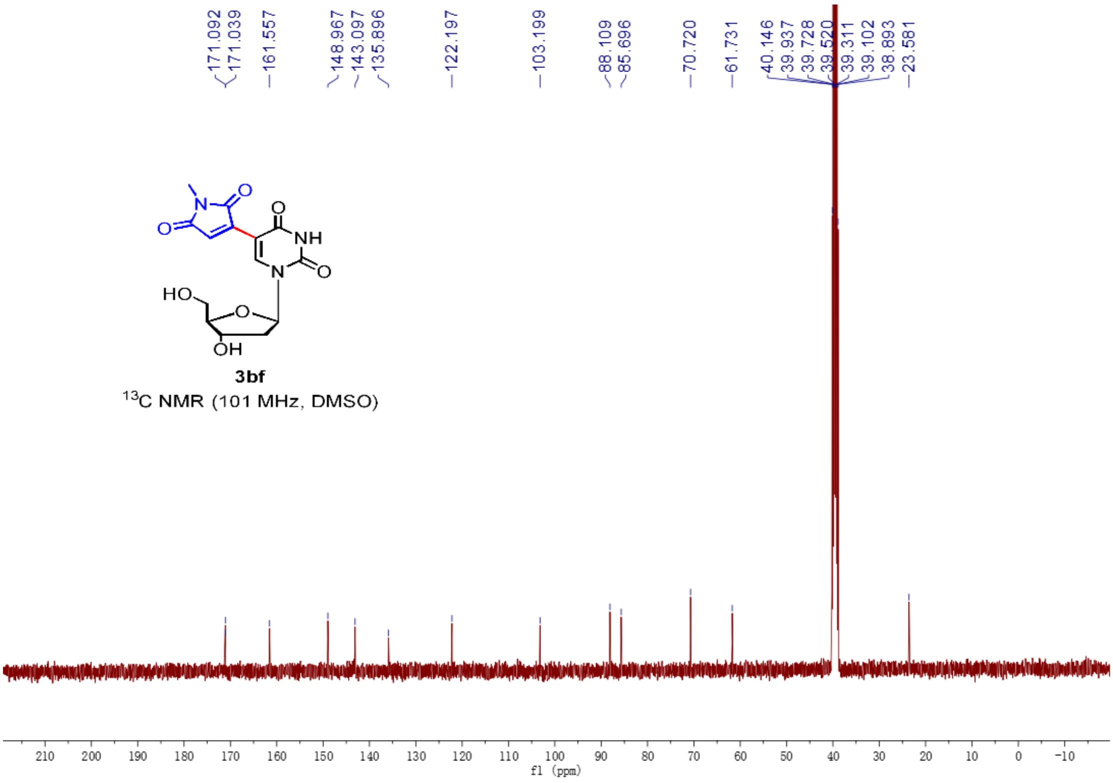
3bf
^{13}C NMR (101 MHz, DMSO)
f1 (ppm)

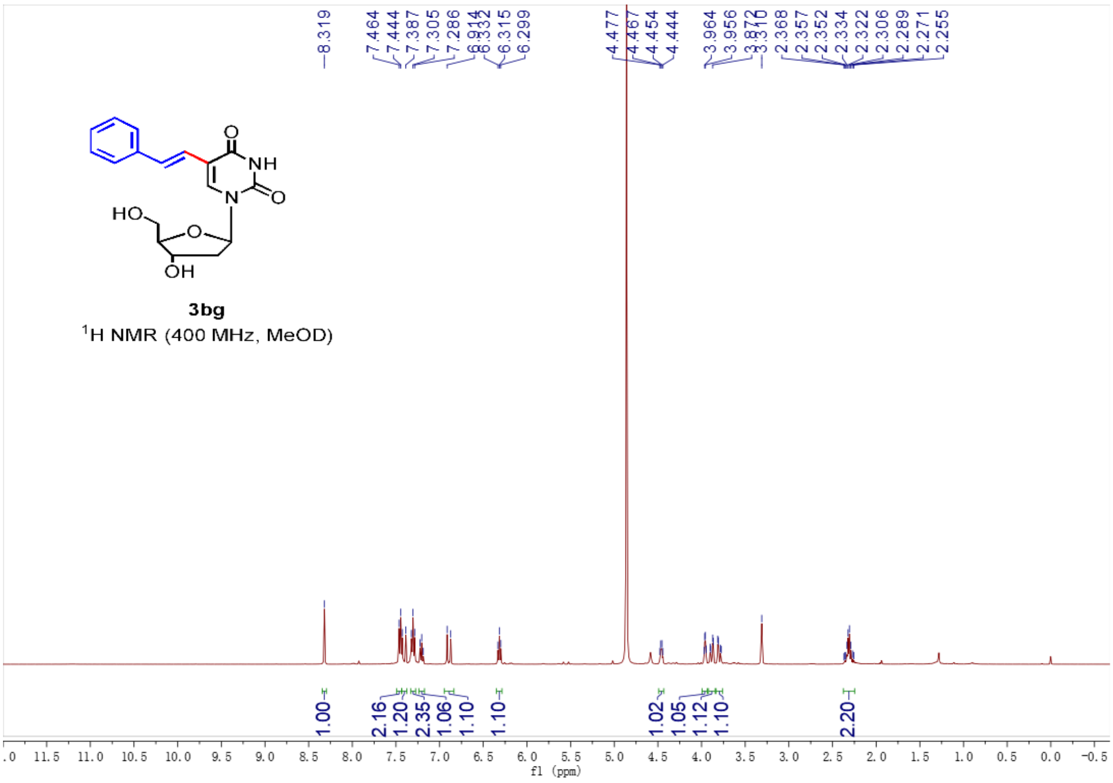
3bg
1H NMR (400 MHz, MeOD)
f1 (ppm)

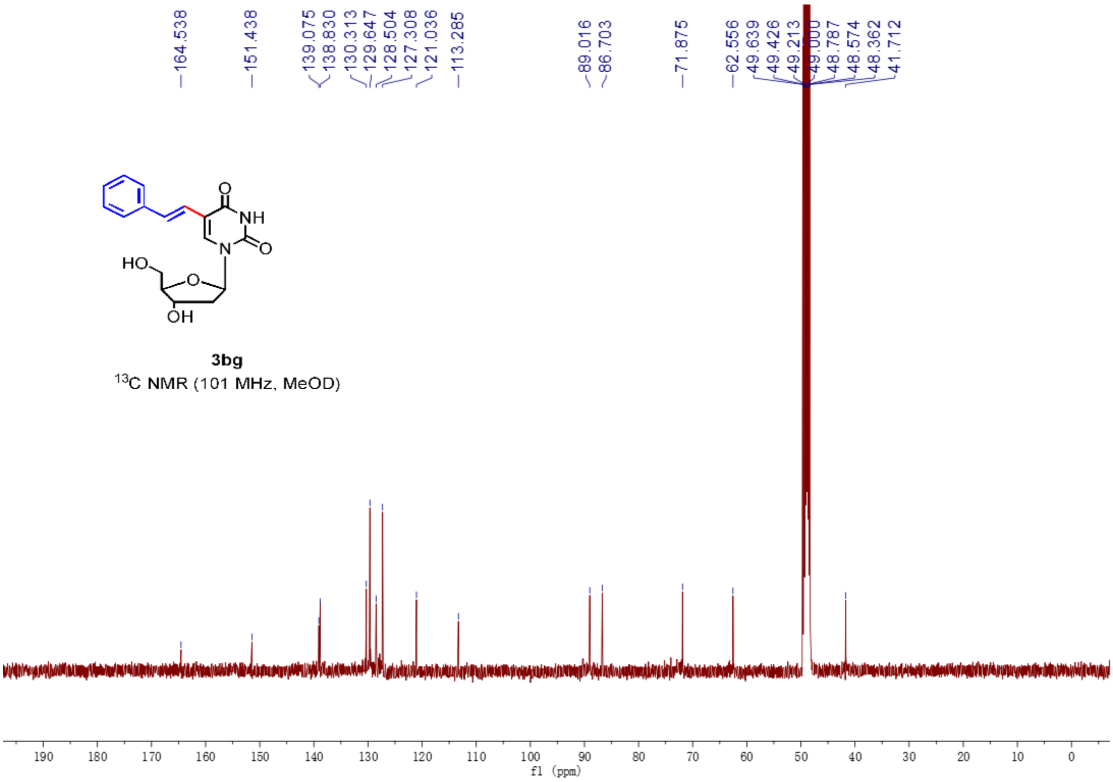
3bg
13C NMR (101 MHz, MeOD)
f1 (ppm)

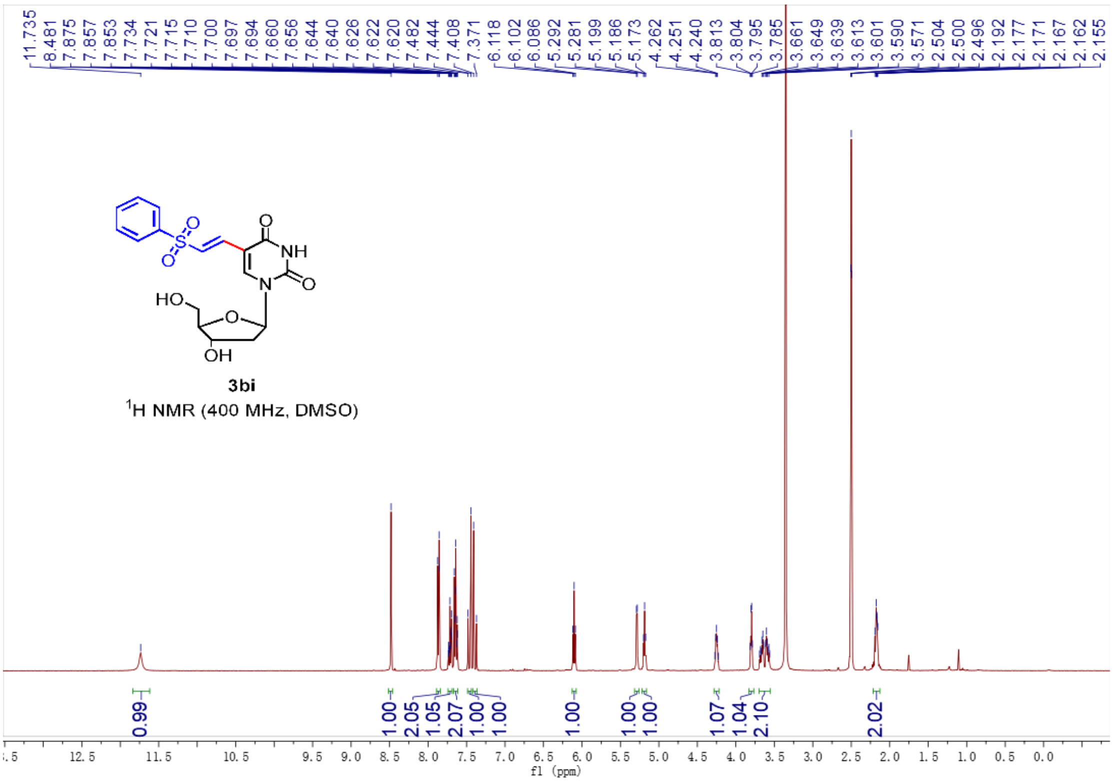
3bi
^{1}H NMR (400 MHz, DMSO)
f1 (ppm)

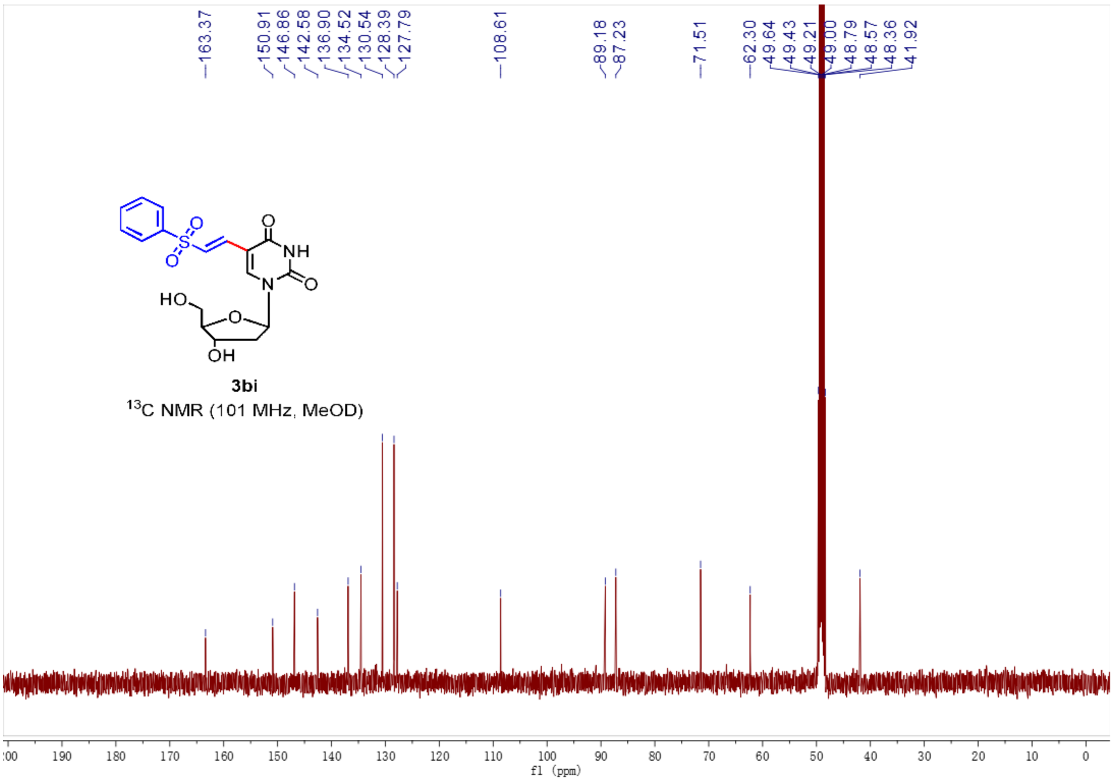
3bi
^{13}C NMR (101 MHz, MeOD)
f1 (ppm)

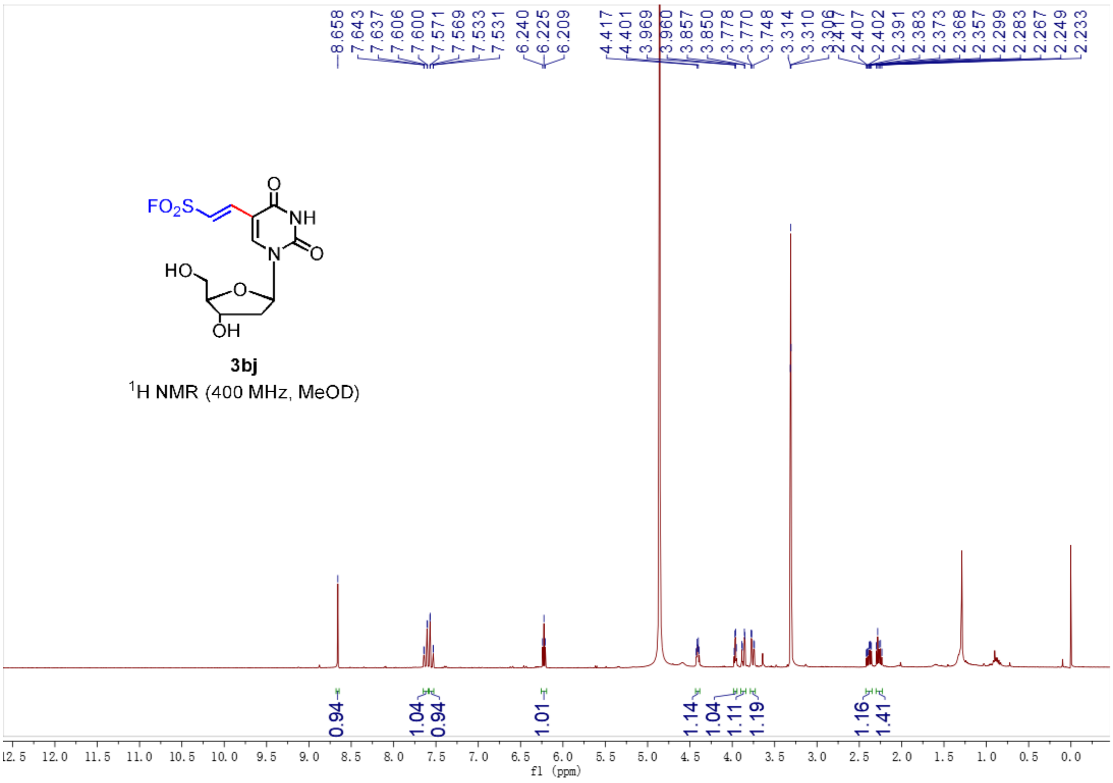
FO2S
NH
O
N
HO
OH
3bj
1H NMR (400 MHz, MeOD)
f1 (ppm)

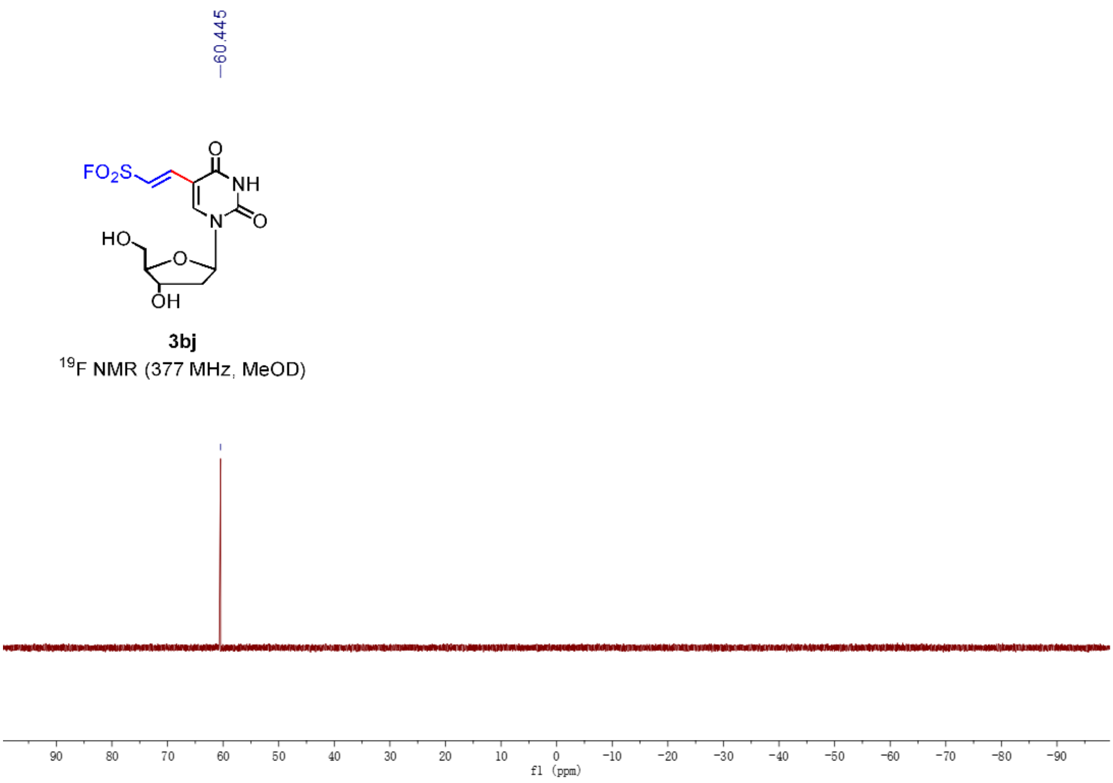
-60.445
FO2S
NH
O
N
HO
OH
3bj
19F NMR (377 MHz, MeOD)
f1 (ppm)

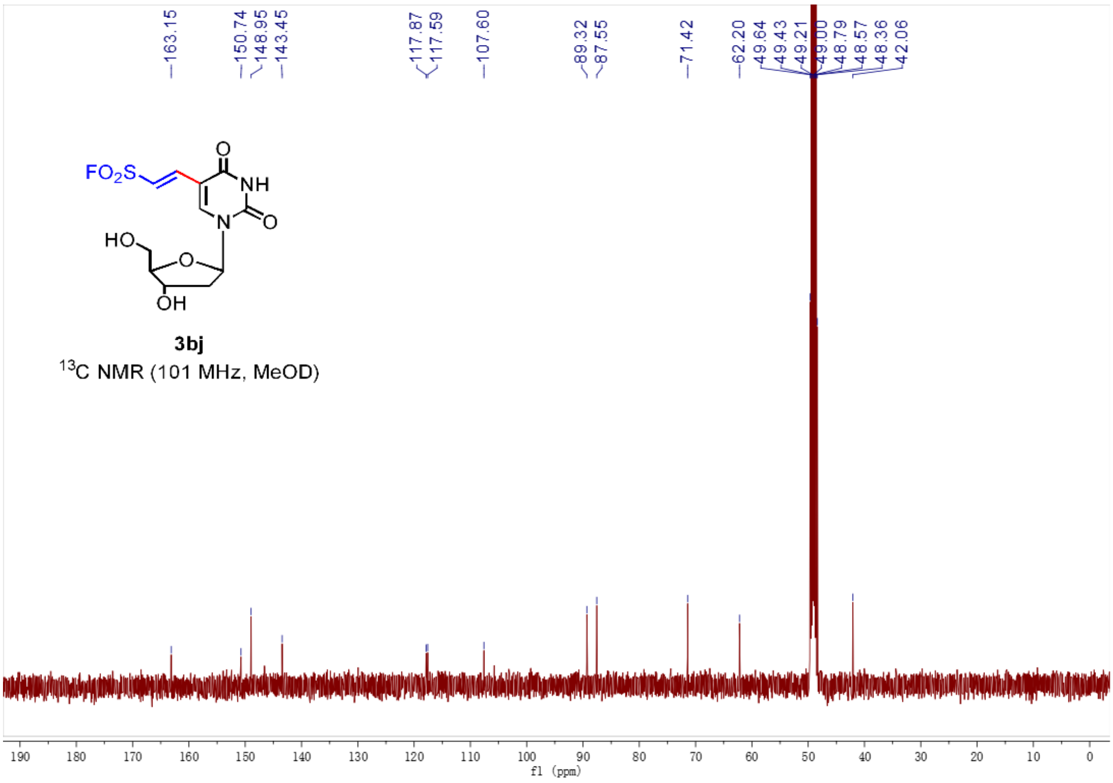
163.15
150.74
148.95
143.45
117.87
117.59
107.60
89.32
87.55
71.42
62.20
49.64
49.43
49.21
49.00
48.79
48.57
48.36
42.06
FO2S
O
NH
N
O
HO
O
OH
3bj
13C NMR (101 MHz, MeOD)
f1 (ppm)

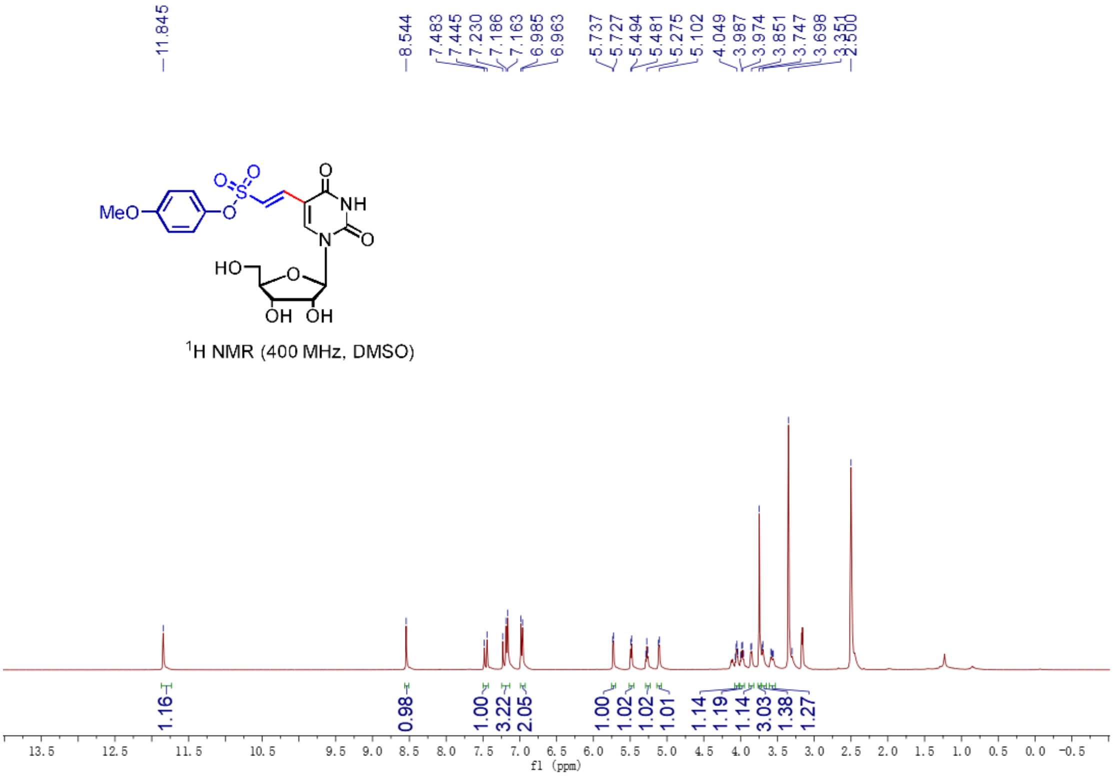
11.845
8.544
7.483
7.445
7.230
7.186
7.163
6.985
6.963
5.737
5.727
5.494
5.481
5.275
5.102
4.049
3.987
3.974
3.851
3.747
3.698
3.351
2.500
MeO
O
S
O
O
NH
N
O
HO
O
OH OH
1H NMR (400 MHz, DMSO)
1.16
0.98
1.00
3.22
2.05
1.00
1.02
1.02
1.01
1.14
1.19
1.14
3.03
1.38
1.27
f1 (ppm)

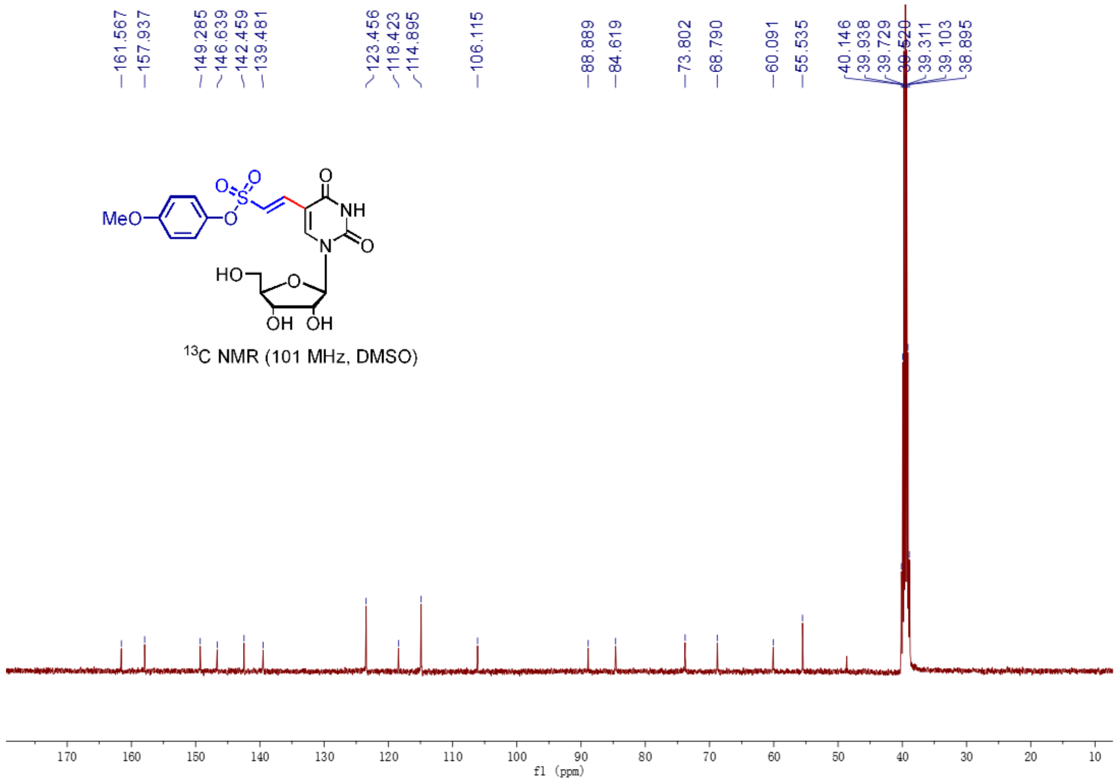

161.567
157.937
149.285
146.639
142.459
139.481
123.456
118.423
114.895
106.115
88.889
84.619
73.802
68.790
60.091
55.535
40.146
39.938
39.729
39.520
39.311
39.103
38.895
MeO
O
S
O
O
NH
N
O
O
HO
O
OH
OH
^{13}C NMR (101 MHz, DMSO)
170
160
150
140
130
120
110
100
90
80
70
60
50
40
30
20
10
f1 (ppm)
